# Supplementary figures and images for: Development and Evaluation of AccuPower COVID-19 Multiplex Real-Time RT-PCR Kit and AccuPower SARS-CoV-2 Multiplex Real-Time RT-PCR Kit for SARS-CoV-2 Detection in Sputum, NPS/OPS, Saliva and Pooled Samples
Source: PLoS One. 2022 Feb 10;17(2):e0263341. doi: 10.1371/journal.pone.0263341 (PMC8830688; doi:10.1371/journal.pone.0263341)

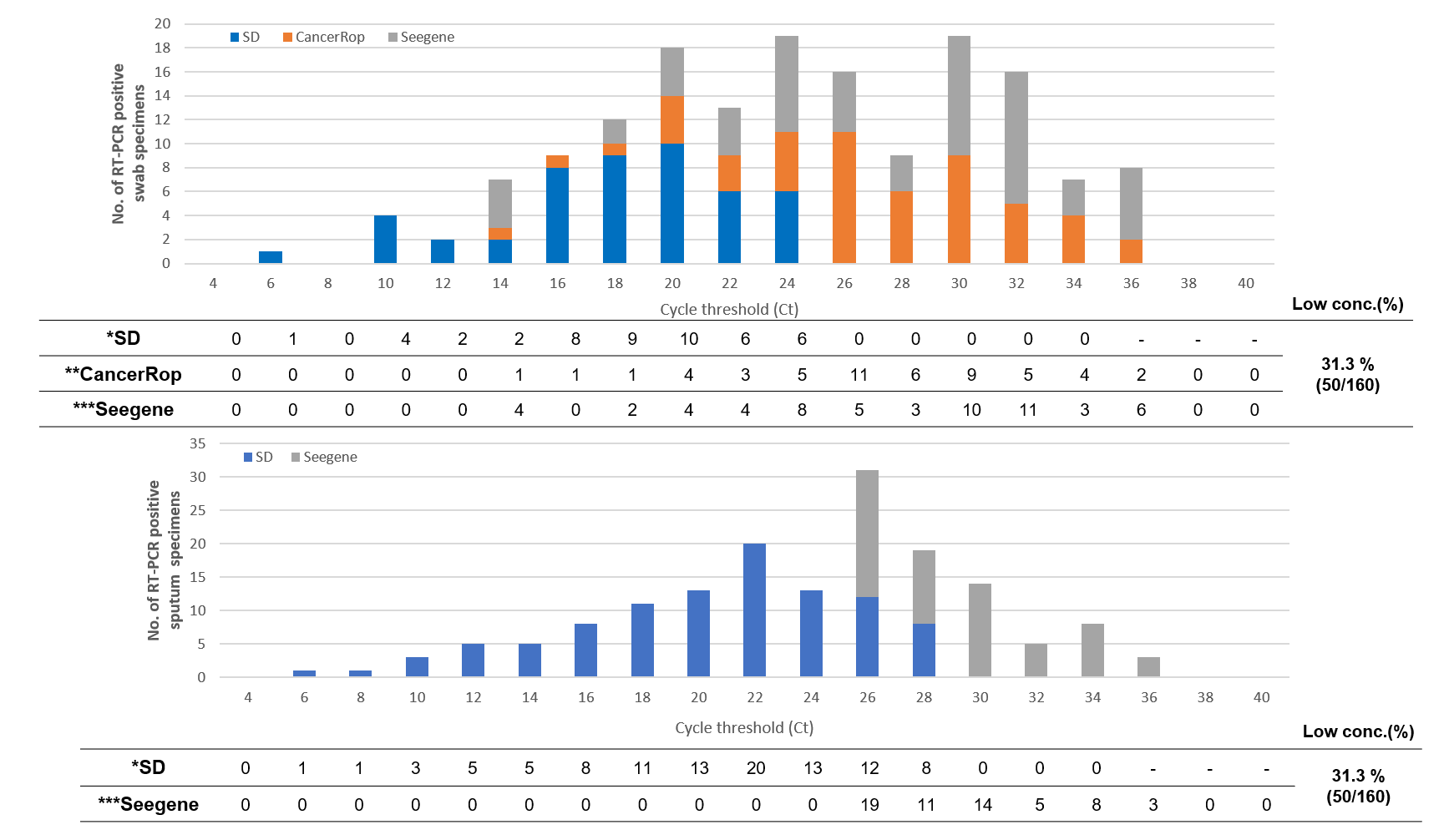

Supplement: S1 Fig — Nashpharyngeal and Oropharyngeal swab specimens Ct distribution (Above). Sputum specimens Ct distribution (Below). *SD: STANDARD™ M nCoV Real-Time Detection kit, Cutoff (Ct): 36 **CancerRop: Q-Sens® COVID-19 Detection Kit V2, Cutoff (Ct): 40 ***Seegene: Allplex™ 2019-nCoV Assay, Cutoff (Ct): 40 (TIF) [file pone.0263341.s001.tif]

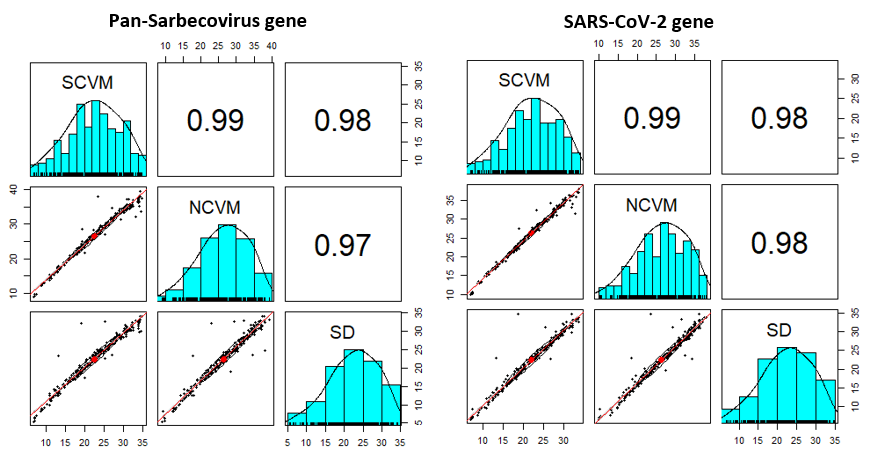

Supplement: S2 Fig — Ct values among assays (NCVM, SCVM and STANDARD™ M nCoV Real-Time Detection kit) showed high correlation with a Pearson R2 correlation coefficient ≥0.97. (TIF) [file pone.0263341.s002.tif]

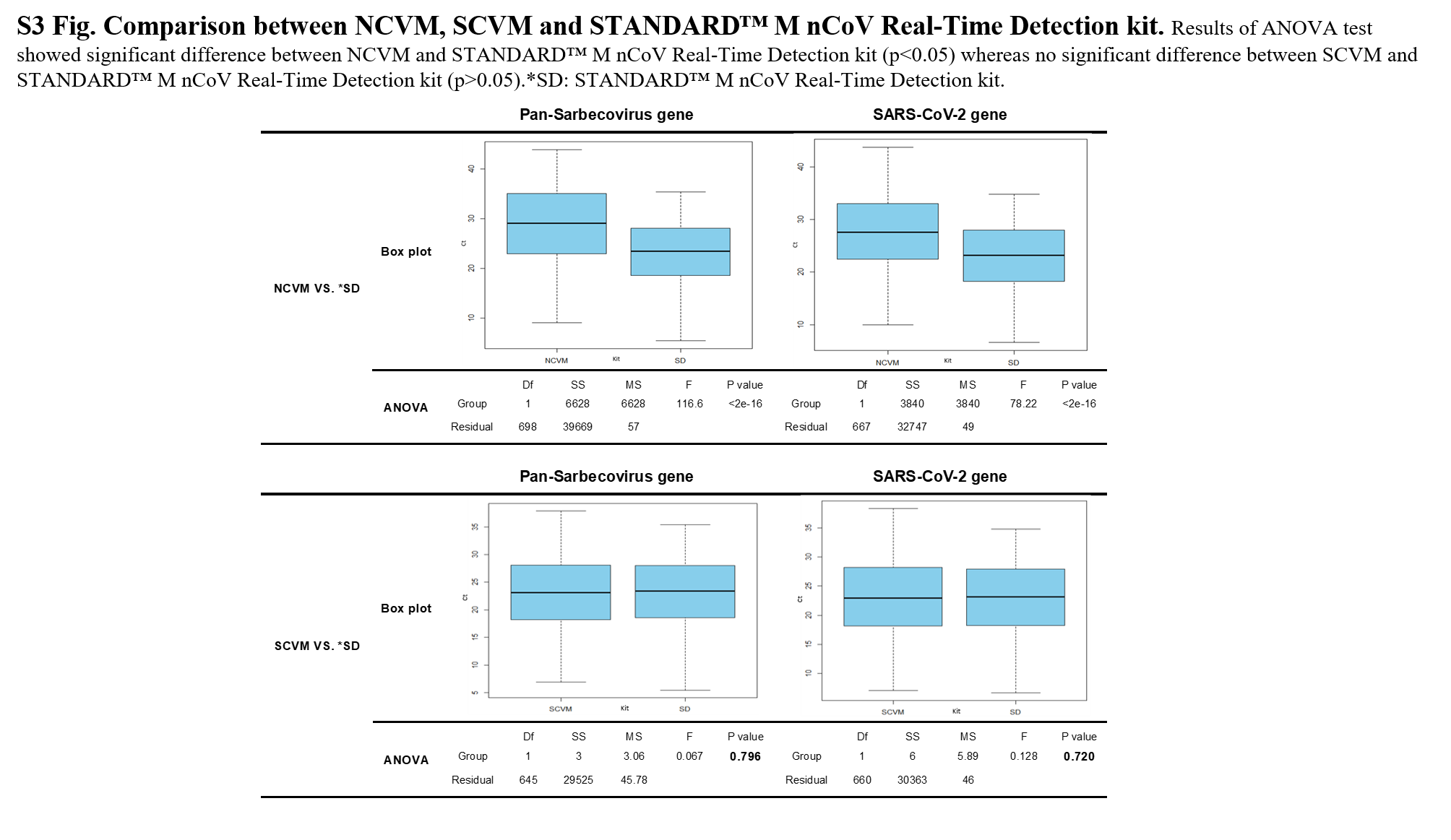

Supplement: S3 Fig — Results of ANOVA test showed significant difference between NCVM and STANDARD™ M nCoV Real-Time Detection kit (p<0.05) whereas no significant difference between SCVM and STANDARD™ M nCoV Real-Time Detection kit (p>0.05).*SD: STANDARD™ M nCoV Real-Time Detection kit. (TIF) [file pone.0263341.s003.tif]

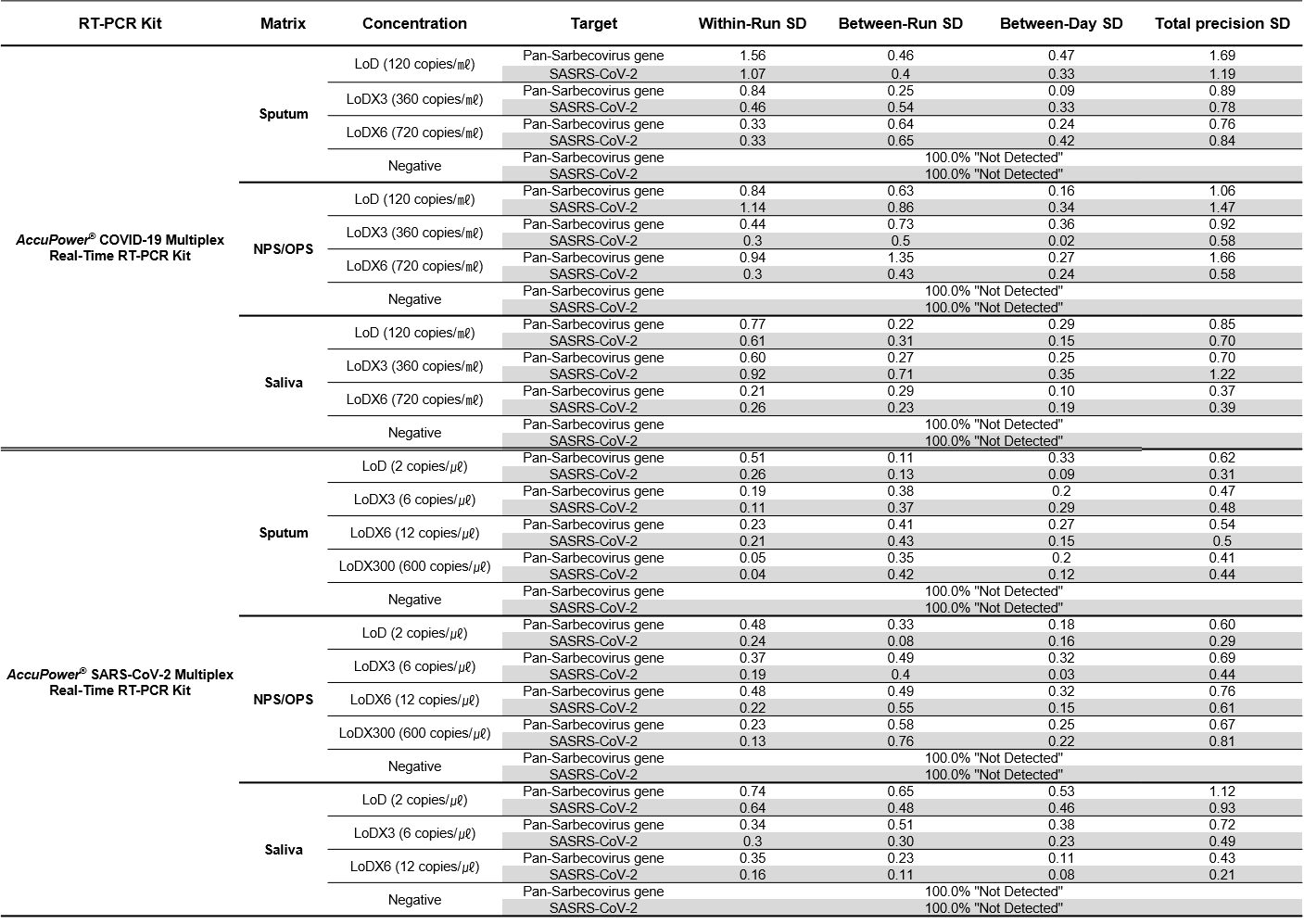

Supplement: S1 Table — Precision evaluation results of AccuPower® COVID-19 Multiplex Real-Time RT-PCR Kit and AccuPower® SARS-CoV-2 Multiplex Real-Time RT-PCR Kit. (TIF) [file pone.0263341.s004.tif]

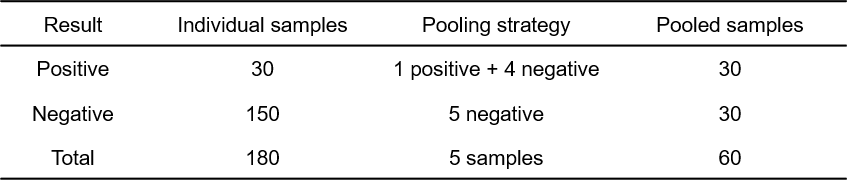

Supplement: S2 Table — (TIF) [file pone.0263341.s005.tif]

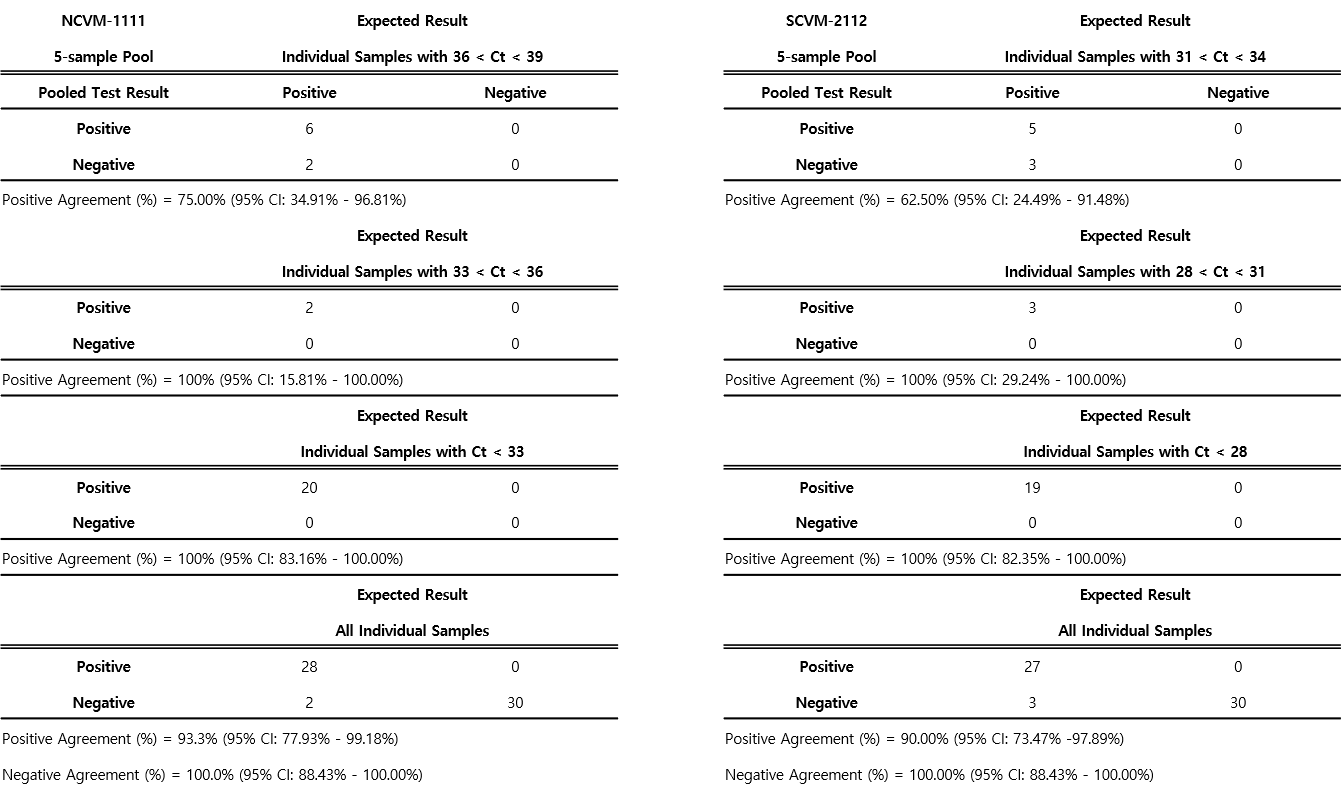

Supplement: S3 Table — (TIF) [file pone.0263341.s006.tif]

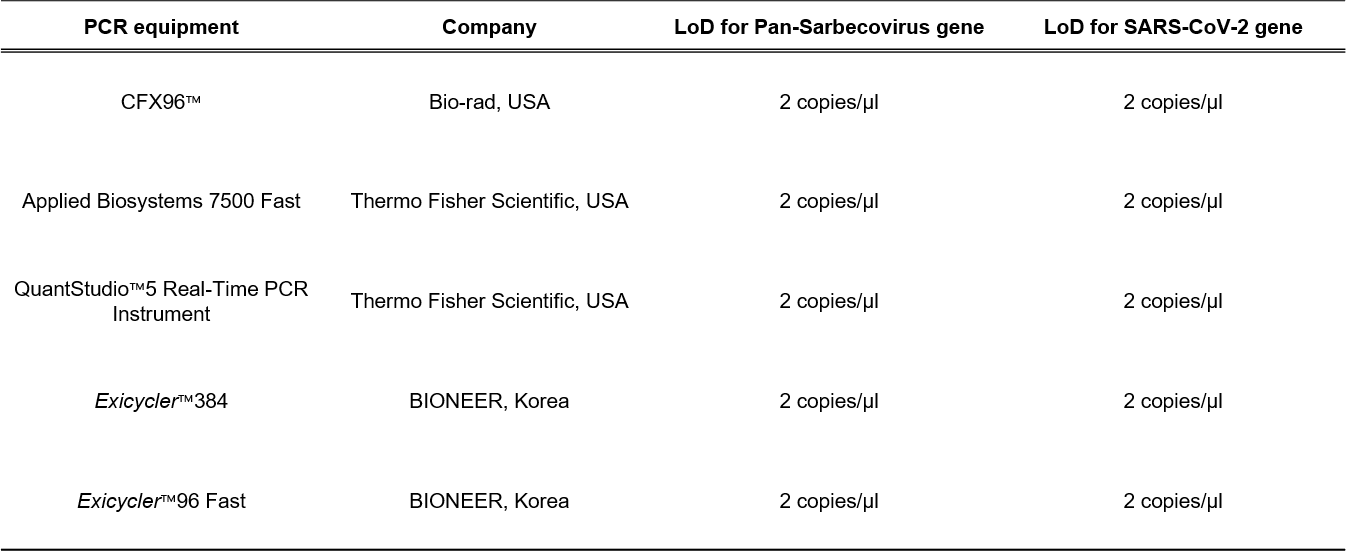

Supplement: S4 Table — (TIF) [file pone.0263341.s007.tif]

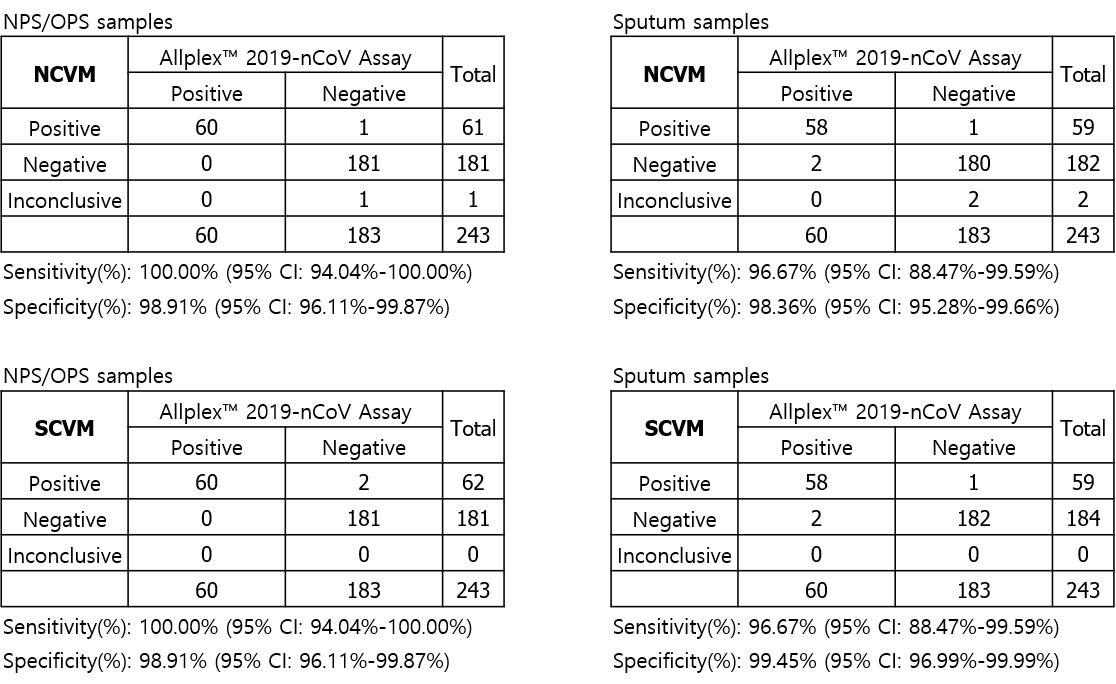

Supplement: S5 Table — (TIF) [file pone.0263341.s008.tif]
