# Supplementary material for: Development and Evaluation of AccuPower COVID-19 Multiplex Real-Time RT-PCR Kit and AccuPower SARS-CoV-2 Multiplex Real-Time RT-PCR Kit for SARS-CoV-2 Detection in Sputum, NPS/OPS, Saliva and Pooled Samples
Source: PLoS One. 2022 Feb 10;17(2):e0263341. doi: 10.1371/journal.pone.0263341 (PMC8830688; doi:10.1371/journal.pone.0263341)
Supplement: S1 Data Analytical Performance Evaluation — (PDF) [file pone.0263341.s009.pdf]

Table 2. Characteristic and PCR condition for LoD test of the *AccuPower*® Kits

1. *AccuPower*® COVID-19 Multiplex Real-Time RT-PCR Kit (NCVM)

LoD- Hit Rate Analysis

Lot	Matrix	Nominal Concentration (copies/㎖)	Number of replicates tested (N)	Pan-Sarbecovirus gene		SARS-CoV-2 gene	
				Number of positives detected (N)	Positive rate (%)	Number of positives detected (N)	Positive rate (%)
200111H	Sputum	240	20	20	100%	20	100%
		200	20	20	100%	20	100%
		160	20	20	100%	20	100%
		120	20	20	100%	20	100%
		80	20	18	90%	18	90%
		40	20	13	65%	9	45%
		0	20	0	0%	0	0%
	Swab	240	20	20	100%	20	100%
		200	20	20	100%	20	100%
		160	20	20	100%	20	100%
		120	20	19	95%	19	95%
		80	20	18	90%	18	90%
		40	20	18	90%	18	90%
		0	20	0	0%	0	0%
	Saliva	240	20	20	100%	20	100%
		200	20	20	100%	20	100%
		160	20	20	100%	20	100%
		120	20	20	100%	19	95%
		80	20	20	100%	18	90%
		40	20	18	90%	18	90%
		0	20	1	5%	0	0%

Lot	Matrix	Nominal Concentration (copies/㎖)	Number of replicates tested (N)	Pan-Sarbecovirus gene		SARS-CoV-2 gene	
				Number of positives detected (N)	Positive rate (%)	Number of positives detected (N)	Positive rate (%)
200211H	Sputum	240	20	20	100%	20	100%
		200	20	20	100%	20	100%
		160	20	20	100%	20	100%
		120	20	19	95%	20	100%
		80	20	18	90%	18	90%
		40	20	16	80%	14	70%
		0	20	0	0%	0	0%
	Swab	240	20	20	100%	20	100%
		200	20	20	100%	20	100%
		160	20	20	100%	20	100%
		120	20	20	100%	20	100%
		80	20	18	90%	18	90%
		40	20	15	75%	17	85%
		0	20	0	0%	0	0%
	Saliva	240	20	20	100%	20	100%
		200	20	20	100%	20	100%
		160	20	20	100%	20	100%
		120	20	19	95%	19	95%
		80	20	18	90%	19	95%
		40	20	17	85%	17	85%
		0	20	0	0%	0	0%

[Limit of Detection of the *AccuPower*® COVID-19 Multiplex Real-Time RT-PCR Kit]

Matrix	Instrument	Pan-Sarbecovirus gene	SARS-CoV-2 gene
Sputum	*ExiStation*™48 system	120 copies/㎖	120 copies/㎖
Swab	*ExiStation*™48 system	120 copies/㎖	120 copies/㎖
Saliva	*ExiStation*™48 system	120 copies/㎖	120 copies/㎖

Result of Ct value in LoD study

1 200111H Lot

Lot	Matrix	Sputum																				
	Repl	Neg			240 copies/㎖			200 copies/㎖			160 copies/㎖			120 copies/㎖			80 copies/㎖			40 copies/㎖		
		IPC	Pan-Sarbecovirus gene	SARS-CoV-2	IPC	Pan-Sarbecovirus gene	SARS-CoV-2	IPC	Pan-Sarbecovirus gene	SARS-CoV-2	IPC	Pan-Sarbecovirus gene	SARS-CoV-2	IPC	Pan-Sarbecovirus gene	SARS-CoV-2	IPC	Pan-Sarbecovirus gene	SARS-CoV-2	IPC	Pan-Sarbecovirus gene	SARS-CoV-2
200111H	1	25.83	N.D	N.D	28.93	38.2	36.23	29.99	38.7	36.9	27.76	36.16	36.65	28.39	37	36.35	27.73	37.19	36.83	28.4	ND	ND
	2	25.67	N.D	N.D	29.22	37.45	36.23	29.72	37.6	36.46	28.45	37.76	37.35	28.15	37.36	36.47	28.4	ND	ND	28.09	39.51	ND
	3	25.77	N.D	N.D	29.17	36.61	35.88	28.48	36.53	35	28.49	37.42	37.39	28.37	37.41	36.32	28.51	37.75	37.27	27.55	38.92	ND
	4	25.68	N.D	N.D	29.19	37.35	35.76	28.4	37.05	35.62	28.06	37.19	36.59	28.35	36.74	36.5	28.58	38.21	37.43	28.65	35.03	38.8
	5	27.17	N.D	N.D	29.76	38.04	36.44	30.5	38.52	37.13	29.27	34.93	37.57	29.18	38.17	36.91	29.12	38.83	37.91	29.42	35.03	39.3
	6	27.06	N.D	N.D	29.94	37.3	36.44	30.38	37.45	36.69	28.84	37.28	37.28	29.18	38.06	37.4	29.47	39.66	38.05	29.2	ND	39.55
	7	27.11	N.D	N.D	29.84	36.44	36.11	29.23	36.38	35.2	29.01	37.98	37.36	29.22	37.72	37.29	29.48	39.98	38.07	29.16	40.47	39.71
	8	26.46	N.D	N.D	29.92	37.21	36.02	29.22	36.87	35.88	29.1	38.41	37.4	29.27	36.5	36.92	29.52	39.22	37.82	29.39	40.48	ND
	9	26.32	N.D	N.D	28.47	35.44	36.19	29.09	36.27	36.01	30	39.3	37.43	28.68	38.18	36.87	29.7	39.25	38.42	32.37	ND	39.67
	10	26.28	N.D	N.D	29.18	37.03	36.93	28.82	36.72	36.14	29.16	39.08	37.77	35.38	38.3	38.8	30.24	40.4	38.16	30.01	40.57	ND
	11	26.75	N.D	N.D	29.19	36.76	37.02	29.06	36.69	36.05	29.03	39.08	38.32	29.78	38.55	37.59	29.37	39.26	37.9	29.65	ND	39.11
	12	27.29	N.D	N.D	28.71	36.45	36.19	29.08	36.04	36.21	29.57	39.09	37.56	29.77	39.27	37.29	30.92	ND	39.26	29.7	41.47	ND
	13	26.15	N.D	N.D	28.66	36.9	36.28	28.41	37	36.6	28.25	38.05	36.02	28.74	38.51	36.68	29.01	38.99	37.35	28.75	38.96	ND
	14	26.67	N.D	N.D	28.52	37.1	35.95	27.53	38.28	36.68	28.25	37.8	36.31	28.53	38.53	36.8	28.86	39.2	ND	27.96	40.48	ND
	15	25.86	N.D	N.D	28.58	34.57	36.22	27.99	36.97	36.9	28.53	37.44	37.07	28.65	38.62	36.98	28.94	36.87	37.34	28.29	38.78	37.94
	16	27.03	N.D	N.D	28.36	37.15	36.26	28.31	36.94	36.6	28.91	38.25	36.37	28.22	37.82	36.73	28.69	39.41	37.33	28.62	38.87	ND
	17	26.97	N.D	N.D	27.72	36.58	35.63	27.66	37.4	36.84	28.41	38.01	36.46	28.84	38.35	37.13	28.49	38.77	37.3	28.59	ND	38.68
	18	27.27	N.D	N.D	27.77	37.61	35.36	27.98	37.07	36.45	27.91	38.79	36.75	28.98	38.4	37.25	28.6	40.25	37.15	28.78	ND	ND
	19	27.12	N.D	N.D	28.13	35.97	35.73	27.51	36.92	37.15	28.09	38.2	36.24	28.63	38.85	37.61	28.92	38.36	37.48	28.34	39.19	38.96
	20	28.08	N.D	N.D	29.05	37.03	36.31	27.78	37.44	36.43	28.62	38.76	37.12	28.69	38.7	37.1	29.97	41.24	37.88	28.59	ND	ND

Lot	Matrix	Swab																				
	Repl	Neg			240 copies/㎖			200 copies/㎖			160 copies/㎖			120 copies/㎖			80 copies/㎖			40 copies/㎖		
		IPC	Pan-Sarbecovirus gene	SARS-CoV-2	IPC	Pan-Sarbecovirus gene	SARS-CoV-2	IPC	Pan-Sarbecovirus gene	SARS-CoV-2	IPC	Pan-Sarbecovirus gene	SARS-CoV-2	IPC	Pan-Sarbecovirus gene	SARS-CoV-2	IPC	Pan-Sarbecovirus gene	SARS-CoV-2	IPC	Pan-Sarbecovirus gene	SARS-CoV-2
200111H	1	25.4	N.D	N.D	28.56	37.26	35.63	28.58	37.83	36.19	29.34	38.26	36.93	28.3	37.55	36.49	28.98	38.64	37.52	28.78	40.54	38.57
	2	25.23	N.D	N.D	28.12	36.94	37.52	29.09	37.37	36.58	28.84	37	36.95	33.63	42.3	37.75	32	41.9	38.21	29.07	39.44	39.32

	**3**	24.73	N.D	N.D	28.05	36.75	35.77	28.51	37.6	36.53	28.89	37.17	37.54	28.66	38.13	37.1	28.72	38.99	37.5	27.96	40.95	39.2
	**4**	25.05	N.D	N.D	29.19	37.42	36.05	28.76	37.51	36.55	28.17	37.16	36.61	30.2	38.24	36.89	29.01	38.83	37.74	28.25	38.18	39
	**5**	27.07	N.D	N.D	29.26	37.14	35.86	29.31	37.68	36.37	28.73	36.77	37.05	29.22	37.57	36.73	28.46	37.73	37.18	29.18	ND	39.25
	**6**	27.05	N.D	N.D	29.5	36.77	37.77	29.68	37.24	36.79	29.35	38.4	37.71	29.05	37.95	36.84	29.27	ND	ND	28.97	40.32	38.86
	**7**	27.06	N.D	N.D	28.69	36.59	36.03	29.21	37.44	36.74	29.32	38.03	37.74	29.2	38.05	36.68	29.19	38.35	37.63	28.58	39.66	38.25
	**8**	27.04	N.D	N.D	29.96	37.27	36.25	29.42	37.37	36.76	28.93	37.84	36.97	29.26	37.37	36.88	29.37	38.87	37.79	29.39	36.1	39.17
	**9**	27.13	N.D	N.D	30.01	37.49	36.59	29.03	37.03	36.19	29.59	36.27	37.81	29.47	38.87	37.12	29.39	39.48	38.12	29.78	ND	39.53
	**10**	26.98	N.D	N.D	29.39	36.41	36.6	29.87	37.27	37.42	29.15	38.04	37.48	29.9	ND	ND	29.88	40.45	38.24	29.43	43.03	39.78
	**11**	27.09	N.D	N.D	29.45	36.67	37.22	29.29	37.44	36.79	29.25	38.54	37.57	29.48	38.36	37.49	29.9	40.79	38.26	29.46	41.16	39.95
	**12**	26.34	N.D	N.D	28.81	36.61	36.29	30.64	37.58	36.59	29.32	39.13	37.62	29.52	37.86	37.15	29.96	40.03	38.05	29.75	41.23	39.1
	**13**	27.03	N.D	N.D	28.44	36.91	36.45	29.07	37.17	36.93	29.59	38.5	37.11	29.06	38.41	36.6	28.74	38.8	37.63	29.38	39.44	38.12
	**14**	26.98	N.D	N.D	28.48	36.81	36.15	28.15	37.45	36.24	28.74	38.31	36.36	28.6	36	36.71	28.8	38.77	37.31	28.43	39.52	ND
	**15**	27.08	N.D	N.D	27.96	37.12	35.79	27.93	37.67	36.78	28.46	37.5	36.73	28.65	37.95	36.86	28.23	39.28	ND	28.26	39.72	37.94
	**16**	27.33	N.D	N.D	28.85	37.17	35.98	27.99	37.47	36.29	29.15	38.53	37.01	28.91	38.31	37.21	29.12	39.18	37.07	28.28	40.08	ND
	**17**	26.54	N.D	N.D	28.66	36.91	36.29	28.41	37	36.6	28.7	38.22	36.35	29.18	38.7	37.05	29.35	39.16	37.71	29.17	39.13	38.27
	**18**	27.1	N.D	N.D	28.52	37.1	35.95	27.53	38.28	36.69	28.68	38	36.65	29.02	38.74	37.19	29.23	39.39	37.56	28.42	40.68	38.32
	**19**	26.25	N.D	N.D	28.58	35.57	36.23	27.99	36.97	36.91	29.01	37.6	37.38	29.09	38.85	37.34	29.29	ND	37.7	28.76	38.98	38.28
	**20**	27.3	N.D	N.D	28.36	37.16	36.26	28.31	36.94	36.6	29.29	38.42	36.78	28.66	38.03	37.1	29.12	39.65	37.68	29.07	39.05	38.19
**Lot**	**Matrix**	**Saliva**																				
	**Repl**	**Neg**			**240 copies/㎖**			**200 copies/㎖**			**160 copies/㎖**			**120 copies/㎖**			**80 copies/㎖**			**40 copies/㎖**		
		**IPC**	**E gene**	**SARS-CoV-2**	**IPC**	**E gene**	**SARS-CoV-2**	**IPC**	**E gene**	**SARS-CoV-2**	**IPC**	**E gene**	**SARS-CoV-2**	**IPC**	**E gene**	**SARS-CoV-2**	**IPC**	**E gene**	**SARS-CoV-2**	**IPC**	**E gene**	**SARS-CoV-2**
**200111H**	**1**	Valid	N.D	N.D	Valid	37.14	35.59	Valid	37.09	36.15	Valid	37.52	36.15	Valid	37.78	37.14	Valid	40.02	37.26	Valid	39.32	N.D
	**2**	Valid	N.D	N.D	Valid	37.14	35.77	Valid	37.93	36.28	Valid	38.18	36.67	Valid	38.34	37.27	Valid	37.53	36.46	Valid	N.D	37.98
	**3**	Valid	N.D	N.D	Valid	36.56	35.45	Valid	35.62	35.78	Valid	37.37	36.07	Valid	39.39	36.97	Valid	39.22	37.02	Valid	40.75	37.79
	**4**	Valid	N.D	N.D	Valid	36.44	35.19	Valid	35.65	36.42	Valid	37.34	36.58	Valid	35.87	36.99	Valid	38.75	37.37	Valid	N.D	39.53
	**5**	Valid	N.D	N.D	Valid	36.85	36.06	Valid	36.55	35.83	Valid	38.15	36.37	Valid	37.52	36.67	Valid	39.54	37.13	Valid	39.24	38.70
	**6**	Valid	N.D	N.D	Valid	37.02	35.59	Valid	37.59	35.86	Valid	37.72	36.59	Valid	37.99	N.D	Valid	39.45	37.63	Valid	41.02	38.69
	**7**	Valid	N.D	N.D	Valid	37.15	35.45	Valid	37.11	35.71	Valid	37.47	35.93	Valid	39.34	36.88	Valid	37.77	N.D	Valid	40.04	38.17
	**8**	Valid	N.D	N.D	Valid	36.18	35.57	Valid	37.16	36.08	Valid	37.30	37.26	Valid	40.54	37.57	Valid	39.20	36.77	Valid	38.86	39.05
	**9**	Valid	N.D	N.D	Valid	36.62	35.78	Valid	37.90	36.34	Valid	37.67	36.40	Valid	38.38	37.60	Valid	38.45	38.26	Valid	38.66	38.31
	**10**	Valid	N.D	N.D	Valid	36.52	35.73	Valid	37.42	36.13	Valid	38.67	36.41	Valid	38.07	37.28	Valid	37.96	38.18	Valid	40.07	38.42
	**11**	Valid	N.D	N.D	Valid	36.43	36.07	Valid	37.69	36.46	Valid	37.78	36.71	Valid	37.77	37.66	Valid	38.69	37.47	Valid	39.49	39.34
	**12**	Valid	N.D	N.D	Valid	36.77	35.36	Valid	37.08	36.68	Valid	37.31	36.67	Valid	38.58	37.11	Valid	38.77	37.22	Valid	39.69	38.90
	**13**	Valid	N.D	N.D	Valid	37.01	36.57	Valid	36.66	36.26	Valid	37.11	36.12	Valid	37.57	36.79	Valid	39.13	38.03	Valid	39.74	39.04

	14	Valid	N.D	N.D	Valid	36.37	35.75	Valid	37.19	35.71	Valid	37.50	36.33	Valid	37.92	36.62	Valid	39.39	38.22	Valid	41.27	38.10
	15	Valid	N.D	N.D	Valid	37.11	36.43	Valid	36.57	36.33	Valid	37.57	36.22	Valid	37.33	36.73	Valid	38.16	N.D	Valid	38.62	39.37
	16	Valid	N.D	N.D	Valid	36.49	36.13	Valid	36.86	36.18	Valid	36.77	36.74	Valid	37.06	36.32	Valid	39.68	37.84	Valid	41.03	39.77
	17	Valid	N.D	N.D	Valid	37.05	35.81	Valid	37.94	36.08	Valid	38.27	36.80	Valid	38.18	36.21	Valid	37.48	36.59	Valid	39.83	39.29
	18	Valid	N.D	N.D	Valid	36.63	35.46	Valid	37.16	35.93	Valid	37.71	36.01	Valid	37.76	36.63	Valid	38.91	38.30	Valid	39.50	37.70
	19	Valid	42.06	N.D	Valid	36.89	35.38	Valid	37.84	36.06	Valid	38.32	35.71	Valid	37.20	37.49	Valid	39.49	37.68	Valid	38.89	37.53
	20	Valid	N.D	N.D	Valid	36.71	35.47	Valid	37.26	36.23	Valid	38.06	36.41	Valid	37.37	36.99	Valid	38.94	36.91	Valid	39.62	N.D

2 200211H Lot

Lot	Matrix	Sputum																				
	Repl	Neg			240 copies/㎖			200 copies/㎖			160 copies/㎖			120 copies/㎖			80 copies/㎖			40 copies/㎖		
		IPC	Pan-Sarbecovirus gene	SARS-CoV-2	IPC	Pan-Sarbecovirus gene	SARS-CoV-2	IPC	Pan-Sarbecovirus gene	SARS-CoV-2	IPC	Pan-Sarbecovirus gene	SARS-CoV-2	IPC	Pan-Sarbecovirus gene	SARS-CoV-2	IPC	Pan-Sarbecovirus gene	SARS-CoV-2	IPC	Pan-Sarbecovirus gene	SARS-CoV-2
200211H	1	24.93	N.D	N.D	24.7	38.49	36.64	24.37	37.32	35.91	24.15	38.09	36.32	25.06	ND	37.39	25.1	39.21	38.54	25.32	40.87	39.95
	2	24.56	N.D	N.D	25.02	37.25	36.2	25.08	36.72	36.86	24.16	36.83	36.42	25.7	40.18	37.47	24.55	40.11	37.36	25.58	ND	37.52
	3	24.29	N.D	N.D	24.51	37.07	35.8	24.81	36.6	37.37	24.33	37.61	36.58	25.93	39.09	38.28	24.57	41.4	36.88	24.78	ND	38.44
	4	31.21	N.D	N.D	25.14	38.36	36.03	23.69	37.09	36.19	25.1	39.34	36.45	24.4	38.21	36.79	25.67	39.2	37.21	25.25	39.86	ND
	5	25.56	N.D	N.D	25.72	37.64	35.44	25.51	38.19	36.82	25.07	37.01	36.21	25.53	38.2	36.75	26.23	38.59	36.81	25.38	43.2	37.65
	6	25.5	N.D	N.D	25.57	36.82	35.86	25.73	37.37	36.26	25.69	37.04	36.25	24.97	38.4	36.69	26.63	40.88	37.11	26.65	40.56	39.1
	7	25.26	N.D	N.D	25.99	36.43	35.21	25.67	37.76	36.21	25.5	37.29	36.5	26.04	38.1	36.52	25.43	38.81	36.84	24.91	ND	38.01
	8	25.2	N.D	N.D	25.07	37.41	35.98	26.41	39.22	36.17	25.15	36.93	36.13	26.4	40.42	37.08	26.13	ND	37.46	25.07	40.03	38.01
	9	25.59	N.D	N.D	26.01	37.54	37.02	25.45	36.49	36.21	25.27	37.26	36.65	26.08	38.79	37.74	26.2	38.29	38.94	26.46	38.56	40.48
	10	25.78	N.D	N.D	26.22	36.39	36.54	26.17	36.05	37.18	25.24	36.12	36.77	26.89	38.14	37.85	25.63	39.02	37.71	26.64	41.05	37.94
	11	25.09	N.D	N.D	25.69	36.21	36.16	26.07	35.92	37.73	25.52	36.62	36.96	27.2	38.13	38.61	25.8	39.59	37.19	25.88	ND	38.84
	12	32.51	N.D	N.D	26.17	37.26	36.31	24.86	36.26	36.5	26.15	38.06	36.83	25.67	37.3	37.12	26.73	37.94	37.52	26.61	38.9	37.59
	13	26.16	N.D	N.D	25.32	35.93	36.05	25.5	36.79	36.65	25.47	37.38	36.25	25.33	38.15	36.02	25.9	37.79	36.57	25.48	38.22	ND
	14	25.66	N.D	N.D	25.4	37.09	36.25	25.77	36.37	36.13	25.46	37.42	37.1	25.79	37.64	37.27	25.61	37	37.05	25.88	39.33	ND
	15	25.85	N.D	N.D	25.29	36.44	36.12	26.25	38.1	36.17	25.19	37.83	36.72	26.4	36.94	37.4	25.54	39.39	36.86	26.55	39.38	38.41
	16	25.23	N.D	N.D	25.19	36.77	36.04	25.73	38.01	35.83	25.58	37.12	36.47	26.13	37.45	37.08	25.36	39.69	37.61	26.13	39.49	37.3
	17	26.47	N.D	N.D	25.84	36.5	35.53	26.12	37.4	36.2	26	38.09	35.8	25.92	38.77	35.48	26.23	38.5	ND	26.08	39.04	ND
	18	26.06	N.D	N.D	25.98	37.72	35.81	26.31	37.01	35.65	26.03	38.18	36.61	26.29	38.27	36.86	26.08	37.65	ND	26.36	40.08	ND
	19	26.22	N.D	N.D	25.83	37.07	35.62	26.72	38.77	35.72	25.67	38.5	36.25	26.77	37.58	36.96	26.03	40.76	36.33	26.97	40.26	37.94

	**20**	25.61	N.D	N.D	25.7	37.37	35.54	26.2	38.73	35.33	26.15	37.77	36.08	26.45	38.24	36.55	25.86	ND	37.17	26.41	40.3	ND
**Lot**	**Matrix**	**Swab**																				
	**Repl**	**Neg**			**240 copies/㎖**			**200 copies/㎖**			**160 copies/㎖**			**120 copies/㎖**			**80 copies/㎖**			**40 copies/㎖**		
		**IPC**	**Pan-Sarbecovirus gene**	**SARS-CoV-2**	**IPC**	**Pan-Sarbecovirus gene**	**SARS-CoV-2**	**IPC**	**Pan-Sarbecovirus gene**	**SARS-CoV-2**	**IPC**	**Pan-Sarbecovirus gene**	**SARS-CoV-2**	**IPC**	**Pan-Sarbecovirus gene**	**SARS-CoV-2**	**IPC**	**Pan-Sarbecovirus gene**	**SARS-CoV-2**	**IPC**	**Pan-Sarbecovirus gene**	**SARS-CoV-2**
**200211H**	**1**	24.18	N.D	N.D	25.23	37.09	36.38	23.77	37.65	36.59	22.95	37.85	36.96	24.54	38.27	36.2	24.33	39.94	36.32	23.4	38.98	39.62
	**2**	24.34	N.D	N.D	24.17	38.27	36.3	24.51	37.68	37.56	23.51	37.6	36.3	24.54	37.88	37.7	25.1	39.31	37.25	24.77	ND	37.32
	**3**	25.02	N.D	N.D	23.97	37.55	35.79	25.43	37.74	36.97	23.21	38.45	36.26	26.21	39.98	36.98	24.58	38.76	37.39	25.62	39	ND
	**4**	24.09	N.D	N.D	24.24	37.96	36.17	26.35	38.1	37.08	24.15	37.36	37.08	25.11	37.66	37.15	23.59	38.04	36.52	24.66	42.27	37.63
	**5**	24.73	N.D	N.D	26	37.01	36.2	24.65	37.53	36.38	23.62	37.73	36.72	25.33	38.17	36.05	25.1	39.7	ND	24.27	38.81	39.43
	**6**	25	N.D	N.D	25.03	38.18	36.15	25.33	37.57	37.35	24.27	37.5	36.13	25.32	37.75	37.49	25.83	39.22	37.07	25.52	ND	37.14
	**7**	25.51	N.D	N.D	24.76	37.45	35.6	26.22	37.65	36.76	23.95	38.35	36.11	27	39.82	36.73	25.33	38.63	37.2	26.25	39.12	ND
	**8**	24.74	N.D	N.D	25.11	37.84	36.03	27.09	37.98	36.88	25.01	37.24	36.9	25.76	37.55	37	24.41	37.91	36.33	25.28	43.13	37.41
	**9**	25.96	N.D	N.D	26.17	37.01	36.03	26.05	37.36	37.25	25.48	36.03	36.83	26	37.25	37.26	26.68	37.55	37.33	25.79	41.42	38.19
	**10**	25.88	N.D	N.D	26.1	36.13	36.33	26.14	36.64	36.82	26.16	36.28	36.77	25.29	37.48	37.23	27.11	39.55	37.63	27.13	39.15	39.64
	**11**	25.57	N.D	N.D	26.43	35.82	35.72	26.21	37.04	36.72	25.92	36.56	37.06	26.54	37.23	37.08	26.05	37.66	37.38	25.38	ND	38.51
	**12**	25.49	N.D	N.D	25.45	36.72	36.43	26.95	38.06	36.7	25.49	36.21	36.61	26.96	39.42	37.57	26.64	39.73	38.06	25.38	38.89	38.52
	**13**	25.33	N.D	N.D	25.84	36.98	36.5	26.26	37.36	36.78	25.58	37.19	36.61	25.92	39	38.34	25.3	38.02	36.53	25.38	42.19	37.82
	**14**	24.91	N.D	N.D	25.91	37.08	36.08	26.3	37.16	35.73	25.74	36.67	36.68	26.09	38.35	36.28	25.92	36.95	37.21	26.11	ND	39.26
	**15**	25.39	N.D	N.D	25.78	37.09	36.27	25.65	36.19	36.61	25.31	36.69	36.07	25.52	37.09	37.06	25.34	ND	36.62	25.68	40.19	38.07
	**16**	25.32	N.D	N.D	25.86	36.51	36.08	25.65	37.05	37.59	26.05	37.06	36.27	25.49	37.18	37.09	25.75	40.06	38.5	25.79	38.8	37.17
	**17**	25.8	N.D	N.D	26.29	37.58	36.08	26.82	38.14	36.28	26.1	37.89	36.18	26.32	39.82	37.86	25.78	38.65	36.11	25.98	43.17	37.33
	**18**	25.3	N.D	N.D	26.32	37.75	35.58	26.86	37.87	35.28	26.22	37.32	36.21	26.53	39.29	35.85	26.46	37.65	36.75	26.59	ND	38.74
	**19**	25.88	N.D	N.D	26.26	37.84	35.87	26.22	36.8	36.15	25.8	37.33	35.55	26.09	37.77	36.53	25.84	ND	ND	26.18	40.99	37.51
	**20**	25.81	N.D	N.D	26.26	37.2	35.58	26.16	37.71	37.14	26.45	37.65	35.83	25.99	37.88	36.58	26.22	40.73	38.08	26.23	39.88	ND
**Lot**	**Matrix**	**Saliva**																				
	**Repl**	**Neg**			**240 copies/㎖**			**200 copies/㎖**			**160 copies/㎖**			**120 copies/㎖**			**80 copies/㎖**			**40 copies/㎖**		
		**IPC**	**E gene**	**SARS-CoV-2**	**IPC**	**E gene**	**SARS-CoV-2**	**IPC**	**E gene**	**SARS-CoV-2**	**IPC**	**E gene**	**SARS-CoV-2**	**IPC**	**E gene**	**SARS-CoV-2**	**IPC**	**E gene**	**SARS-CoV-2**	**IPC**	**E gene**	**SARS-CoV-2**
**200211H**	**1**	Valid	N.D	N.D	Valid	36.95	36.05	Valid	36.88	35.52	Valid	37.66	37.33	Valid	37.44	36.56	Valid	39.39	38.08	Valid	38.12	37.49
	**2**	Valid	N.D	N.D	Valid	36.47	35.76	Valid	37.11	35.94	Valid	37.52	36.56	Valid	37.71	37.15	Valid	38.81	38.03	Valid	39.05	38.94
	**3**	Valid	N.D	N.D	Valid	36.50	35.62	Valid	36.90	36.34	Valid	37.32	36.70	Valid	37.61	36.65	Valid	38.28	37.14	Valid	39.67	N.D
	**4**	Valid	N.D	N.D	Valid	37.02	35.48	Valid	37.28	36.48	Valid	36.59	36.46	Valid	37.66	37.14	Valid	38.22	38.17	Valid	38.46	38.10
	**5**	Valid	N.D	N.D	Valid	36.85	36.15	Valid	36.44	34.29	Valid	37.24	35.48	Valid	37.96	36.82	Valid	39.17	37.28	Valid	38.68	40.40

	**6**	Valid	N.D	N.D	Valid	37.35	36.40	Valid	36.31	34.24	Valid	37.23	35.77	Valid	37.82	36.75	Valid	38.69	36.92	Valid	37.72	39.17
	**7**	Valid	N.D	N.D	Valid	36.48	36.16	Valid	37.42	34.70	Valid	36.75	36.01	Valid	38.06	36.96	Valid	38.98	38.04	Valid	N.D	38.34
	**8**	Valid	N.D	N.D	Valid	36.62	35.97	Valid	36.48	34.66	Valid	36.02	35.56	Valid	38.94	36.28	Valid	37.79	36.28	Valid	39.31	38.41
	**9**	Valid	N.D	N.D	Valid	36.91	36.09	Valid	36.56	33.69	Valid	37.07	36.53	Valid	37.13	36.25	Valid	40.21	39.12	Valid	38.95	38.52
	**10**	Valid	N.D	N.D	Valid	36.34	35.98	Valid	36.00	33.81	Valid	37.18	36.45	Valid	38.12	36.60	Valid	39.55	39.03	Valid	38.70	38.71
	**11**	Valid	N.D	N.D	Valid	36.42	36.22	Valid	36.32	34.23	Valid	36.53	36.50	Valid	39.32	36.98	Valid	38.39	38.05	Valid	37.92	37.93
	**12**	Valid	N.D	N.D	Valid	37.17	35.92	Valid	36.51	34.12	Valid	37.34	36.02	Valid	38.28	36.45	Valid	38.86	37.29	Valid	38.46	38.11
	**13**	Valid	N.D	N.D	Valid	36.61	35.81	Valid	36.33	33.54	Valid	37.04	36.39	Valid	37.09	35.34	Valid	38.10	38.24	Valid	37.23	39.87
	**14**	Valid	N.D	N.D	Valid	36.95	35.78	Valid	36.05	33.52	Valid	36.94	35.90	Valid	N.D	N.D	Valid	38.12	38.25	Valid	37.36	38.62
	**15**	Valid	N.D	N.D	Valid	36.77	36.54	Valid	36.31	34.78	Valid	36.66	35.70	Valid	42.40	36.64	Valid	39.32	38.08	Valid	37.36	N.D
	**16**	Valid	N.D	N.D	Valid	37.01	35.90	Valid	36.40	34.72	Valid	36.47	36.34	Valid	37.57	37.02	Valid	38.95	37.46	Valid	N.D	39.66
	**17**	Valid	N.D	N.D	Valid	36.72	35.08	Valid	37.71	36.07	Valid	37.52	36.34	Valid	37.87	36.20	Valid	N.D	38.09	Valid	N.D	38.93
	**18**	Valid	N.D	N.D	Valid	35.94	35.23	Valid	37.88	37.19	Valid	38.17	36.88	Valid	37.71	36.87	Valid	N.D	38.07	Valid	37.52	38.45
	**19**	Valid	N.D	N.D	Valid	37.20	36.06	Valid	37.20	36.06	Valid	37.51	35.95	Valid	38.09	36.16	Valid	40.36	37.83	Valid	38.47	38.28
	**20**	Valid	N.D	N.D	Valid	37.24	35.24	Valid	37.24	36.24	Valid	37.99	36.78	Valid	37.61	36.44	Valid	40.07	N.D	Valid	37.37	N.D

2. *AccuPower*® SARS-CoV-2 Multiplex Real-Time RT-PCR Kit (SCVM)

LoD- Hit Rate Analysis

2.1 *ExiPrep*™48 Dx/*Exicycler*™96 V4

Matrix	Lot	Nominal Concentration (copies/㎕)	Number of replicates tested (N)	Pan-Sarbecovirus gene		SARS-CoV-2 gene	
				Number of positives detected (N)	Positive rate (%)	Number of positives detected (N)	Positive rate (%)
Sputum	All 3 Lots combined	6	60	60	100%	60	100%
		5	60	60	100%	60	100%
		4	60	60	100%	60	100%
		3	60	59	98%	60	100%
		2	60	58	97%	57	95%
		1	60	54	90%	52	87%
		0	60	0	0%	0	0%
	2001G	6	20	20	100%	20	100%
		5	20	20	100%	20	100%
		4	20	20	100%	20	100%
		3	20	20	100%	20	100%
		2	20	19	95%	19	95%
		1	20	17	85%	17	85%
		0	20	0	0%	0	0%
	2002G	6	20	20	100%	20	100%
		5	20	20	100%	20	100%
		4	20	20	100%	20	100%
		3	20	20	100%	20	100%
		2	20	19	95%	19	95%
		1	20	19	95%	18	90%
		0	20	0	0%	0	0%
	2003G	6	20	20	100%	20	100%
		5	20	20	100%	20	100%
		4	20	20	100%	20	100%
		3	20	19	95%	20	100%
		2	20	20	100%	19	95%
		1	20	18	90%	17	85%
		0	20	0	0%	0	0%
Swab	All 3 Lots combined	6	60	60	100%	60	100%
		5	60	60	100%	60	100%
		4	60	60	100%	60	100%

		3	60	58	97%	59	98%
		2	60	57	95%	57	95%
		1	60	55	92%	54	90%
		0	60	0	0%	0	0%
	**2001G**	6	20	20	100%	20	100%
		5	20	20	100%	20	100%
		4	20	20	100%	20	100%
		3	20	19	95%	20	100%
		2	20	19	95%	20	100%
		1	20	18	90%	19	95%
		0	20	0	0%	0	0%
	**2002G**	6	20	20	100%	20	100%
		5	20	20	100%	20	100%
		4	20	20	100%	20	100%
		3	20	19	95%	20	100%
		2	20	19	95%	19	95%
		1	20	19	95%	17	85%
		0	20	0	0%	0	0%
	**2003G**	6	20	20	100%	20	100%
		5	20	20	100%	20	100%
		4	20	20	100%	20	100%
		3	20	20	100%	19	95%
		2	20	19	95%	18	90%
		1	20	18	90%	18	90%
		0	20	0	0%	0	0%
**Saliva**	**All 3 Lots combined**	6	60	60	100%	60	100%
		5	60	60	100%	60	100%
		4	60	60	100%	60	100%
		3	60	60	100%	59	98%
		2	60	58	97%	57	95%
		1	60	53	88%	51	85%
		0	60	1	2%	2	3%
	**2101D**	6	20	20	100%	20	100%
		5	20	20	100%	20	100%
		4	20	20	100%	20	100%
		3	20	20	100%	19	95%
		2	20	19	95%	19	95%

		1	20	17	85%	17	85%
		0	20	0	0%	1	5%
	2102D	6	20	20	100%	20	100%
		5	20	20	100%	20	100%
		4	20	20	100%	20	100%
		3	20	20	100%	20	100%
		2	20	20	100%	19	95%
		1	20	18	90%	17	85%
		0	20	1	5%	0	0%
	2103D	6	20	20	100%	20	100%
		5	20	20	100%	20	100%
		4	20	20	100%	20	100%
		3	20	20	100%	20	100%
		2	20	19	95%	19	95%
		1	20	18	90%	17	85%
		0	20	0	0%	1	5%

[Limit of Detection of the *AccuPower*® SARS-CoV-2 Multiplex Real-Time RT-PCR Kit]

Matrix	Instrument	Pan-Sarbecovirus gene	SARS-CoV-2 gene
Sputum	• *Exicycler*™96 V4	2 copies/㎕	2 copies/㎕
Swab	• *Exicycler*™96 V4	2 copies/㎕	2 copies/㎕
Saliva	• *Exicycler*™96 V4	2 copies/㎕	2 copies/㎕

2.2 *ExiPrep*™48 Dx/ CFX96™ Dx Real-Time PCR Detection System

Matrix	Lot	Nominal Concentration (copies/㎕)	Number of replicates tested (N)	Pan-Sarbecovirus gene		SARS-CoV-2 gene	
				Number of positives detected (N)	Positive rate (%)	Number of positives detected (N)	Positive rate (%)
Sputum	All 3 Lots combined	6	60	60	100%	60	100%
		5	60	60	100%	60	100%
		4	60	60	100%	60	100%
		3	60	60	100%	60	100%
		2	60	58	97%	58	97%
		1	60	55	92%	55	92%
		0	60	0	0%	0	0%
	2001G	6	20	20	100%	20	100%
		5	20	20	100%	20	100%
		4	20	20	100%	20	100%

		3	20	20	100%	20	100%
		2	20	19	95%	19	95%
		1	20	19	95%	19	95%
		0	20	0	0%	0	0%
	**2002G**	6	20	20	100%	20	100%
		5	20	20	100%	20	100%
		4	20	20	100%	20	100%
		3	20	20	100%	20	100%
		2	20	19	95%	19	95%
		1	20	17	85%	17	85%
		0	20	0	0%	0	0%
	**2003G**	6	20	20	100%	20	100%
		5	20	20	100%	20	100%
		4	20	20	100%	20	100%
		3	20	20	100%	20	100%
		2	20	20	100%	20	100%
		1	20	19	95%	19	95%
		0	20	0	0%	0	0%
**Swab**	**All 3 Lots combined**	6	60	60	100%	60	100%
		5	60	60	100%	60	100%
		4	60	60	100%	60	100%
		3	60	60	100%	60	100%
		2	60	58	97%	58	97%
		1	60	55	92%	55	92%
		0	60	0	0%	0	0%
	**2001G**	6	20	20	100%	20	100%
		5	20	20	100%	20	100%
		4	20	20	100%	20	100%
		3	20	20	100%	20	100%
		2	20	19	95%	19	95%
		1	20	19	95%	19	95%
		0	20	0	0%	0	0%
	**2002G**	6	20	20	100%	20	100%
		5	20	20	100%	20	100%
		4	20	20	100%	20	100%
		3	20	20	100%	20	100%
		2	20	19	95%	19	95%
		1	20	19	95%	19	95%
		0	20	0	0%	0	0%
	**2003G**	6	20	20	100%	20	100%

		5	20	20	100%	20	100%
		4	20	20	100%	20	100%
		3	20	20	100%	20	100%
		2	20	20	100%	20	100%
		1	20	17	85%	17	85%
		0	20	0	0%	0	0%
**Saliva**	**All 3 Lots combined**	6	60	60	100%	60	100%
		5	60	60	100%	60	100%
		4	60	60	100%	60	100%
		3	60	60	100%	60	100%
		2	60	59	98%	60	100%
		1	60	53	88%	55	92%
		0	60	1	2%	1	2%
	**2101D**	6	20	20	100%	20	100%
		5	20	20	100%	20	100%
		4	20	20	100%	20	100%
		3	20	20	100%	20	100%
		2	20	19	95%	20	100%
		1	20	18	90%	20	100%
		0	20	1	5%	0	0%
	**2102D**	6	20	20	100%	20	100%
		5	20	20	100%	20	100%
		4	20	20	100%	20	100%
		3	20	20	100%	20	100%
		2	20	20	100%	20	100%
		1	20	19	95%	19	95%
		0	20	0	0%	1	5%
	**2103D**	6	20	20	100%	20	100%
		5	20	20	100%	20	100%
		4	20	20	100%	20	100%
		3	20	20	100%	20	100%
		2	20	20	100%	20	100%
		1	20	16	80%	16	80%
		0	20	0	0%	0	0%

[Limit of Detection of the *AccuPower®* SARS-CoV-2 Multiplex Real-Time RT-PCR Kit]

Matrix	Instrument	Pan-Sarbecovirus gene	SARS-CoV-2 gene
Sputum	CFX96™ Dx System	2 copies/㎕	2 copies/㎕
Swab	CFX96™ Dx System	2 copies/㎕	2 copies/㎕
Saliva	CFX96™ Dx System	2 copies/㎕	2 copies/㎕

Result of Ct value in LoD study

- *ExiPrep™*48 Dx/*Exicycler™*96 V4

Lot	Day	Matrix	Sputum																				
			Neg			6 copies/㎕			5 copies/㎕			4 copies/㎕			3 copies/㎕			2 copies/㎕			1 copies/㎕		
		Repl	IPC	Pan-Sarbecovirus gene	SARS-CoV-2 gene	IPC	Pan-Sarbecovirus gene	SARS-CoV-2 gene	IPC	Pan-Sarbecovirus gene	SARS-CoV-2 gene	IPC	Pan-Sarbecovirus gene	SARS-CoV-2 gene	IPC	Pan-Sarbecovirus gene	SARS-CoV-2 gene	IPC	Pan-Sarbecovirus gene	SARS-CoV-2 gene	IPC	Pan-Sarbecovirus gene	SARS-CoV-2 gene
2001G	1	1	Valid	N.D	N.D	Valid	30.35	30.68	Valid	31.18	30.87	Valid	30.79	30.72	Valid	31.13	31.48	Valid	32.31	N.D	Valid	33.32	N.D
		2	Valid	N.D	N.D	Valid	30.59	30.82	Valid	31.75	31.31	Valid	30.88	30.36	Valid	31.00	30.95	Valid	31.87	32.08	Valid	N.D	33.28
		3	Valid	N.D	N.D	Valid	30.28	30.06	Valid	30.04	30.40	Valid	31.12	30.84	Valid	31.07	31.29	Valid	32.19	32.14	Valid	33.7	33.69
		4	Valid	N.D	34.15	Valid	30.31	30.44	Valid	30.74	30.46	Valid	31.41	30.90	Valid	31.82	31.43	Valid	32.14	32.38	Valid	32.61	32.64
	2	5	Valid	N.D	N.D	Valid	31.21	30.99	Valid	31.41	31.59	Valid	31.29	31.19	Valid	32.13	32.40	Valid	32.18	32.23	Valid	N.D	33.33
		6	Valid	N.D	N.D	Valid	30.76	31.02	Valid	31.10	31.10	Valid	31.75	31.82	Valid	31.84	32.33	Valid	33.12	32.25	Valid	33.46	34.13
		7	Valid	N.D	N.D	Valid	30.67	31.05	Valid	31.16	30.85	Valid	31.30	31.47	Valid	32.11	32.19	Valid	32.38	33.01	Valid	33.55	N.D
		8	Valid	N.D	N.D	Valid	30.92	31.22	Valid	30.73	31.03	Valid	31.82	31.41	Valid	32.11	32.38	Valid	32.45	32.38	Valid	33.77	33.07
	3	9	Valid	N.D	N.D	Valid	30.68	31.05	Valid	31.48	31.18	Valid	31.12	31.07	Valid	31.43	31.83	Valid	32.67	32.17	Valid	33.74	33.32
		10	Valid	N.D	N.D	Valid	30.96	31.15	Valid	32.10	31.64	Valid	31.19	30.69	Valid	31.30	31.23	Valid	32.20	32.35	Valid	33.67	33.62
		11	Valid	N.D	N.D	Valid	30.57	30.34	Valid	30.31	30.74	Valid	31.41	31.14	Valid	31.36	31.62	Valid	32.53	32.44	Valid	34.10	34.07
		12	Valid	N.D	N.D	Valid	30.63	30.78	Valid	31.08	30.82	Valid	31.77	31.21	Valid	32.19	31.78	Valid	32.45	32.74	Valid	33.02	33.01
	4	13	Valid	N.D	N.D	Valid	30.80	30.85	Valid	31.2	31.43	Valid	31.13	31.22	Valid	31.36	31.23	Valid	32.38	33.32	Valid	33.97	N.D
		14	Valid	35.02	N.D	Valid	30.77	30.54	Valid	31.16	31.01	Valid	31.2	31.05	Valid	32.06	31.79	Valid	33.08	33.15	Valid	33.36	33.25
		15	Valid	36.34	N.D	Valid	30.42	30.76	Valid	31.16	30.76	Valid	31.21	30.62	Valid	30.87	31.14	Valid	33.46	33.57	Valid	32.35	32.01
		16	Valid	N.D	N.D	Valid	30.56	31.09	Valid	31.31	31.13	Valid	31.71	31.86	Valid	32.02	31.28	Valid	33.92	33.24	Valid	33.00	32.41
	5	17	Valid	N.D	N.D	Valid	30.41	30.75	Valid	31.32	31.24	Valid	30.54	31.14	Valid	31.18	31.5	Valid	32.04	32.15	Valid	32.94	34.06
		18	Valid	N.D	34.97	Valid	30.55	31.28	Valid	30.29	31.07	Valid	31.28	31.03	Valid	31.23	31.72	Valid	31.76	32.33	Valid	32.45	33.08
		19	Valid	N.D	N.D	Valid	30.50	30.97	Valid	30.54	30.39	Valid	31.05	31.35	Valid	31.67	31.29	Valid	N.D	32.72	Valid	32.87	32.60
		20	Valid	N.D	N.D	Valid	30.47	30.55	Valid	30.80	31.40	Valid	31.30	31.40	Valid	31.82	32.40	Valid	32.37	32.50	Valid	N.D	32.98
2002G	1	1	Valid	N.D	N.D	Valid	30.49	30.44	Valid	30.68	30.72	Valid	30.66	30.83	Valid	31.22	31.21	Valid	32.19	32.07	Valid	33.28	33.29
		2	Valid	N.D	N.D	Valid	30.70	30.27	Valid	31.41	31.02	Valid	31.29	31.03	Valid	31.66	31.15	Valid	32.21	31.89	Valid	33.74	33.33

		**3**	Valid	37.65	N.D	Valid	30.73	30.59	Valid	30.61	30.61	Valid	31.42	31.34	Valid	32.10	31.74	Valid	32.53	32.17	Valid	34.53	34.54
		**4**	Valid	N.D	N.D	Valid	30.85	30.70	Valid	30.13	30.44	Valid	31.38	31.11	Valid	31.38	31.67	Valid	32.80	31.98	Valid	33.62	33.18
	2	**5**	Valid	N.D	N.D	Valid	31.02	30.84	Valid	31.30	31.26	Valid	31.07	31.57	Valid	32.24	32.12	Valid	32.45	33.00	Valid	35.76	N.D
		**6**	Valid	N.D	N.D	Valid	30.89	31.03	Valid	31.86	31.80	Valid	31.40	31.47	Valid	32.87	32.20	Valid	33.04	32.91	Valid	33.32	33.99
		**7**	Valid	N.D	N.D	Valid	31.07	31.22	Valid	31.14	31.27	Valid	31.54	31.34	Valid	32.03	32.46	Valid	32.57	32.10	Valid	33.23	33.25
		**8**	Valid	N.D	N.D	Valid	31.06	31.06	Valid	31.23	31.5	Valid	31.18	31.58	Valid	32.03	32.35	Valid	33.87	32.76	Valid	34.02	34.18
	3	**9**	Valid	N.D	N.D	Valid	30.84	30.78	Valid	31.04	31.08	Valid	31.04	31.17	Valid	31.55	31.52	Valid	32.54	32.35	Valid	33.66	33.66
		**10**	Valid	N.D	N.D	Valid	31.06	30.59	Valid	31.76	31.31	Valid	31.63	31.30	Valid	32.04	31.45	Valid	32.57	32.2	Valid	34.16	33.68
		**11**	Valid	N.D	N.D	Valid	31.08	30.95	Valid	30.98	30.97	Valid	31.78	31.68	Valid	32.42	32.08	Valid	32.91	32.49	Valid	35.02	34.96
		**12**	Valid	N.D	N.D	Valid	31.17	31.06	Valid	30.43	30.8	Valid	31.72	31.39	Valid	31.74	32.03	Valid	33.16	32.26	Valid	34.03	33.51
	4	**13**	Valid	N.D	N.D	Valid	30.62	30.36	Valid	32.07	31.18	Valid	31.01	31.48	Valid	31.58	31.05	Valid	33.35	32.77	Valid	33.11	N.D
		**14**	Valid	N.D	N.D	Valid	30.88	30.73	Valid	31.25	31.71	Valid	31.31	31.17	Valid	31.52	31.53	Valid	33.34	N.D	Valid	32.48	32.36
		**15**	Valid	N.D	N.D	Valid	30.83	30.70	Valid	31.12	31.09	Valid	31.57	31.31	Valid	31.93	31.34	Valid	N.D	33.1	Valid	32.21	32.35
		**16**	Valid	N.D	N.D	Valid	31.31	31.02	Valid	31.23	31.14	Valid	31.79	31.15	Valid	32.03	31.77	Valid	33.01	33.19	Valid	32.47	32.82
	5	**17**	Valid	N.D	N.D	Valid	31.06	30.79	Valid	30.77	31.07	Valid	31.05	31.40	Valid	31.11	32.00	Valid	32.25	32.05	Valid	N.D	33.15
		**18**	Valid	N.D	N.D	Valid	30.07	30.79	Valid	30.25	30.50	Valid	31.10	31.04	Valid	31.41	32.46	Valid	32.10	32.74	Valid	32.77	34.77
		**19**	Valid	N.D	N.D	Valid	30.18	30.81	Valid	30.98	31.34	Valid	31.38	31.69	Valid	31.48	31.77	Valid	32.19	32.36	Valid	32.94	33.04
		**20**	Valid	N.D	N.D	Valid	30.24	30.31	Valid	30.39	31.08	Valid	30.63	31.05	Valid	31.49	32.12	Valid	32.16	32.17	Valid	36.72	33.28
**2003G**	1	**1**	Valid	N.D	N.D	Valid	31.03	31.00	Valid	31.23	31.05	Valid	31.64	31.66	Valid	31.85	31.48	Valid	32.67	32.70	Valid	33.97	N.D
		**2**	Valid	N.D	37.88	Valid	30.78	30.63	Valid	31.75	31.53	Valid	31.60	31.14	Valid	32.15	31.96	Valid	32.57	32.11	Valid	33.66	33.85
		**3**	Valid	N.D	N.D	Valid	31.08	30.78	Valid	30.99	31.41	Valid	31.33	31.13	Valid	31.16	31.50	Valid	32.09	N.D	Valid	33.53	33.79
		**4**	Valid	N.D	N.D	Valid	30.77	30.78	Valid	31.20	30.97	Valid	31.20	31.24	Valid	31.57	31.52	Valid	32.46	32.57	Valid	34.43	35.46
	2	**5**	Valid	N.D	N.D	Valid	31.22	31.22	Valid	30.86	31.56	Valid	31.87	31.77	Valid	31.79	32.28	Valid	32.15	33.01	Valid	33.74	34.32
		**6**	Valid	N.D	N.D	Valid	31.49	31.34	Valid	31.86	31.59	Valid	31.50	31.98	Valid	31.64	32.33	Valid	32.59	32.89	Valid	34.57	34.02
		**7**	Valid	N.D	N.D	Valid	31.39	31.31	Valid	30.74	30.92	Valid	32.14	31.79	Valid	31.78	32.01	Valid	32.50	33.02	Valid	33.5	33.68
		**8**	Valid	N.D	N.D	Valid	31.10	31.03	Valid	31.34	31.42	Valid	31.43	31.44	Valid	N.D	31.85	Valid	33.22	33.83	Valid	N.D	33.12
	3	**9**	Valid	N.D	N.D	Valid	31.35	31.30	Valid	31.55	31.34	Valid	32.04	32.04	Valid	32.19	31.83	Valid	33.07	33.08	Valid	34.32	33.42
		**10**	Valid	N.D	N.D	Valid	31.12	31.02	Valid	32.13	31.90	Valid	32.00	31.44	Valid	32.51	32.26	Valid	32.99	32.41	Valid	34.07	34.2

		**11**	Valid	N.D	N.D	Valid	31.39	31.12	Valid	31.26	31.74	Valid	31.68	31.43	Valid	31.47	31.86	Valid	32.40	32.17	Valid	33.95	34.15
		**12**	Valid	N.D	N.D	Valid	31.12	31.12	Valid	31.51	31.27	Valid	31.52	31.54	Valid	31.97	31.89	Valid	32.84	32.93	Valid	34.88	35.87
	4	**13**	Valid	N.D	N.D	Valid	30.31	31.01	Valid	30.31	30.75	Valid	31.30	31.30	Valid	31.15	31.41	Valid	31.73	32.16	Valid	33.82	33.94
		**14**	Valid	N.D	N.D	Valid	30.14	30.92	Valid	30.74	31.35	Valid	30.63	31.12	Valid	31.02	31.51	Valid	32.42	32.96	Valid	32.83	N.D
		**15**	Valid	N.D	N.D	Valid	30.51	30.91	Valid	30.15	30.38	Valid	31.14	31.39	Valid	31.35	31.09	Valid	32.24	32.35	Valid	36.56	N.D
		**16**	Valid	N.D	N.D	Valid	30.30	30.67	Valid	30.32	31.03	Valid	31.07	31.52	Valid	31.69	32.73	Valid	33.09	32.21	Valid	32.76	33.51
	5	**17**	Valid	N.D	N.D	Valid	30.93	30.81	Valid	30.83	31.08	Valid	31.46	31.55	Valid	31.38	32.07	Valid	32.48	32.28	Valid	33.77	33.03
		**18**	Valid	N.D	N.D	Valid	31.13	31.05	Valid	31.25	31.15	Valid	31.76	31.63	Valid	31.64	31.59	Valid	32.93	32.81	Valid	N.D	33.34
		**19**	Valid	N.D	N.D	Valid	31.10	31.27	Valid	31.48	31.12	Valid	31.81	32.02	Valid	32.56	32.13	Valid	32.45	33.03	Valid	33.73	33.15
		**20**	Valid	N.D	N.D	Valid	30.75	30.75	Valid	31.15	31.37	Valid	31.35	31.82	Valid	32.85	32.78	Valid	32.55	32.49	Valid	33.53	33.46

**Lot**	**Day**	**Matrix**	**Swab**																				
			**Neg**			**6 copies/μℓ**			**5 copies/μℓ**			**4 copies/μℓ**			**3 copies/μℓ**			**2 copies/μℓ**			**1 copies/μℓ**		
		**Repl**	**IPC**	**Pan-Sarbecovirus gene**	**SARS-CoV-2 gene**	**IPC**	**Pan-Sarbecovirus gene**	**SARS-CoV-2 gene**	**IPC**	**Pan-Sarbecovirus gene**	**SARS-CoV-2 gene**	**IPC**	**Pan-Sarbecovirus gene**	**SARS-CoV-2 gene**	**IPC**	**Pan-Sarbecovirus gene**	**SARS-CoV-2 gene**	**IPC**	**Pan-Sarbecovirus gene**	**SARS-CoV-2 gene**	**IPC**	**Pan-Sarbecovirus gene**	**SARS-CoV-2 gene**
**2001G**	1	**1**	Valid	N.D	N.D	Valid	30.24	29.95	Valid	30.38	30.06	Valid	30.66	30.68	Valid	31.11	30.69	Valid	32.18	32.17	Valid	33.16	32.81
		**2**	Valid	N.D	N.D	Valid	30.13	29.95	Valid	30.92	30.64	Valid	31.33	30.72	Valid	31.57	30.87	Valid	31.52	31.53	Valid	32.83	32.82
		**3**	Valid	N.D	N.D	Valid	30.29	30.07	Valid	30.63	29.97	Valid	31.23	30.65	Valid	31.36	30.99	Valid	32.22	31.69	Valid	34.04	33.09
		**4**	Valid	N.D	N.D	Valid	30.56	29.90	Valid	30.69	30.53	Valid	31.17	30.73	Valid	31.65	30.84	Valid	31.93	31.95	Valid	33.13	32.64
	2	**5**	Valid	N.D	N.D	Valid	30.07	30.35	Valid	30.26	30.38	Valid	30.44	30.68	Valid	N.D	31.19	Valid	31.53	31.31	Valid	N.D	N.D
		**6**	Valid	N.D	N.D	Valid	30.49	30.31	Valid	30.60	30.27	Valid	30.52	30.97	Valid	31.1	31.12	Valid	N.D	31.50	Valid	32.95	32.74
		**7**	Valid	N.D	N.D	Valid	30.27	30.01	Valid	30.37	30.14	Valid	31.04	30.71	Valid	30.99	30.96	Valid	32.27	31.88	Valid	33.31	32.87
		**8**	Valid	N.D	N.D	Valid	30.09	30.09	Valid	30.73	30.44	Valid	30.85	30.91	Valid	30.89	31.12	Valid	32.00	31.48	Valid	32.47	32.82
	3	**9**	Valid	N.D	34.01	Valid	30.28	30.75	Valid	30.47	30.8	Valid	30.68	31.11	Valid	31.18	31.58	Valid	31.83	31.74	Valid	33.00	32.85
		**10**	Valid	N.D	N.D	Valid	30.76	30.72	Valid	30.88	30.67	Valid	30.78	31.34	Valid	31.33	31.51	Valid	31.74	31.99	Valid	33.21	33.18
		**11**	Valid	N.D	N.D	Valid	30.51	30.35	Valid	30.59	30.49	Valid	31.26	31.13	Valid	31.21	31.33	Valid	32.55	32.30	Valid	33.6	33.27
		**12**	Valid	N.D	N.D	Valid	30.29	30.43	Valid	31.02	30.87	Valid	31.11	31.30	Valid	31.14	31.52	Valid	32.26	31.94	Valid	N.D	33.25
	4	**13**	Valid	N.D	N.D	Valid	30.96	30.89	Valid	31.14	30.84	Valid	31.41	31.25	Valid	31.77	31.37	Valid	32.02	31.79	Valid	32.87	32.45

		**14**	Valid	N.D	N.D	Valid	30.88	30.40	Valid	31.07	30.76	Valid	31.25	31.05	Valid	31.54	31.19	Valid	32.19	31.85	Valid	33.43	32.71
		**15**	Valid	N.D	N.D	Valid	30.65	30.45	Valid	31.01	30.94	Valid	31.31	31.01	Valid	31.73	31.66	Valid	31.42	31.46	Valid	33.21	32.92
		**16**	Valid	N.D	N.D	Valid	30.99	30.43	Valid	31.23	31.03	Valid	31.16	31.04	Valid	31.82	31.23	Valid	32.07	31.88	Valid	33.68	33.13
	5	**17**	Valid	N.D	N.D	Valid	30.47	30.16	Valid	30.75	30.49	Valid	31.22	31.11	Valid	31.78	31.40	Valid	31.89	31.78	Valid	33.18	33.33
		**18**	Valid	37.05	N.D	Valid	30.60	30.57	Valid	31.18	30.8	Valid	31.27	31.29	Valid	31.57	31.25	Valid	31.71	32.08	Valid	33.10	33.15
		**19**	Valid	N.D	N.D	Valid	30.35	30.31	Valid	30.54	30.35	Valid	31.24	31.17	Valid	31.56	31.29	Valid	32.45	32.03	Valid	33.08	33.06
		**20**	Valid	N.D	N.D	Valid	30.47	30.30	Valid	31.09	30.73	Valid	31.29	31.12	Valid	31.42	31.37	Valid	31.50	31.70	Valid	33.09	33.24
**2002G**	1	**1**	Valid	N.D	N.D	Valid	30.44	30.05	Valid	30.78	30.33	Valid	31.30	31.31	Valid	31.52	30.99	Valid	32.02	32.13	Valid	33.13	32.66
		**2**	Valid	N.D	N.D	Valid	30.27	30.26	Valid	30.90	30.71	Valid	31.09	30.88	Valid	31.69	30.98	Valid	32.43	31.75	Valid	34.23	32.74
		**3**	Valid	N.D	N.D	Valid	30.47	30.29	Valid	31.41	30.73	Valid	31.41	31.04	Valid	31.37	31.40	Valid	31.88	31.61	Valid	N.D	32.93
		**4**	Valid	N.D	N.D	Valid	30.56	30.14	Valid	31.19	30.76	Valid	31.52	30.91	Valid	32.28	31.98	Valid	32.02	31.75	Valid	33.20	33.25
	2	**5**	Valid	N.D	N.D	Valid	30.12	30.58	Valid	30.74	30.77	Valid	30.98	31.39	Valid	31.68	31.49	Valid	N.D	32.20	Valid	33.01	33.52
		**6**	Valid	N.D	35.07	Valid	30.35	30.38	Valid	30.63	30.54	Valid	31.06	31.12	Valid	31.39	31.20	Valid	31.84	32.02	Valid	33.16	33.14
		**7**	Valid	N.D	N.D	Valid	30.29	30.37	Valid	30.62	31.04	Valid	31.07	31.17	Valid	31.43	31.28	Valid	31.80	31.88	Valid	33.10	32.51
		**8**	Valid	N.D	N.D	Valid	30.34	30.19	Valid	30.93	30.91	Valid	30.63	30.79	Valid	31.54	31.67	Valid	31.89	31.91	Valid	32.56	33.32
	3	**9**	Valid	N.D	N.D	Valid	30.35	31.05	Valid	31.03	31.21	Valid	31.23	31.85	Valid	N.D	31.99	Valid	32.04	32.68	Valid	33.31	N.D
		**10**	Valid	N.D	N.D	Valid	30.61	30.84	Valid	30.93	31.02	Valid	31.31	31.55	Valid	31.66	31.63	Valid	32.18	32.46	Valid	33.48	33.60
		**11**	Valid	N.D	N.D	Valid	30.54	30.83	Valid	30.91	31.43	Valid	31.32	31.60	Valid	31.74	31.73	Valid	32.14	32.35	Valid	33.41	33.02
		**12**	Valid	N.D	N.D	Valid	30.60	30.60	Valid	31.19	31.31	Valid	30.94	31.23	Valid	31.88	32.16	Valid	32.20	32.38	Valid	32.90	33.77
	4	**13**	Valid	N.D	N.D	Valid	30.32	30.44	Valid	30.43	30.53	Valid	30.93	31.03	Valid	31.42	31.87	Valid	32.10	31.89	Valid	32.2	N.D
		**14**	Valid	N.D	N.D	Valid	30.41	30.23	Valid	30.52	30.53	Valid	30.99	31.08	Valid	31.22	31.34	Valid	31.79	31.79	Valid	33.24	N.D
		**15**	Valid	N.D	N.D	Valid	30.39	30.31	Valid	31.03	30.59	Valid	31.1	30.77	Valid	31.21	31.19	Valid	31.72	N.D	Valid	32.88	32.53
		**16**	Valid	N.D	N.D	Valid	30.16	30.19	Valid	30.67	30.58	Valid	31.01	30.87	Valid	31.34	31.13	Valid	31.2	31.45	Valid	32.5	32.98
	5	**17**	Valid	N.D	N.D	Valid	30.58	30.54	Valid	31.11	31.21	Valid	32.02	31.47	Valid	31.86	31.52	Valid	32.21	32.31	Valid	34.17	33.43
		**18**	Valid	N.D	N.D	Valid	31.12	30.62	Valid	30.89	30.72	Valid	31.74	31.42	Valid	31.86	31.91	Valid	33.03	32.30	Valid	32.98	33.19
		**19**	Valid	36.86	N.D	Valid	30.71	30.45	Valid	31.23	30.94	Valid	31.75	31.43	Valid	32.10	31.88	Valid	32.41	32.29	Valid	34.31	33.09
		**20**	Valid	N.D	N.D	Valid	30.93	30.50	Valid	31.83	31.07	Valid	31.66	31.63	Valid	32.12	31.82	Valid	32.11	32.24	Valid	33.33	33.44
**2003G**	1	**1**	Valid	N.D	N.D	Valid	30.60	30.31	Valid	31.08	30.66	Valid	31.08	31.16	Valid	31.92	31.28	Valid	32.19	N.D	Valid	34.05	33.28
		**2**	Valid	N.D	35.03	Valid	30.53	30.23	Valid	30.95	30.51	Valid	31.43	31.11	Valid	31.33	31.40	Valid	32.10	31.55	Valid	33.57	32.78

		**3**	Valid	N.D	N.D	Valid	30.50	30.11	Valid	31.30	30.81	Valid	31.76	31.13	Valid	31.26	30.99	Valid	32.48	31.69	Valid	33.46	32.48
		**4**	Valid	N.D	N.D	Valid	30.40	30.17	Valid	31.17	30.68	Valid	31.3	31.43	Valid	31.68	31.15	Valid	32.08	31.88	Valid	N.D	32.89
	2	**5**	Valid	N.D	N.D	Valid	30.43	30.57	Valid	30.51	30.87	Valid	31.46	31.08	Valid	31.22	31.36	Valid	31.52	31.86	Valid	33.26	32.59
		**6**	Valid	N.D	N.D	Valid	30.47	30.51	Valid	30.71	30.42	Valid	31.18	31.1	Valid	31.35	31.9	Valid	31.70	31.68	Valid	33.41	33.59
		**7**	Valid	N.D	N.D	Valid	30.26	30.45	Valid	30.56	30.72	Valid	30.93	31.06	Valid	32.00	N.D	Valid	31.97	N.D	Valid	32.62	33.00
		**8**	Valid	N.D	N.D	Valid	30.22	30.30	Valid	31.07	30.77	Valid	30.87	30.79	Valid	31.2	31.08	Valid	31.79	31.98	Valid	33.35	N.D
	3	**9**	Valid	N.D	N.D	Valid	30.73	31.06	Valid	30.82	31.34	Valid	31.73	31.49	Valid	31.47	31.83	Valid	31.81	32.30	Valid	33.51	33.07
		**10**	Valid	N.D	N.D	Valid	30.78	31.01	Valid	31.05	30.94	Valid	31.42	31.54	Valid	31.62	32.34	Valid	32.00	32.15	Valid	33.68	N.D
		**11**	Valid	N.D	N.D	Valid	30.54	30.95	Valid	30.83	31.16	Valid	31.18	31.45	Valid	32.71	31.13	Valid	32.22	32.07	Valid	32.93	33.39
		**12**	Valid	N.D	N.D	Valid	30.49	30.76	Valid	31.33	31.23	Valid	31.12	31.23	Valid	31.45	31.49	Valid	32.07	32.41	Valid	33.62	33.09
	4	**13**	Valid	N.D	N.D	Valid	30.53	30.24	Valid	30.89	30.54	Valid	31.41	31.54	Valid	31.64	31.19	Valid	32.14	32.34	Valid	33.27	32.96
		**14**	Valid	N.D	N.D	Valid	30.34	30.46	Valid	31.01	30.96	Valid	31.18	31.11	Valid	31.82	31.17	Valid	32.58	32.02	Valid	N.D	33.03
		**15**	Valid	N.D	N.D	Valid	30.56	30.50	Valid	31.53	31.00	Valid	31.53	31.23	Valid	31.49	31.62	Valid	32.03	31.88	Valid	33.91	33.17
		**16**	Valid	N.D	N.D	Valid	30.66	30.32	Valid	31.29	31.01	Valid	31.64	31.12	Valid	32.42	32.2	Valid	32.13	32.02	Valid	33.34	33.50
	5	**17**	Valid	N.D	N.D	Valid	30.59	30.23	Valid	31.07	30.58	Valid	31.42	31.24	Valid	31.65	31.59	Valid	31.82	32.16	Valid	33.45	32.72
		**18**	Valid	N.D	N.D	Valid	30.68	30.65	Valid	31.43	30.88	Valid	31.47	31.64	Valid	31.55	31.6	Valid	32.32	31.94	Valid	33.59	32.68
		**19**	Valid	N.D	N.D	Valid	30.84	30.70	Valid	31.10	31.10	Valid	31.30	31.22	Valid	32.10	31.55	Valid	N.D	31.79	Valid	33.83	32.93
		**20**	Valid	N.D	N.D	Valid	30.77	30.40	Valid	31.38	30.99	Valid	31.48	30.86	Valid	32.04	31.80	Valid	32.25	32.23	Valid	33.52	32.86

**Lot**	**Day**	**Matrix**	**Saliva**																				
			**Neg**			**6 copies/㎕**			**5 copies/㎕**			**4 copies/㎕**			**3 copies/㎕**			**2 copies/㎕**			**1 copies/㎕**		
		**Repl**	**IPC**	**Pan-Sarbe coviru s gene**	**SARS-CoV-2 gene**	**IPC**	**Pan-Sarbe coviru s gene**	**SARS-CoV-2 gene**	**IPC**	**Pan-Sarbe coviru s gene**	**SARS-CoV-2 gene**	**IPC**	**Pan-Sarbe coviru s gene**	**SARS-CoV-2 gene**	**IPC**	**Pan-Sarbe coviru s gene**	**SARS-CoV-2 gene**	**IPC**	**Pan-Sarbe coviru s gene**	**SARS-CoV-2 gene**	**IPC**	**Pan-Sarbe coviru s gene**	**SARS-CoV-2 gene**
**2101D**	1	**1**	Valid	N.D	N.D	Valid	30.16	29.41	Valid	30.62	29.70	Valid	30.51	30.93	Valid	31.64	31.4	Valid	32.07	31.39	Valid	32.18	32.4
		**2**	Valid	N.D	N.D	Valid	30.18	30.77	Valid	30.33	30.5	Valid	30.58	31.86	Valid	31.11	30.3	Valid	N.D	31.44	Valid	33.82	32.94
		**3**	Valid	N.D	N.D	Valid	30.14	29.93	Valid	30.55	30.82	Valid	31.38	30.96	Valid	31.74	31.00	Valid	31.75	31.66	Valid	N.D	N.D
		**4**	Valid	N.D	N.D	Valid	30.01	30.29	Valid	30.25	30.07	Valid	31.36	30.99	Valid	31.98	31.48	Valid	31.28	31.03	Valid	34.79	32.63
	2	**5**	Valid	N.D	N.D	Valid	29.49	29.65	Valid	29.91	30.34	Valid	31.20	30.80	Valid	30.90	30.94	Valid	31.44	31.49	Valid	32.72	32.01

		**6**	Valid	N.D	N.D	Valid	30.2	29.61	Valid	30.22	30.21	Valid	30.77	30.37	Valid	30.88	31.03	Valid	32.41	31.62	Valid	33.59	32.30
		**7**	Valid	N.D	N.D	Valid	29.17	30.00	Valid	30.00	30.37	Valid	30.28	30.61	Valid	30.86	30.71	Valid	31.14	31.73	Valid	N.D	33.32
		**8**	Valid	N.D	N.D	Valid	29.19	30.29	Valid	29.72	30.26	Valid	30.48	30.33	Valid	30.98	30.81	Valid	31.73	31.44	Valid	32.46	32.55
	3	**9**	Valid	N.D	N.D	Valid	30.14	30.04	Valid	30.16	30.38	Valid	31.09	31.03	Valid	31.23	31.23	Valid	32.31	31.40	Valid	32.20	32.80
		**10**	Valid	N.D	N.D	Valid	30.04	30.07	Valid	30.78	30.46	Valid	30.63	30.65	Valid	31.22	31.08	Valid	32.21	31.63	Valid	33.27	32.31
		**11**	Valid	N.D	N.D	Valid	30.04	29.91	Valid	30.6	30.79	Valid	31.30	30.77	Valid	31.00	31.08	Valid	32.24	31.36	Valid	33.41	32.12
		**12**	Valid	N.D	N.D	Valid	30.11	30.25	Valid	30.77	30.80	Valid	30.89	30.62	Valid	31.24	30.72	Valid	31.77	31.68	Valid	34.27	32.56
	4	**13**	Valid	N.D	N.D	Valid	28.36	29.31	Valid	29.24	29.89	Valid	30.09	30.29	Valid	30.57	30.91	Valid	31.15	32.43	Valid	32.62	N.D
		**14**	Valid	N.D	N.D	Valid	29.04	30.18	Valid	29.79	29.94	Valid	30.25	30.53	Valid	30.07	31.29	Valid	31.23	31.61	Valid	32.23	32.21
		**15**	Valid	N.D	N.D	Valid	29.55	29.33	Valid	29.66	29.58	Valid	30.04	30.4	Valid	31.21	30.89	Valid	31.66	31.92	Valid	33.64	32.86
		**16**	Valid	N.D	N.D	Valid	29.19	29.64	Valid	29.18	29.74	Valid	30.05	30.10	Valid	31.14	30.66	Valid	32.03	31.48	Valid	32.74	32.58
	5	**17**	Valid	N.D	34.43	Valid	30.45	29.98	Valid	30.53	30.37	Valid	30.92	30.34	Valid	32.00	31.83	Valid	31.34	31.43	Valid	32.50	32.97
		**18**	Valid	N.D	N.D	Valid	29.52	30.06	Valid	30.37	30.09	Valid	31.59	30.81	Valid	31.69	31.08	Valid	32.45	32.06	Valid	34.13	N.D
		**19**	Valid	N.D	N.D	Valid	30.78	30.42	Valid	30.53	30.09	Valid	31.20	30.58	Valid	31.05	30.41	Valid	32.53	N.D	Valid	37.74	32.57
		**20**	Valid	N.D	N.D	Valid	30.43	30.13	Valid	30.96	30.17	Valid	31.72	30.37	Valid	32.19	N.D	Valid	32.27	32.62	Valid	N.D	32.55
**2102D**	1	**1**	Valid	N.D	N.D	Valid	30.07	30.11	Valid	30.72	30.87	Valid	31.02	30.59	Valid	31.60	31.57	Valid	32.96	31.57	Valid	33.96	32.89
		**2**	Valid	N.D	N.D	Valid	30.55	30.03	Valid	30.16	30.76	Valid	31.29	30.94	Valid	31.57	31.16	Valid	32.41	31.60	Valid	33.63	33.45
		**3**	Valid	N.D	N.D	Valid	29.77	30.22	Valid	30.54	30.48	Valid	31.10	30.85	Valid	31.14	31.09	Valid	32.11	N.D	Valid	33.60	N.D
		**4**	Valid	N.D	N.D	Valid	30.80	30.20	Valid	30.68	30.17	Valid	31.01	30.93	Valid	32.18	30.59	Valid	31.97	31.70	Valid	32.35	32.09
	2	**5**	Valid	N.D	N.D	Valid	29.46	29.91	Valid	30.20	30.50	Valid	30.43	30.70	Valid	31.46	30.85	Valid	31.86	31.47	Valid	33.25	33.06
		**6**	Valid	N.D	N.D	Valid	29.66	30.23	Valid	29.92	30.23	Valid	30.37	31.04	Valid	31.20	30.94	Valid	31.82	31.3	Valid	N.D	32.58
		**7**	Valid	N.D	N.D	Valid	30.14	29.48	Valid	30.37	30.12	Valid	30.91	30.87	Valid	31.14	30.87	Valid	32.14	31.48	Valid	32.99	32.83
		**8**	Valid	N.D	N.D	Valid	29.36	29.83	Valid	30.36	30.67	Valid	30.97	30.49	Valid	31.34	31.28	Valid	31.78	31.26	Valid	32.33	32.00
	3	**9**	Valid	N.D	N.D	Valid	29.57	30.12	Valid	30.50	30.32	Valid	30.84	30.93	Valid	31.56	31.62	Valid	32.70	31.55	Valid	34.14	32.34
		**10**	Valid	N.D	N.D	Valid	29.96	30.24	Valid	30.32	30.69	Valid	31.18	30.59	Valid	31.81	30.92	Valid	32.33	31.52	Valid	32.79	32.58
		**11**	Valid	35.47	N.D	Valid	29.77	29.65	Valid	30.39	30.56	Valid	31.17	30.89	Valid	31.52	31.42	Valid	32.15	31.32	Valid	34.47	32.46
		**12**	Valid	N.D	N.D	Valid	30.19	29.81	Valid	30.78	30.98	Valid	31.07	30.61	Valid	31.75	31.31	Valid	31.89	32.67	Valid	33.19	32.05
	4	**13**	Valid	N.D	N.D	Valid	29.40	29.22	Valid	29.41	30.28	Valid	30.20	30.72	Valid	30.97	31.31	Valid	32.5	31.58	Valid	33.46	33.04
		**14**	Valid	N.D	N.D	Valid	28.69	29.40	Valid	29.76	30.34	Valid	30.61	30.77	Valid	30.63	31.17	Valid	31.64	31.92	Valid	32.54	32.87

		**15**	Valid	N.D	N.D	Valid	29.12	29.40	Valid	30.22	30.22	Valid	30.32	30.76	Valid	30.77	31.36	Valid	31.04	31.83	Valid	34.44	32.24
		**16**	Valid	N.D	N.D	Valid	29.6	29.8	Valid	29.68	30.24	Valid	30.75	31.02	Valid	30.69	31.36	Valid	31.25	31.08	Valid	N.D	N.D
	5	**17**	Valid	N.D	N.D	Valid	30.48	29.72	Valid	30.76	30.13	Valid	31.01	30.59	Valid	31.56	30.86	Valid	32.16	32.52	Valid	34.16	N.D
		**18**	Valid	N.D	N.D	Valid	30.03	29.92	Valid	30.44	29.97	Valid	30.83	30.39	Valid	31.69	31.14	Valid	30.93	31.47	Valid	32.85	32.6
		**19**	Valid	N.D	N.D	Valid	30.08	29.65	Valid	30.52	29.81	Valid	31.21	30.46	Valid	30.68	30.97	Valid	32.53	31.65	Valid	33.85	32.35
		**20**	Valid	N.D	N.D	Valid	29.39	30.01	Valid	30.60	29.74	Valid	31.00	30.64	Valid	31.55	30.95	Valid	32.16	31.48	Valid	32.16	32.55
**2103D**	1	**1**	Valid	N.D	N.D	Valid	30.01	30.30	Valid	30.70	30.66	Valid	30.89	31.01	Valid	31.72	30.84	Valid	32.18	31.62	Valid	32.82	32.46
		**2**	Valid	N.D	N.D	Valid	30.08	30.25	Valid	30.50	30.44	Valid	30.91	30.41	Valid	31.62	31.64	Valid	31.51	31.79	Valid	33.07	32.46
		**3**	Valid	N.D	N.D	Valid	30.03	29.64	Valid	30.44	30.48	Valid	31.28	31.06	Valid	31.60	31.22	Valid	31.54	31.41	Valid	33.61	32.24
		**4**	Valid	N.D	N.D	Valid	30.32	29.87	Valid	30.85	29.86	Valid	31.26	30.53	Valid	31.50	31.6	Valid	32.29	31.19	Valid	N.D	33.98
	2	**5**	Valid	N.D	N.D	Valid	29.31	29.67	Valid	30.03	30.12	Valid	30.56	30.49	Valid	30.900	30.81	Valid	31.48	31.15	Valid	32.45	32.02
		**6**	Valid	N.D	N.D	Valid	29.61	30.04	Valid	29.83	30.18	Valid	30.36	30.41	Valid	30.46	30.68	Valid	31.89	31.33	Valid	33.2	32.48
		**7**	Valid	N.D	N.D	Valid	29.89	29.63	Valid	30.5	30.15	Valid	30.24	30.42	Valid	30.8	30.65	Valid	31.85	32.04	Valid	32.05	32.73
		**8**	Valid	N.D	N.D	Valid	29.53	29.73	Valid	29.9	30.08	Valid	30.69	30.57	Valid	31.24	30.45	Valid	31.32	31.77	Valid	32.93	33.17
	3	**9**	Valid	N.D	N.D	Valid	30.27	29.06	Valid	30.50	30.20	Valid	31.05	30.64	Valid	31.37	31.17	Valid	32.46	31.51	Valid	32.62	32.64
		**10**	Valid	N.D	N.D	Valid	30.11	30.09	Valid	30.03	30.21	Valid	30.77	30.86	Valid	31.46	31.03	Valid	31.91	31.57	Valid	34.47	32.44
		**11**	Valid	N.D	N.D	Valid	30.09	29.85	Valid	30.5	30.59	Valid	30.60	30.74	Valid	30.77	31.04	Valid	31.83	31.79	Valid	32.76	32.44
		**12**	Valid	N.D	N.D	Valid	30.11	30.44	Valid	30.91	30.45	Valid	30.98	30.86	Valid	31.29	31.17	Valid	32.54	31.76	Valid	32.76	32.32
	4	**13**	Valid	N.D	N.D	Valid	28.98	29.6	Valid	29.72	30.07	Valid	31.08	30.52	Valid	31.31	31.05	Valid	31.29	31.67	Valid	31.90	32.75
		**14**	Valid	N.D	N.D	Valid	29.00	29.08	Valid	30.35	30.10	Valid	30.88	30.35	Valid	31.16	31.06	Valid	31.47	31.64	Valid	32.53	32.5
		**15**	Valid	N.D	N.D	Valid	29.19	29.29	Valid	30.17	30.14	Valid	30.81	30.43	Valid	30.5	31.9	Valid	31.87	32.35	Valid	32.81	N.D
		**16**	Valid	N.D	N.D	Valid	28.92	29.71	Valid	30.06	30.71	Valid	30.59	30.46	Valid	31.18	31.61	Valid	31.68	31.65	Valid	34.37	32.67
	5	**17**	Valid	N.D	34.23	Valid	29.61	30.03	Valid	30.24	30.08	Valid	31.10	30.75	Valid	31.32	30.74	Valid	N.D	32.36	Valid	32.94	32.78
		**18**	Valid	N.D	N.D	Valid	29.20	30.03	Valid	29.61	29.76	Valid	31.05	30.53	Valid	31.34	31.05	Valid	32.44	32.77	Valid	33.94	32.88
		**19**	Valid	N.D	N.D	Valid	29.60	29.42	Valid	30.88	29.93	Valid	30.83	30.91	Valid	31.22	30.90	Valid	32.07	31.37	Valid	32.54	N.D
		**20**	Valid	N.D	N.D	Valid	30.04	29.49	Valid	30.2	30.12	Valid	30.59	31.21	Valid	31.77	30.63	Valid	32.17	N.D	Valid	N.D	N.D

- *ExiPrep™*48 Dx/ CFX96™ Dx Real-Time PCR Detection System (Bio-Rad)

Lot	Day	Matrix	Sputum																				
			Neg			6 copies/㎕			5 copies/㎕			4 copies/㎕			3 copies/㎕			2 copies/㎕			1 copies/㎕		
		Repl	IPC	Pan-Sarbecovirus gene	SARS-CoV-2 gene	IPC	Pan-Sarbecovirus gene	SARS-CoV-2 gene	IPC	Pan-Sarbecovirus gene	SARS-CoV-2 gene	IPC	Pan-Sarbecovirus gene	SARS-CoV-2 gene	IPC	Pan-Sarbecovirus gene	SARS-CoV-2 gene	IPC	Pan-Sarbecovirus gene	SARS-CoV-2 gene	IPC	Pan-Sarbecovirus gene	SARS-CoV-2 gene
2001G	1	**1**	Valid	ND	ND	Valid	26.16	26.52	Valid	26.59	26.37	Valid	26.69	27.11	Valid	26.85	27.21	Valid	27.63	27.90	Valid	28.34	28.50
		**2**	Valid	ND	ND	Valid	26.26	26.45	Valid	26.19	26.52	Valid	27.24	27.16	Valid	26.99	27.04	Valid	27.65	27.98	Valid	28.32	28.48
		**3**	Valid	ND	ND	Valid	26.16	26.31	Valid	26.29	26.59	Valid	27.11	27.17	Valid	27.16	27.41	Valid	27.47	27.67	Valid	28.23	28.68
		**4**	Valid	ND	ND	Valid	25.54	26.27	Valid	26.31	26.58	Valid	26.74	27.02	Valid	27.06	27.05	Valid	27.44	27.78	Valid	28.23	28.51
	2	**5**	Valid	ND	ND	Valid	26.30	26.33	Valid	26.00	26.38	Valid	26.82	26.19	Valid	26.88	26.88	Valid	27.89	28.13	Valid	28.84	28.38
		**6**	Valid	ND	ND	Valid	26.15	26.39	Valid	26.34	26.45	Valid	26.50	27.11	Valid	26.68	26.87	Valid	27.22	27.45	Valid	27.85	27.78
		**7**	Valid	ND	ND	Valid	25.69	26.26	Valid	26.58	26.63	Valid	26.67	26.50	Valid	26.83	27.37	Valid	27.34	28.02	Valid	27.93	28.71
		**8**	Valid	ND	ND	Valid	26.17	26.22	Valid	26.41	26.66	Valid	27.05	26.74	Valid	26.77	27.25	Valid	27.80	27.79	Valid	29.05	28.56
	3	**9**	Valid	ND	ND	Valid	26.25	25.73	Valid	26.56	26.14	Valid	27.06	26.05	Valid	26.94	26.51	Valid	27.04	26.33	Valid	ND	29.96
		**10**	Valid	ND	ND	Valid	26.62	25.82	Valid	26.67	25.85	Valid	26.61	26.13	Valid	27.64	26.61	Valid	27.04	26.23	Valid	ND	30.69
		**11**	Valid	ND	ND	Valid	26.69	25.76	Valid	27.77	26.41	Valid	26.85	26.24	Valid	28.57	28.13	Valid	26.99	26.17	Valid	ND	30.41
		**12**	Valid	ND	ND	Valid	26.10	25.53	Valid	26.58	26.02	Valid	26.86	26.17	Valid	27.12	27.02	Valid	27.20	26.12	Valid	ND	30.19
	4	**13**	Valid	ND	ND	Valid	26.95	26.06	Valid	26.35	25.93	Valid	27.03	26.12	Valid	27.18	26.90	Valid	28.44	27.15	Valid	29.65	28.30
		**14**	Valid	ND	ND	Valid	26.73	25.87	Valid	26.66	25.94	Valid	27.08	26.38	Valid	27.76	26.81	Valid	27.87	27.63	Valid	28.97	27.41
		**15**	Valid	ND	ND	Valid	26.69	25.88	Valid	28.14	26.12	Valid	27.26	26.23	Valid	29.01	26.85	Valid	28.80	27.79	Valid	29.01	28.62
		**16**	Valid	ND	ND	Valid	26.48	25.70	Valid	26.75	26.13	Valid	27.25	26.54	Valid	27.74	27.07	Valid	28.03	27.54	Valid	29.71	28.35
	5	**17**	Valid	ND	ND	Valid	27.12	26.06	Valid	27.42	26.42	Valid	27.37	27.04	Valid	27.63	26.97	Valid	ND	ND	Valid	ND	ND
		**18**	Valid	ND	ND	Valid	27.27	26.44	Valid	27.31	26.48	Valid	27.14	26.72	Valid	27.54	27.13	Valid	28.37	28.77	Valid	29.03	30.48
		**19**	Valid	ND	ND	Valid	26.23	26.21	Valid	26.90	26.43	Valid	27.59	26.86	Valid	27.90	27.19	Valid	28.71	28.76	Valid	29.39	29.80
		**20**	Valid	ND	ND	Valid	26.87	26.43	Valid	27.13	26.51	Valid	27.05	26.97	Valid	27.91	26.94	Valid	28.95	29.35	Valid	30.11	29.45
**2002G**	1	**1**	Valid	ND	ND	Valid	26.25	26.74	Valid	26.22	26.59	Valid	26.18	26.86	Valid	27.06	27.11	Valid	28.08	28.24	Valid	28.45	28.78

		**2**	Valid	ND	ND	Valid	26.02	26.38	Valid	26.07	26.40	Valid	26.88	27.02	Valid	26.94	27.15	Valid	27.38	27.86	Valid	28.43	28.33
		**3**	Valid	ND	33.77	Valid	26.11	26.35	Valid	26.33	26.41	Valid	26.56	27.08	Valid	26.76	27.39	Valid	28.02	27.86	Valid	28.04	28.51
		**4**	Valid	ND	ND	Valid	26.06	26.23	Valid	26.22	26.61	Valid	27.09	26.87	Valid	26.82	27.08	Valid	27.23	28.04	Valid	28.52	28.64
	2	**5**	Valid	ND	ND	Valid	26.12	26.35	Valid	26.32	26.63	Valid	27.10	27.22	Valid	27.12	27.02	Valid	28.32	28.41	Valid	28.57	28.84
		**6**	Valid	ND	ND	Valid	26.16	26.48	Valid	26.64	26.55	Valid	27.02	26.98	Valid	27.16	26.88	Valid	27.56	28.07	Valid	28.54	28.62
		**7**	Valid	ND	ND	Valid	25.76	26.26	Valid	26.38	26.42	Valid	26.84	27.01	Valid	27.10	27.34	Valid	27.74	27.55	Valid	28.60	29.06
		**8**	Valid	ND	ND	Valid	26.08	26.48	Valid	26.28	26.47	Valid	26.68	26.87	Valid	27.03	27.40	Valid	27.44	27.86	Valid	28.71	28.80
	3	**9**	Valid	ND	ND	Valid	26.26	25.75	Valid	26.62	25.96	Valid	26.96	26.37	Valid	27.64	26.64	Valid	27.41	27.44	Valid	28.95	28.06
		**10**	Valid	ND	ND	Valid	26.31	25.90	Valid	26.88	26.14	Valid	27.24	26.18	Valid	27.35	26.84	Valid	28.09	27.17	Valid	28.77	28.06
		**11**	Valid	ND	ND	Valid	26.39	25.54	Valid	27.42	26.56	Valid	27.06	26.19	Valid	28.61	28.94	Valid	28.03	27.37	Valid	28.61	28.94
		**12**	Valid	ND	ND	Valid	26.10	25.69	Valid	27.05	26.13	Valid	26.81	26.23	Valid	27.04	26.99	Valid	28.14	27.45	Valid	29.82	28.08
	4	**13**	Valid	ND	ND	Valid	26.55	26.00	Valid	26.79	25.99	Valid	26.92	26.45	Valid	27.72	26.78	Valid	26.59	26.93	Valid	28.60	28.53
		**14**	Valid	ND	ND	Valid	26.46	25.81	Valid	26.79	26.28	Valid	26.89	26.38	Valid	27.25	26.96	Valid	26.44	27.09	Valid	28.45	28.29
		**15**	Valid	ND	ND	Valid	26.52	26.02	Valid	27.32	26.20	Valid	26.90	26.67	Valid	29.94	26.93	Valid	27.66	27.68	Valid	ND	ND
		**16**	Valid	ND	ND	Valid	26.46	26.09	Valid	26.89	26.32	Valid	27.15	26.21	Valid	27.61	26.79	Valid	27.87	28.23	Valid	ND	ND
	5	**17**	Valid	ND	ND	Valid	26.80	26.27	Valid	27.15	26.43	Valid	27.33	26.75	Valid	28.02	27.28	Valid	ND	ND	Valid	ND	ND
		**18**	Valid	ND	ND	Valid	26.45	26.23	Valid	26.98	26.44	Valid	27.29	26.78	Valid	28.98	27.06	Valid	28.70	29.05	Valid	29.38	30.65
		**19**	Valid	ND	ND	Valid	26.61	26.17	Valid	26.82	26.35	Valid	27.07	26.63	Valid	27.69	27.03	Valid	28.93	28.84	Valid	29.08	30.44
		**20**	Valid	ND	ND	Valid	26.64	26.24	Valid	26.97	26.44	Valid	26.90	26.71	Valid	27.18	26.98	Valid	28.26	28.60	Valid	28.61	29.90
**2003G**	1	**1**	Valid	ND	ND	Valid	25.73	26.25	Valid	26.21	26.58	Valid	26.79	26.85	Valid	26.73	26.56	Valid	27.66	27.68	Valid	27.68	28.51
		**2**	Valid	ND	ND	Valid	25.79	26.39	Valid	26.43	26.72	Valid	26.71	27.07	Valid	27.01	26.57	Valid	27.87	28.23	Valid	28.01	27.89
		**3**	Valid	ND	ND	Valid	26.03	26.44	Valid	26.22	26.43	Valid	26.69	26.73	Valid	26.59	26.93	Valid	27.54	27.67	Valid	28.60	28.53
		**4**	Valid	ND	ND	Valid	26.08	26.34	Valid	26.11	26.58	Valid	26.47	26.88	Valid	26.44	27.09	Valid	27.18	27.88	Valid	28.45	28.29
	2	**5**	Valid	ND	ND	Valid	26.14	26.43	Valid	26.27	26.26	Valid	26.62	27.09	Valid	26.50	27.03	Valid	27.36	28.04	Valid	27.90	28.52
		**6**	Valid	ND	ND	Valid	26.15	26.64	Valid	26.08	26.43	Valid	26.16	27.04	Valid	26.28	26.68	Valid	27.36	27.83	Valid	28.32	29.00
		**7**	Valid	ND	ND	Valid	26.19	26.55	Valid	26.41	26.66	Valid	26.51	27.00	Valid	26.54	27.19	Valid	27.07	27.62	Valid	28.89	28.82
		**8**	Valid	ND	ND	Valid	26.13	26.16	Valid	26.36	26.72	Valid	26.39	26.93	Valid	27.02	27.17	Valid	27.31	27.65	Valid	28.54	28.50
	3	**9**	Valid	ND	33.28	Valid	26.18	25.68	Valid	26.47	26.03	Valid	27.34	26.11	Valid	27.06	26.60	Valid	28.08	27.10	Valid	28.44	28.08

		**10**	Valid	ND	33.94	Valid	26.44	25.70	Valid	26.46	25.84	Valid	26.72	26.22	Valid	27.37	26.33	Valid	27.81	27.46	Valid	28.83	27.44
		**11**	Valid	ND	ND	Valid	26.53	25.73	Valid	27.82	26.95	Valid	27.11	26.20	Valid	28.38	28.34	Valid	28.24	27.22	Valid	28.38	28.34
		**12**	Valid	ND	ND	Valid	26.00	25.73	Valid	26.69	26.11	Valid	26.71	26.02	Valid	27.04	26.74	Valid	27.75	27.11	Valid	28.52	28.35
	4	**13**	Valid	ND	ND	Valid	26.56	26.05	Valid	26.31	25.61	Valid	26.89	26.36	Valid	27.85	26.66	Valid	28.60	27.09	Valid	28.72	28.38
		**14**	Valid	ND	ND	Valid	26.36	25.95	Valid	26.64	26.05	Valid	27.29	26.33	Valid	27.79	26.57	Valid	27.97	28.09	Valid	29.37	28.11
		**15**	Valid	ND	ND	Valid	26.52	26.03	Valid	27.29	26.20	Valid	27.08	26.44	Valid	29.13	27.13	Valid	28.13	27.44	Valid	29.13	28.42
		**16**	Valid	ND	ND	Valid	26.64	25.85	Valid	26.73	26.21	Valid	27.04	26.27	Valid	27.68	26.72	Valid	27.71	28.01	Valid	28.90	28.34
	5	**17**	Valid	ND	ND	Valid	26.66	26.41	Valid	26.89	26.68	Valid	27.33	27.04	Valid	28.09	27.30	Valid	28.77	28.69	Valid	ND	ND
		**18**	Valid	ND	ND	Valid	26.34	26.07	Valid	27.19	26.47	Valid	27.16	26.75	Valid	27.59	27.28	Valid	28.38	29.02	Valid	29.12	29.65
		**19**	Valid	ND	ND	Valid	26.61	26.34	Valid	27.14	26.79	Valid	27.51	26.92	Valid	27.73	27.35	Valid	28.38	29.08	Valid	29.03	30.58
		**20**	Valid	ND	ND	Valid	27.00	26.26	Valid	27.25	26.44	Valid	27.56	26.67	Valid	27.19	26.68	Valid	30.28	28.66	Valid	28.31	29.94

**Lot**	**Day**	**Matrix**	**Swab**																				
		**Repl**	**Neg**			**6 copies/µℓ**			**5 copies/µℓ**			**4 copies/µℓ**			**3 copies/µℓ**			**2 copies/µℓ**			**1 copies/µℓ**		
			**IPC**	**IPC**	**Pan-Sarbecovirus gene**	**SARS-CoV-2 gene**	**IPC**	**Pan-Sarbecovirus gene**	**SARS-CoV-2 gene**	**IPC**	**Pan-Sarbecovirus gene**	**SARS-CoV-2 gene**	**IPC**	**Pan-Sarbecovirus gene**	**SARS-CoV-2 gene**	**IPC**	**Pan-Sarbecovirus gene**	**SARS-CoV-2 gene**	**IPC**	**Pan-Sarbecovirus gene**	**SARS-CoV-2 gene**	**IPC**	**Pan-Sarbecovirus gene**
**2001G**	1	**1**	Valid	ND	ND	Valid	26.25	26.49	Valid	26.39	26.58	Valid	26.63	26.92	Valid	27.30	27.27	Valid	27.60	27.78	Valid	28.79	29.19
		**2**	Valid	ND	ND	Valid	26.15	26.55	Valid	26.33	26.83	Valid	27.01	27.16	Valid	27.28	27.36	Valid	28.02	27.85	Valid	28.70	29.00
		**3**	Valid	ND	ND	Valid	26.24	26.31	Valid	26.67	26.48	Valid	27.19	27.13	Valid	27.45	27.62	Valid	27.43	28.01	Valid	29.12	28.85
		**4**	Valid	ND	ND	Valid	26.14	26.40	Valid	26.71	26.61	Valid	26.83	27.09	Valid	27.47	27.52	Valid	28.09	27.76	Valid	29.44	29.57
	2	**5**	Valid	ND	ND	Valid	26.29	26.25	Valid	26.18	26.60	Valid	26.41	27.09	Valid	26.75	27.09	Valid	27.60	27.81	Valid	29.00	28.59
		**6**	Valid	ND	ND	Valid	26.17	26.59	Valid	26.31	26.39	Valid	26.67	26.92	Valid	26.79	27.34	Valid	27.74	27.58	Valid	28.79	28.96
		**7**	Valid	ND	ND	Valid	26.19	26.23	Valid	26.67	26.71	Valid	27.11	27.16	Valid	27.25	27.78	Valid	27.54	27.79	Valid	27.91	28.75
		**8**	Valid	ND	ND	Valid	26.10	26.13	Valid	26.46	26.54	Valid	26.67	27.04	Valid	27.35	27.45	Valid	27.34	27.81	Valid	28.25	29.27
	3	**9**	Valid	ND	ND	Valid	26.29	25.90	Valid	27.09	26.06	Valid	27.01	26.28	Valid	27.90	26.60	Valid	28.63	27.72	Valid	28.88	28.71
		**10**	Valid	ND	33.61	Valid	26.20	25.88	Valid	27.17	25.99	Valid	27.20	26.33	Valid	27.25	26.40	Valid	28.14	27.12	Valid	29.28	28.47
		**11**	Valid	ND	ND	Valid	26.38	25.84	Valid	27.33	26.13	Valid	27.24	26.15	Valid	28.91	26.51	Valid	28.05	27.18	Valid	28.91	27.67
		**12**	Valid	ND	ND	Valid	26.47	25.86	Valid	27.13	26.09	Valid	27.14	26.34	Valid	27.20	27.04	Valid	28.27	27.33	Valid	29.04	28.48

	4	**13**	Valid	ND	ND	Valid	26.64	25.93	Valid	26.95	25.90	Valid	27.26	26.44	Valid	27.62	26.76	Valid	ND	ND	Valid	ND	ND
		**14**	Valid	ND	ND	Valid	26.17	25.63	Valid	26.93	26.16	Valid	27.23	26.34	Valid	27.37	26.77	Valid	29.09	29.28	Valid	29.33	30.42
		**15**	Valid	ND	ND	Valid	26.56	26.01	Valid	26.96	26.10	Valid	27.32	26.28	Valid	27.44	27.08	Valid	28.87	29.40	Valid	29.47	31.29
		**16**	Valid	ND	ND	Valid	26.36	25.68	Valid	26.90	25.85	Valid	27.11	26.31	Valid	27.27	26.66	Valid	28.25	28.92	Valid	31.49	30.23
	5	**17**	Valid	ND	ND	Valid	26.70	26.31	Valid	27.30	26.69	Valid	27.48	27.06	Valid	27.37	26.79	Valid	28.21	27.61	Valid	29.42	29.07
		**18**	Valid	ND	ND	Valid	27.06	26.05	Valid	27.22	26.40	Valid	27.48	26.98	Valid	28.19	27.03	Valid	28.50	27.90	Valid	28.91	28.62
		**19**	Valid	ND	ND	Valid	26.69	26.51	Valid	27.33	26.33	Valid	27.48	26.87	Valid	28.05	27.48	Valid	28.34	27.45	Valid	29.61	28.39
		**20**	Valid	34.38	ND	Valid	26.72	25.86	Valid	26.97	26.36	Valid	27.33	26.71	Valid	27.77	27.62	Valid	28.47	28.10	Valid	29.66	28.62
**2002G**	1	**1**	Valid	ND	35.78	Valid	26.15	26.52	Valid	26.45	26.33	Valid	26.46	26.84	Valid	27.04	27.70	Valid	27.57	28.08	Valid	28.73	28.89
		**2**	Valid	ND	ND	Valid	26.08	26.37	Valid	26.35	26.77	Valid	26.92	26.92	Valid	27.11	27.30	Valid	28.05	28.11	Valid	28.82	29.10
		**3**	Valid	ND	ND	Valid	25.80	26.21	Valid	26.49	26.59	Valid	26.81	26.91	Valid	27.17	27.25	Valid	28.19	28.45	Valid	29.17	29.04
		**4**	Valid	ND	ND	Valid	26.20	26.49	Valid	26.64	26.54	Valid	26.71	26.83	Valid	27.01	27.06	Valid	27.68	27.67	Valid	29.81	29.27
	2	**5**	Valid	ND	ND	Valid	26.05	26.34	Valid	26.21	26.41	Valid	26.43	24.82	Valid	27.00	27.58	Valid	27.27	27.94	Valid	28.73	28.85
		**6**	Valid	ND	ND	Valid	26.07	26.26	Valid	26.10	26.61	Valid	26.73	25.85	Valid	27.23	27.30	Valid	27.32	27.64	Valid	28.76	29.67
		**7**	Valid	ND	ND	Valid	25.90	26.33	Valid	26.48	26.43	Valid	27.10	26.90	Valid	26.93	27.37	Valid	27.14	27.61	Valid	28.80	29.23
		**8**	Valid	ND	ND	Valid	26.06	26.35	Valid	26.38	26.74	Valid	26.63	26.65	Valid	27.01	27.52	Valid	27.82	28.07	Valid	28.67	29.04
	3	**9**	Valid	ND	ND	Valid	26.04	25.77	Valid	27.00	26.18	Valid	27.46	26.85	Valid	28.00	27.10	Valid	29.07	27.73	Valid	29.20	28.15
		**10**	Valid	ND	ND	Valid	26.60	25.94	Valid	27.06	26.00	Valid	27.17	26.12	Valid	27.85	26.73	Valid	28.56	27.24	Valid	29.17	28.38
		**11**	Valid	ND	ND	Valid	26.20	25.84	Valid	27.45	26.12	Valid	27.09	26.13	Valid	29.42	26.88	Valid	28.26	27.26	Valid	29.42	28.33
		**12**	Valid	ND	ND	Valid	26.72	26.05	Valid	26.96	26.01	Valid	27.14	26.31	Valid	27.77	26.73	Valid	28.26	27.20	Valid	30.48	28.07
	4	**13**	Valid	ND	ND	Valid	26.59	25.70	Valid	26.76	26.03	Valid	27.37	26.43	Valid	27.52	26.56	Valid	ND	ND	Valid	29.32	31.15
		**14**	Valid	ND	ND	Valid	26.55	26.02	Valid	26.97	25.92	Valid	27.06	26.39	Valid	27.38	26.96	Valid	28.53	29.44	Valid	29.54	30.10
		**15**	Valid	ND	ND	Valid	26.68	25.74	Valid	26.37	26.09	Valid	27.12	26.17	Valid	27.64	26.70	Valid	28.57	28.85	Valid	30.01	31.01
		**16**	Valid	ND	ND	Valid	26.41	25.72	Valid	26.96	26.27	Valid	27.20	26.35	Valid	27.29	26.82	Valid	28.77	29.33	Valid	ND	ND
	5	**17**	Valid	ND	ND	Valid	26.81	26.23	Valid	27.05	26.45	Valid	27.22	27.35	Valid	28.02	27.01	Valid	28.20	27.77	Valid	29.18	29.00
		**18**	Valid	ND	ND	Valid	26.85	26.04	Valid	26.92	26.70	Valid	27.21	27.02	Valid	28.06	27.13	Valid	28.08	28.54	Valid	29.37	28.69
		**19**	Valid	ND	ND	Valid	26.77	26.40	Valid	27.01	26.71	Valid	27.20	26.66	Valid	27.30	27.01	Valid	28.89	27.68	Valid	28.76	28.64
		**20**	Valid	ND	ND	Valid	26.75	26.16	Valid	27.37	26.89	Valid	27.23	26.99	Valid	27.51	27.05	Valid	28.25	27.99	Valid	29.31	28.40
**2003G**	1	**1**	Valid	ND	ND	Valid	26.19	26.27	Valid	26.12	26.49	Valid	27.04	26.98	Valid	27.68	27.51	Valid	28.20	27.76	Valid	28.94	28.19

		**2**	Valid	ND	ND	Valid	26.19	26.44	Valid	26.56	26.66	Valid	26.71	27.09	Valid	27.26	27.66	Valid	27.27	27.90	Valid	28.57	29.10
		**3**	Valid	ND	ND	Valid	26.00	26.20	Valid	26.05	26.31	Valid	26.79	27.00	Valid	27.04	27.09	Valid	27.97	27.85	Valid	28.06	29.24
		**4**	Valid	ND	ND	Valid	26.01	26.35	Valid	26.52	26.41	Valid	26.96	27.06	Valid	27.04	27.60	Valid	28.41	27.94	Valid	28.45	29.40
	2	**5**	Valid	33.82	ND	Valid	23.01	22.69	Valid	26.04	26.23	Valid	26.32	26.72	Valid	27.20	26.88	Valid	27.36	27.85	Valid	28.50	28.42
		**6**	Valid	ND	ND	Valid	25.07	24.92	Valid	30.24	31.25	Valid	26.06	25.91	Valid	27.76	26.55	Valid	27.31	28.03	Valid	28.39	28.97
		**7**	Valid	ND	ND	Valid	26.33	26.24	Valid	26.01	26.97	Valid	28.10	35.15	Valid	26.68	27.04	Valid	27.46	28.38	Valid	28.81	29.04
		**8**	Valid	ND	ND	Valid	26.07	26.11	Valid	26.19	26.40	Valid	26.88	27.27	Valid	26.79	27.49	Valid	27.53	27.97	Valid	28.99	28.99
	3	**9**	Valid	ND	ND	Valid	26.48	25.95	Valid	27.03	26.28	Valid	27.04	26.33	Valid	27.62	26.71	Valid	28.16	27.14	Valid	28.98	28.14
		**10**	Valid	ND	ND	Valid	26.30	26.05	Valid	26.50	26.11	Valid	27.04	26.23	Valid	27.52	26.43	Valid	28.04	27.32	Valid	28.41	27.94
		**11**	Valid	ND	ND	Valid	26.22	25.81	Valid	27.26	26.02	Valid	26.99	26.17	Valid	29.67	27.16	Valid	28.02	27.20	Valid	29.28	28.23
		**12**	Valid	ND	ND	Valid	26.52	25.93	Valid	26.95	26.40	Valid	27.20	26.12	Valid	27.29	26.87	Valid	27.54	27.63	Valid	29.28	28.20
	4	**13**	Valid	ND	ND	Valid	26.89	25.82	Valid	27.06	26.18	Valid	27.05	26.25	Valid	27.56	26.94	Valid	28.52	29.24	Valid	ND	ND
		**14**	Valid	ND	ND	Valid	26.83	25.93	Valid	27.17	26.09	Valid	27.23	26.51	Valid	27.63	26.92	Valid	28.89	28.82	Valid	ND	ND
		**15**	Valid	ND	ND	Valid	26.37	25.85	Valid	26.96	26.22	Valid	27.11	26.20	Valid	27.39	26.97	Valid	28.48	29.09	Valid	ND	ND
		**16**	Valid	ND	ND	Valid	26.49	25.96	Valid	26.75	26.13	Valid	27.09	26.12	Valid	27.45	26.57	Valid	28.78	29.28	Valid	29.01	30.05
	5	**17**	Valid	33.92	ND	Valid	26.72	26.22	Valid	27.13	26.63	Valid	27.41	26.93	Valid	27.65	27.18	Valid	28.94	27.80	Valid	29.27	28.75
		**18**	Valid	ND	ND	Valid	26.62	26.05	Valid	27.06	26.41	Valid	27.24	26.64	Valid	27.45	27.15	Valid	28.01	27.48	Valid	29.19	28.01
		**19**	Valid	ND	ND	Valid	26.75	26.42	Valid	26.44	26.58	Valid	27.32	27.49	Valid	27.81	27.26	Valid	27.43	27.74	Valid	29.54	28.92
		**20**	Valid	ND	ND	Valid	27.07	26.46	Valid	27.01	26.76	Valid	27.21	26.83	Valid	28.12	27.06	Valid	28.09	27.24	Valid	29.15	28.17

**Lot**	**Day**	**Matrix**	**Saliva**																				
		**Repl**	**Neg**			**6 copies/㎕**			**5 copies/㎕**			**4 copies/㎕**			**3 copies/㎕**			**2 copies/㎕**			**1 copies/㎕**		
			**IPC**	**Pan-Sarbecovirus gene**	**SARS-CoV-2 gene**	**IPC**	**Pan-Sarbecovirus gene**	**SARS-CoV-2 gene**	**IPC**	**Pan-Sarbecovirus gene**	**SARS-CoV-2 gene**	**IPC**	**Pan-Sarbecovirus gene**	**SARS-CoV-2 gene**	**IPC**	**Pan-Sarbecovirus gene**	**SARS-CoV-2 gene**	**IPC**	**Pan-Sarbecovirus gene**	**SARS-CoV-2 gene**	**IPC**	**Pan-Sarbecovirus gene**	**SARS-CoV-2 gene**
**2101D**	1	**1**	Valid	ND	ND	Valid	27.21	27.88	Valid	27.80	28.07	Valid	28.69	28.26	Valid	28.69	29.40	Valid	29.75	29.62	Valid	29.78	29.56
		**2**	Valid	ND	ND	Valid	27.35	28.21	Valid	27.36	28.16	Valid	28.93	28.78	Valid	28.58	29.55	Valid	29.27	29.29	Valid	29.85	30.58
		**3**	Valid	ND	ND	Valid	27.98	27.61	Valid	27.68	28.07	Valid	27.47	28.32	Valid	27.68	29.12	Valid	30.57	29.27	Valid	29.81	31.00
		**4**	Valid	ND	ND	Valid	27.59	28.10	Valid	28.03	26.01	Valid	27.82	27.99	Valid	28.19	29.06	Valid	28.43	29.80	Valid	30.21	29.71

	2	**5**	Valid	ND	ND	Valid	27.63	28.06	Valid	28.15	28.35	Valid	28.01	28.62	Valid	28.41	29.04	Valid	28.92	29.59	Valid	30.39	30.36
		**6**	Valid	ND	ND	Valid	28.19	28.09	Valid	27.91	28.50	Valid	28.64	27.79	Valid	30.29	28.72	Valid	29.55	29.70	Valid	31.10	30.41
		**7**	Valid	ND	ND	Valid	27.70	26.95	Valid	29.00	28.01	Valid	28.02	28.04	Valid	28.66	28.90	Valid	29.17	29.22	Valid	29.50	29.88
		**8**	Valid	ND	ND	Valid	27.01	28.19	Valid	27.39	28.18	Valid	28.09	28.22	Valid	29.09	28.40	Valid	29.70	29.39	Valid	32.11	29.95
	3	**9**	Valid	ND	ND	Valid	26.58	28.04	Valid	29.65	28.28	Valid	29.26	28.96	Valid	30.05	29.49	Valid	30.92	31.07	Valid	30.20	30.37
		**10**	Valid	ND	ND	Valid	26.21	27.73	Valid	28.75	28.53	Valid	29.16	28.99	Valid	30.50	30.21	Valid	30.54	29.42	Valid	31.76	30.44
		**11**	Valid	ND	ND	Valid	26.62	28.38	Valid	28.82	28.27	Valid	29.87	28.23	Valid	30.00	30.18	Valid	29.38	30.00	Valid	30.93	29.95
		**12**	Valid	ND	ND	Valid	26.22	27.96	Valid	28.09	28.18	Valid	39.59	40.27	Valid	30.15	29.80	Valid	30.15	29.81	Valid	32.31	30.78
	4	**13**	Valid	ND	ND	Valid	28.64	28.23	Valid	28.36	28.04	Valid	28.83	29.06	Valid	29.80	28.91	Valid	29.69	29.21	Valid	30.14	29.09
		**14**	Valid	ND	ND	Valid	28.15	27.88	Valid	28.44	28.35	Valid	29.63	29.05	Valid	29.03	29.74	Valid	29.42	31.24	Valid	30.14	29.39
		**15**	Valid	32.81	ND	Valid	29.07	29.29	Valid	28.90	28.47	Valid	28.52	28.11	Valid	30.28	28.73	Valid	29.98	28.34	Valid	UD	32.60
		**16**	Valid	ND	ND	Valid	28.42	28.05	Valid	28.63	28.10	Valid	29.52	28.46	Valid	31.17	28.72	Valid	UD	30.43	Valid	32.60	33.73
	5	**17**	Valid	ND	ND	Valid	27.15	27.83	Valid	28.11	28.01	Valid	28.04	28.62	Valid	29.26	29.26	Valid	29.24	29.44	Valid	30.94	29.45
		**18**	Valid	ND	ND	Valid	27.49	27.58	Valid	28.00	28.02	Valid	28.42	28.79	Valid	28.31	28.75	Valid	29.52	29.78	Valid	UD	28.00
		**19**	Valid	ND	ND	Valid	27.67	28.15	Valid	27.28	27.47	Valid	28.01	27.22	Valid	28.44	29.26	Valid	29.59	30.18	Valid	31.21	30.39
		**20**	Valid	ND	ND	Valid	27.95	28.04	Valid	27.27	28.32	Valid	28.30	28.00	Valid	28.46	29.26	Valid	28.41	29.05	Valid	31.80	30.15
**2102D**	1	**1**	Valid	ND	ND	Valid	27.35	27.54	Valid	27.88	27.59	Valid	27.71	28.20	Valid	30.06	28.44	Valid	29.62	29.22	Valid	30.59	27.00
		**2**	Valid	ND	ND	Valid	27.47	27.19	Valid	27.68	27.46	Valid	29.51	29.64	Valid	29.50	29.08	Valid	29.70	29.52	Valid	29.99	29.48
		**3**	Valid	ND	ND	Valid	27.12	27.39	Valid	27.41	28.06	Valid	28.08	27.70	Valid	28.38	28.57	Valid	28.61	29.23	Valid	UD	UD
		**4**	Valid	ND	ND	Valid	27.37	27.54	Valid	27.65	28.30	Valid	28.35	28.42	Valid	28.10	28.20	Valid	28.53	28.60	Valid	31.96	30.10
	2	**5**	Valid	ND	ND	Valid	26.43	28.32	Valid	28.70	28.98	Valid	28.71	28.54	Valid	30.01	29.17	Valid	29.83	29.73	Valid	30.37	31.17
		**6**	Valid	ND	ND	Valid	27.26	28.47	Valid	29.61	28.31	Valid	29.32	28.93	Valid	30.35	29.15	Valid	30.21	30.82	Valid	31.40	31.11
		**7**	Valid	ND	ND	Valid	27.03	28.26	Valid	29.09	28.67	Valid	29.21	28.80	Valid	29.21	29.05	Valid	31.32	29.46	Valid	32.57	30.39
		**8**	Valid	ND	33.03	Valid	26.72	28.23	Valid	28.69	28.46	Valid	28.34	28.83	Valid	30.54	29.33	Valid	30.43	30.14	Valid	32.19	30.39
	3	**9**	Valid	ND	ND	Valid	28.69	28.46	Valid	28.98	28.20	Valid	28.41	28.60	Valid	29.99	28.82	Valid	30.29	30.02	Valid	31.04	29.55
		**10**	Valid	ND	ND	Valid	28.68	28.14	Valid	28.21	28.78	Valid	28.56	28.62	Valid	28.81	28.75	Valid	30.58	28.20	Valid	31.58	30.60
		**11**	Valid	ND	ND	Valid	28.36	28.22	Valid	29.41	28.38	Valid	29.04	28.63	Valid	29.20	29.11	Valid	30.68	29.60	Valid	32.40	31.14
		**12**	Valid	ND	ND	Valid	28.30	28.44	Valid	29.42	28.86	Valid	29.75	28.89	Valid	30.03	29.36	Valid	29.72	28.61	Valid	32.46	31.68
	4	**13**	Valid	ND	ND	Valid	26.73	27.12	Valid	27.27	27.20	Valid	27.90	27.40	Valid	28.93	28.49	Valid	29.07	28.89	Valid	32.31	29.13

		**14**	Valid	ND	ND	Valid	28.21	27.52	Valid	27.37	27.33	Valid	27.65	28.06	Valid	28.56	27.13	Valid	30.06	28.34	Valid	30.54	28.55
		**15**	Valid	ND	ND	Valid	27.93	27.47	Valid	27.28	27.05	Valid	28.45	28.04	Valid	28.51	27.40	Valid	29.47	28.11	Valid	30.20	29.67
		**16**	Valid	ND	ND	Valid	27.38	27.05	Valid	27.87	27.26	Valid	24.76	25.12	Valid	29.10	28.07	Valid	28.44	28.25	Valid	29.88	28.09
	5	**17**	Valid	ND	ND	Valid	27.49	28.15	Valid	28.07	28.25	Valid	28.28	28.60	Valid	28.69	29.42	Valid	28.67	29.87	Valid	29.04	30.23
		**18**	Valid	ND	ND	Valid	27.44	27.50	Valid	28.19	28.12	Valid	27.81	28.82	Valid	28.95	29.24	Valid	29.16	29.91	Valid	30.51	28.72
		**19**	Valid	ND	ND	Valid	27.20	28.13	Valid	28.08	27.74	Valid	28.09	27.90	Valid	29.05	28.56	Valid	28.86	28.70	Valid	29.13	30.21
		**20**	Valid	ND	ND	Valid	27.19	27.72	Valid	27.10	28.71	Valid	28.20	28.40	Valid	28.07	26.86	Valid	29.41	28.81	Valid	29.81	29.12
**2103D**	1	**1**	Valid	ND	ND	Valid	27.94	27.70	Valid	30.02	30.35	Valid	28.20	27.99	Valid	28.59	28.13	Valid	28.75	29.08	Valid	30.66	30.08
		**2**	Valid	ND	ND	Valid	27.30	28.17	Valid	27.89	27.76	Valid	27.53	28.14	Valid	28.28	28.23	Valid	29.82	28.96	Valid	30.29	29.43
		**3**	Valid	ND	ND	Valid	27.77	27.22	Valid	27.80	28.09	Valid	31.26	31.25	Valid	28.97	28.92	Valid	29.10	29.41	Valid	UD	UD
		**4**	Valid	ND	ND	Valid	27.47	27.59	Valid	27.40	27.85	Valid	28.04	28.17	Valid	29.33	22.92	Valid	29.89	28.97	Valid	UD	UD
	2	**5**	Valid	ND	ND	Valid	26.19	28.28	Valid	28.53	28.43	Valid	29.61	28.71	Valid	29.90	28.61	Valid	30.41	30.33	Valid	30.67	29.94
		**6**	Valid	ND	ND	Valid	26.64	28.36	Valid	29.95	28.81	Valid	28.93	29.05	Valid	30.16	29.17	Valid	30.14	29.65	Valid	31.26	31.13
		**7**	Valid	ND	ND	Valid	26.41	28.64	Valid	28.20	28.64	Valid	28.45	29.11	Valid	29.75	28.76	Valid	30.59	30.00	Valid	32.91	30.31
		**8**	Valid	ND	ND	Valid	26.83	28.31	Valid	28.76	28.62	Valid	29.01	28.46	Valid	29.52	29.15	Valid	30.01	30.08	Valid	34.02	30.30
	3	**9**	Valid	ND	ND	Valid	28.58	28.31	Valid	28.44	29.14	Valid	28.90	28.12	Valid	29.07	29.64	Valid	30.23	29.43	Valid	31.24	30.28
		**10**	Valid	ND	ND	Valid	28.12	27.97	Valid	28.22	28.42	Valid	28.69	28.73	Valid	29.05	28.83	Valid	29.76	29.44	Valid	30.05	31.42
		**11**	Valid	ND	ND	Valid	28.23	28.10	Valid	28.14	28.50	Valid	28.41	29.18	Valid	29.41	28.61	Valid	30.11	29.24	Valid	32.76	30.42
		**12**	Valid	ND	ND	Valid	27.91	28.30	Valid	28.54	28.05	Valid	28.69	28.17	Valid	29.44	28.61	Valid	29.87	29.95	Valid	30.70	30.32
	4	**13**	Valid	ND	ND	Valid	26.73	27.12	Valid	27.27	27.20	Valid	27.90	27.40	Valid	28.93	28.49	Valid	29.07	28.89	Valid	32.31	29.13
		**14**	Valid	ND	ND	Valid	28.21	27.52	Valid	27.37	27.33	Valid	27.65	28.06	Valid	28.56	27.13	Valid	30.06	28.34	Valid	30.54	28.55
		**15**	Valid	ND	ND	Valid	27.93	27.47	Valid	27.28	27.05	Valid	28.45	28.04	Valid	28.51	27.40	Valid	29.47	28.11	Valid	30.20	29.67
		**16**	Valid	ND	ND	Valid	27.38	27.05	Valid	27.87	27.26	Valid	24.76	25.12	Valid	29.10	28.07	Valid	28.44	28.25	Valid	29.88	28.09
	5	**17**	Valid	ND	ND	Valid	27.82	27.78	Valid	27.37	28.02	Valid	28.10	27.96	Valid	28.23	28.49	Valid	28.60	28.93	Valid	31.13	29.57
		**18**	Valid	ND	ND	Valid	27.60	27.77	Valid	27.49	27.99	Valid	28.11	27.48	Valid	28.16	28.76	Valid	28.81	28.87	Valid	31.26	29.50
		**19**	Valid	ND	ND	Valid	27.06	27.49	Valid	27.81	28.11	Valid	27.76	28.16	Valid	28.26	28.21	Valid	30.51	27.24	Valid	UD	UD
		**20**	Valid	ND	ND	Valid	27.27	27.58	Valid	27.49	28.03	Valid	27.93	28.01	Valid	28.21	28.37	Valid	29.63	28.57	Valid	UD	UD

S4 Table. Limit of detection of SCVM in various PCR instruments.

AccuPower® SARS-CoV-2 Multiplex Real-Time RT-PCR Kit (SCVM)

Instrument	Matrix	Nominal Concentration (copies/㎕)	Number of replicates tested (N)	Pan-Sarbecovirus gene		SARS-CoV-2 gene	
				Number of positives detected (N)	Positive rate	Number of positives detected (N)	Positive rate
**ABI 7500 Fast Real-time PCR Instrument system**	**Sputum**	3	21	21	100%	21	100%
		2	21	21	100%	21	100%
	**Swab**	3	21	21	100%	21	100%
		2	21	21	100%	21	100%
	**Saliva**	2	21	21	100%	21	100%
**QuantStudio™ 5 Real-Time PCR Instrument**	**Sputum**	3	21	21	100%	21	100%
		2	21	20	95%	20	95%
	**Swab**	3	21	21	100%	21	100%
		2	21	21	100%	21	100%
	**Saliva**	2	21	21	100%	21	100%
***Exicycler*™384**	**Sputum**	3	21	21	100%	21	100%
		2	21	21	100%	21	100%
	**Swab**	3	21	21	100%	21	100%
		2	21	21	100%	21	100%
	**Saliva**	2	21	21	100%	21	100%
***Exicycler*™96 V4 Fast**	**Sputum**	3	21	21	100%	21	100%
		2	21	20	95%	21	100%
	**Swab**	3	21	21	100%	21	100%
		2	21	21	100%	21	100%
	**Saliva**	2	21	21	100%	21	100%

[Alternative Instrument Testing summary – Swab, Sputum, Saliva]

Instrument	Pan-Sarbecovirus gene	SARS-CoV-2 gene
ABI 7500 Fast Real-time PCR Instrument system	2 copies/㎕	2 copies/㎕
QuantStudio™ 5 Real-Time PCR Instrument	2 copies/㎕	2 copies/㎕
Exicycler™384	2 copies/㎕	2 copies/㎕
Exicycler™96 V4 Fast	2 copies/㎕	2 copies/㎕

Result of Alternative Instrument Testing

1. Applied Biosystems 7500 Fast Real-time PCR Instrument system, QuantStudio™5 Real-Time PCR Instrument

Sample	Applied Biosystems 7500 Fast Real-time PCR Instrument system									QuantStudio™5 Real-Time PCR Instrument								
	Sputum			Swab			Saliva			Sputum			Swab			Saliva		
	IPC	Pan-Sarbecovirus gene	SARS-CoV-2 gene	IPC	Pan-Sarbecovirus gene	SARS-CoV-2 gene	IPC	Pan-Sarbecovirus gene	SARS-CoV-2 gene	IPC	Pan-Sarbecovirus gene	SARS-CoV-2 gene	IPC	Pan-Sarbecovirus gene	SARS-CoV-2 gene	IPC	Pan-Sarbecovirus gene	SARS-CoV-2 gene
NTC	Valid	N.D	N.D	N/A			Valid	N.D	N.D	Valid	N.D	N.D	N/A			N.D	N.D	N.D
PC	Valid	24.70	23.32				Valid	22.39	22.40	Valid	21.83	22.39				21.45	22.71	21.45
Neg	Valid	N.D	N.D	Valid	N.D	N.D	Valid	N.D	N.D	Valid	N.D	N.D	Valid	N.D	N.D	Valid	N.D	N.D
	Valid	N.D	N.D	Valid	N.D	N.D	Valid	N.D	N.D	Valid	N.D	N.D	Valid	N.D	N.D	Valid	N.D	N.D
	Valid	N.D	N.D	Valid	N.D	N.D	Valid	N.D	N.D	Valid	N.D	N.D	Valid	N.D	N.D	Valid	N.D	N.D
	Valid	N.D	N.D	Valid	N.D	N.D	Valid	N.D	N.D	Valid	37.49	N.D	Valid	N.D	N.D	Valid	N.D	N.D
	Valid	N.D	N.D	Valid	N.D	N.D	Valid	N.D	N.D	Valid	N.D	N.D	Valid	N.D	N.D	Valid	N.D	N.D
	Valid	N.D	N.D	Valid	N.D	N.D	Valid	N.D	N.D	Valid	N.D	N.D	Valid	N.D	N.D	Valid	N.D	N.D
	Valid	N.D	N.D	Valid	N.D	N.D	Valid	N.D	N.D	Valid	N.D	N.D	Valid	N.D	N.D	Valid	N.D	N.D
	Valid	N.D	N.D	Valid	N.D	N.D	Valid	N.D	N.D	Valid	N.D	N.D	Valid	N.D	N.D	Valid	N.D	N.D
	Valid	N.D	N.D	Valid	N.D	N.D	Valid	N.D	N.D	Valid	N.D	N.D	Valid	N.D	N.D	Valid	N.D	N.D
	Valid	N.D	N.D	Valid	N.D	N.D	Valid	N.D	N.D	Valid	N.D	N.D	Valid	N.D	N.D	Valid	N.D	N.D
	Valid	N.D	N.D	Valid	N.D	N.D	Valid	N.D	N.D	Valid	N.D	N.D	Valid	N.D	N.D	Valid	N.D	N.D
	Valid	N.D	N.D	Valid	N.D	N.D	Valid	N.D	N.D	Valid	N.D	N.D	Valid	N.D	N.D	Valid	N.D	N.D
	Valid	N.D	N.D	Valid	N.D	N.D	Valid	N.D	N.D	Valid	N.D	N.D	Valid	N.D	N.D	Valid	N.D	N.D
	Valid	N.D	N.D	Valid	N.D	N.D	Valid	N.D	N.D	Valid	N.D	N.D	Valid	N.D	N.D	Valid	N.D	N.D
	Valid	N.D	N.D	Valid	N.D	N.D	Valid	N.D	N.D	Valid	N.D	N.D	Valid	N.D	N.D	Valid	N.D	N.D
	Valid	N.D	N.D	Valid	N.D	N.D	Valid	N.D	N.D	Valid	N.D	N.D	Valid	N.D	N.D	Valid	N.D	N.D
	Valid	N.D	N.D	Valid	N.D	N.D	Valid	N.D	N.D	Valid	N.D	N.D	Valid	N.D	N.D	Valid	N.D	N.D
	Valid	N.D	N.D	Valid	N.D	N.D	Valid	N.D	N.D	Valid	N.D	N.D	Valid	N.D	N.D	Valid	N.D	N.D
	Valid	N.D	N.D	Valid	N.D	N.D	Valid	N.D	N.D	Valid	N.D	N.D	Valid	N.D	N.D	Valid	N.D	N.D
	Valid	N.D	N.D	Valid	N.D	N.D	Valid	N.D	N.D	Valid	N.D	N.D	Valid	N.D	N.D	Valid	N.D	N.D
	Valid	N.D	N.D	Valid	N.D	N.D	Valid	N.D	N.D	Valid	N.D	N.D	Valid	N.D	N.D	Valid	N.D	N.D
3 copies/ul	Valid	31.32	30.81	Valid	32.17	31.37	N/A			Valid	31.74	31.60	Valid	31.61	32.05	N/A		
	Valid	31.78	30.99	Valid	31.04	31.79				Valid	30.98	31.53	Valid	31.54	31.93			
	Valid	31.63	30.87	Valid	31.24	31.42				Valid	32.00	31.99	Valid	31.64	31.86			
	Valid	31.39	30.99	Valid	32.13	31.49				Valid	32.15	31.88	Valid	31.43	32.15			

	Valid	31.74	31.75	Valid	32.36	30.85				Valid	31.64	32.34	Valid	32.14	31.59			
	Valid	31.19	30.21	Valid	30.94	30.91				Valid	31.34	32.21	Valid	31.45	31.97			
	Valid	31.98	31.02	Valid	31.81	30.41				Valid	31.54	31.74	Valid	31.50	31.62			
	Valid	30.58	31.34	Valid	31.14	30.99				Valid	31.24	31.49	Valid	31.11	31.72			
	Valid	31.86	30.89	Valid	31.06	30.73				Valid	31.50	31.88	Valid	31.43	31.73			
	Valid	31.81	30.90	Valid	31.58	31.28				Valid	31.44	31.74	Valid	31.94	31.60			
	Valid	31.76	31.38	Valid	30.92	30.86				Valid	31.75	31.77	Valid	31.25	31.75			
	Valid	31.47	31.12	Valid	31.84	30.85				Valid	31.42	31.58	Valid	31.85	31.92			
	Valid	31.03	30.65	Valid	31.83	31.18				Valid	31.72	32.21	Valid	31.55	31.75			
	Valid	31.95	31.28	Valid	32.00	31.31				Valid	32.09	31.82	Valid	31.58	32.21			
	Valid	31.43	31.06	Valid	31.47	30.87				Valid	28.99	30.26	Valid	29.09	30.39			
	Valid	31.76	31.11	Valid	30.92	31.10				Valid	28.58	30.05	Valid	28.80	29.82			
	Valid	31.69	31.34	Valid	31.60	31.31				Valid	29.38	30.02	Valid	29.16	30.17			
	Valid	31.48	31.75	Valid	32.97	31.49				Valid	28.62	29.58	Valid	29.53	30.16			
	Valid	31.74	31.11	Valid	32.16	30.78				Valid	28.40	30.36	Valid	29.51	30.83			
	Valid	31.63	31.04	Valid	32.06	31.24				Valid	28.94	30.61	Valid	29.41	30.41			
	Valid	31.27	30.49	Valid	31.02	30.71				Valid	29.15	29.79	Valid	29.31	30.72			
	Valid	32.02	31.62	Valid	31.62	31.52	Valid	32.24	31.82	Valid	31.39	31.66	Valid	32.36	32.37	Valid	31.36	31.61
	Valid	32.23	31.55	Valid	31.55	30.93	Valid	32.67	31.43	Valid	32.02	31.46	Valid	33.11	32.63	Valid	31.14	31.69
	Valid	31.85	31.49	Valid	31.49	31.09	Valid	32.79	31.53	Valid	32.66	32.59	Valid	32.27	33.15	Valid	29.74	29.97
	Valid	32.14	31.93	Valid	31.93	31.56	Valid	33.82	31.97	Valid	32.32	33.14	Valid	32.36	32.73	Valid	30.40	31.08
	Valid	33.37	32.72	Valid	32.72	31.71	Valid	33.67	31.94	Valid	33.12	32.65	Valid	32.40	32.70	Valid	29.91	30.95
	Valid	32.72	36.09	Valid	36.09	32.47	Valid	33.02	31.23	Valid	32.94	32.95	Valid	32.03	32.96	Valid	30.18	30.82
	Valid	31.98	32.29	Valid	32.29	31.31	Valid	33.03	32.06	Valid	32.04	32.49	Valid	32.46	32.33	Valid	30.32	30.86
**2 copies/ ul**	Valid	32.37	32.12	Valid	32.13	31.19	Valid	33.72	31.63	Valid	31.65	31.81	Valid	32.13	32.93	Valid	30.53	31.06
	Valid	30.82	31.40	Valid	32.10	31.72	Valid	32.65	31.23	Valid	31.97	32.28	Valid	33.08	32.97	Valid	30.89	31.14
	Valid	32.30	31.78	Valid	32.21	31.38	Valid	34.72	31.78	Valid	32.33	32.54	Valid	32.26	32.84	Valid	30.60	30.65
	Valid	31.75	31.15	Valid	33.81	31.98	Valid	32.35	31.82	Valid	32.29	32.72	Valid	32.20	32.91	Valid	30.42	31.04
	Valid	32.25	31.84	Valid	32.62	31.74	Valid	32.48	31.28	Valid	33.57	32.59	Valid	32.26	32.47	Valid	30.38	30.89
	Valid	32.37	31.91	Valid	32.68	31.13	Valid	33.36	31.44	Valid	32.44	32.64	Valid	32.92	32.33	Valid	29.96	30.67
	Valid	32.52	31.19	Valid	32.58	31.16	Valid	33.66	31.30	Valid	32.54	32.77	Valid	32.69	31.94	Valid	30.17	30.65
	Valid	31.62	31.72	Valid	31.79	31.96	Valid	33.43	31.60	Valid	29.49	30.81	Valid	30.30	30.39	Valid	31.00	31.00

	Valid	32.24	31.58	Valid	31.65	31.64	Valid	33.11	31.64	Valid	29.68	30.34	Valid	28.94	29.83	Valid	29.89	30.57
	Valid	31.91	31.87	Valid	32.48	31.52	Valid	33.49	30.94	Valid	29.31	30.39	Valid	30.47	30.42	Valid	30.49	31.04
	Valid	32.53	31.93	Valid	31.73	31.67	Valid	32.57	31.59	Valid	29.33	31.12	Valid	29.94	30.38	Valid	30.75	31.21
	Valid	33.58	31.98	Valid	32.01	31.72	Valid	33.09	32.31	Valid	30.30	31.07	Valid	30.99	31.24	Valid	30.69	31.28
	Valid	32.11	31.73	Valid	32.02	31.45	Valid	32.79	32.62	Valid	29.25	30.74	Valid	30.55	31.37	Valid	30.53	31.01
	Valid	31.85	31.19	Valid	31.90	31.64	Valid	32.50	31.94	Valid	N.D	N.D	Valid	30.49	30.23	Valid	30.45	31.14

2. *ExiCycler*™ 384 thermo block (BIONEER), *Exicycler*™96 (BIONEER) , *Exicycler*™96 V4 Fast (BIONEER)

Sample	*Exicycler*™384									*Exicycler™96*								
	Sputum			Swab			Saliva			Sputum			Swab			Saliva		
	IPC	Pan-Sarbecovirus gene	SARS-CoV-2 gene	IPC	Pan-Sarbecovirus gene	SARS-CoV-2 gene	IPC	Pan-Sarbecovirus gene	SARS-CoV-2 gene	IPC	Pan-Sarbecovirus gene	SARS-CoV-2 gene	IPC	Pan-Sarbecovirus gene	SARS-CoV-2 gene	IPC	Pan-Sarbecovirus gene	SARS-CoV-2 gene
**NTC**	Valid	N.D	N.D	N/A			Valid	N.D	N.D	Valid	N.D	N.D	N/A			Valid	N.D	N.D
**PC**	Valid	22.04	22.01				Valid	22.01	22.07	Valid	23.2	23.03				Valid	21.79	23.03
**Neg**	Valid	N.D	N.D	Valid	Valid	N.D	Valid	N.D	N.D	Valid	N.D	N.D	Valid	N.D	N.D	Valid	N.D	N.D
	Valid	N.D	N.D	Valid	Valid	N.D	Valid	N.D	N.D	Valid	N.D	N.D	Valid	N.D	N.D	Valid	N.D	N.D
	Valid	N.D	N.D	Valid	Valid	N.D	Valid	N.D	N.D	Valid	N.D	N.D	Valid	N.D	N.D	Valid	N.D	N.D
	Valid	N.D	N.D	Valid	Valid	N.D	Valid	N.D	N.D	Valid	N.D	N.D	Valid	N.D	N.D	Valid	N.D	N.D
	Valid	N.D	N.D	Valid	Valid	N.D	Valid	N.D	N.D	Valid	N.D	N.D	Valid	N.D	N.D	Valid	N.D	N.D
	Valid	N.D	N.D	Valid	Valid	N.D	Valid	N.D	N.D	Valid	N.D	N.D	Valid	N.D	N.D	Valid	N.D	N.D
	Valid	N.D	N.D	Valid	Valid	N.D	Valid	N.D	N.D	Valid	N.D	N.D	Valid	N.D	N.D	Valid	N.D	N.D
	Valid	N.D	N.D	Valid	Valid	N.D	Valid	N.D	N.D	Valid	N.D	N.D	Valid	N.D	N.D	Valid	N.D	N.D
	Valid	N.D	N.D	Valid	Valid	N.D	Valid	N.D	N.D	Valid	N.D	N.D	Valid	N.D	N.D	Valid	N.D	N.D
	Valid	N.D	N.D	Valid	Valid	N.D	Valid	N.D	N.D	Valid	N.D	N.D	Valid	N.D	N.D	Valid	N.D	N.D
	Valid	N.D	N.D	Valid	Valid	N.D	Valid	N.D	N.D	Valid	N.D	N.D	Valid	N.D	N.D	Valid	N.D	N.D
	Valid	N.D	N.D	Valid	Valid	N.D	Valid	N.D	N.D	Valid	N.D	N.D	Valid	N.D	N.D	Valid	N.D	N.D
	Valid	N.D	N.D	Valid	Valid	N.D	Valid	N.D	N.D	Valid	N.D	N.D	Valid	N.D	N.D	Valid	N.D	N.D
	Valid	N.D	N.D	Valid	Valid	N.D	Valid	N.D	N.D	Valid	N.D	N.D	Valid	N.D	N.D	Valid	N.D	N.D
	Valid	N.D	N.D	Valid	Valid	N.D	Valid	N.D	N.D	Valid	N.D	N.D	Valid	N.D	N.D	Valid	N.D	N.D
	Valid	N.D	N.D	Valid	Valid	N.D	Valid	N.D	N.D	Valid	N.D	N.D	Valid	N.D	N.D	Valid	N.D	N.D
	Valid	N.D	N.D	Valid	Valid	N.D	Valid	N.D	N.D	Valid	N.D	N.D	Valid	N.D	N.D	Valid	N.D	N.D
	Valid	N.D	N.D	Valid	Valid	N.D	Valid	N.D	N.D	Valid	N.D	N.D	Valid	N.D	N.D	Valid	N.D	N.D

	Valid	N.D	N.D	Valid	Valid	N.D	Valid	N.D	N.D	Valid	N.D	N.D	Valid	N.D	N.D	Valid	N.D	N.D
	Valid	N.D	N.D	Valid	Valid	N.D	Valid	N.D	N.D	Valid	N.D	N.D	Valid	N.D	N.D	Valid	N.D	N.D
	Valid	N.D	N.D	Valid	Valid	N.D	Valid	N.D	N.D	Valid	N.D	N.D	Valid	N.D	N.D	Valid	N.D	N.D
	Valid	31.3	30.82	Valid	31.12	30.76				Valid	30.59	31	Valid	31.15	30.68			
	Valid	31.16	30.79	Valid	30.99	30.72				Valid	31.12	30.73	Valid	30.55	30.9			
	Valid	31.54	31.03	Valid	31.32	30.98				Valid	31.2	31.05	Valid	31.15	30.9			
	Valid	31.03	30.95	Valid	30.8	30.87				Valid	31.07	30.57	Valid	31.19	31.01			
	Valid	31.11	30.24	Valid	30.91	30.16				Valid	31.2	30.86	Valid	30.99	31.2			
	Valid	31.09	31.14	Valid	30.9	31.08				Valid	30.95	30.78	Valid	31.03	30.54			
	Valid	31.24	30.71	Valid	31.05	30.64				Valid	30.92	30.77	Valid	31.13	31.02			
	Valid	31.06	31.11	Valid	31.54	30.92				Valid	31.41	30.79	Valid	31.17	30.98			
	Valid	31.16	30.71	Valid	31.68	30.5				Valid	30.61	31.01	Valid	31.2	31.14			
	Valid	31.23	30.92	Valid	31.76	30.71				Valid	31.21	31.01	Valid	31.32	31.45			
**3 copies/ ul**	Valid	31.28	31.42	Valid	31.84	31.24		N/A		Valid	31.49	31.08	Valid	31.92	30.93		N/A	
	Valid	31.2	30.83	Valid	31.72	30.58				Valid	31.04	31.28	Valid	31.47	30.54			
	Valid	30.64	30.79	Valid	31.17	30.58				Valid	31.08	30.64	Valid	30.68	31			
	Valid	31.36	31.35	Valid	31.97	31.17				Valid	31.19	31.1	Valid	31.68	31.12			
	Valid	31.51	30.65	Valid	30.79	30.73				Valid	31.53	31.11	Valid	30.94	30.64			
	Valid	31.54	31.02	Valid	30.83	30.56				Valid	30.96	31.08	Valid	31.34	31.39			
	Valid	31.18	31.45	Valid	30.91	31.04				Valid	31.78	30.69	Valid	31.18	30.78			
	Valid	31.28	30.8	Valid	31.74	31.51				Valid	31.61	30.92	Valid	31.12	30.74			
	Valid	31.45	30.52	Valid	31.03	30.75				Valid	31.45	31.04	Valid	31.04	30.97			
	Valid	31.39	30.71	Valid	31.24	31.05				Valid	31.44	31.16	Valid	31.23	30.75			
	Valid	31.61	30.73	Valid	31.43	30.92				Valid	31.58	31.18	Valid	31.28	31.07			
	Valid	32.4	32.63	Valid	32.21	32.55	Valid	31.2	29.87	Valid	31.06	31.04	Valid	31.06	30.93	Valid	31.41	30.51
	Valid	33.3	33.65	Valid	33.06	33.5	Valid	31.98	29.71	Valid	31.4	31.01	Valid	30.82	30.53	Valid	31.13	30.93
	Valid	32.77	32.7	Valid	32.51	32.62	Valid	31.02	29.43	Valid	30.81	30.96	Valid	31.24	31.28	Valid	30.31	31.13
**2 copies/ ul**	Valid	32.14	32.55	Valid	31.92	32.47	Valid	32.86	29.98	Valid	31.61	30.59	Valid	31.09	30.6	Valid	32.33	30.89
	Valid	32.5	32.07	Valid	32.29	31.99	Valid	31.23	30.57	Valid	31.28	30.82	Valid	30.84	30.65	Valid	31.15	30.98
	Valid	32.39	34.48	Valid	32.14	34.31	Valid	31.94	30.09	Valid	31.33	30.95	Valid	30.93	30.87	Valid	31.45	31.43
	Valid	32.23	32.04	Valid	32.04	31.97	Valid	32.06	30.73	Valid	31.32	31.09	Valid	31.13	30.66	Valid	30.59	31.02
	Valid	32.72	32.21	Valid	33.27	32.06	Valid	32.19	30.21	Valid	31.12	31.03	Valid	30.9	31.08	Valid	32.04	31.01

	Valid	33.42	32.32	Valid	34.08	32.15	Valid	30.88	30.84	Valid	30.89	30.63	Valid	31.54	31.21	Valid	29.64	28.6
	Valid	32.87	32.5	Valid	33.66	32.26	Valid	31.31	30.66	Valid	31.21	31.36	Valid	31.6	30.91	Valid	30.82	31.32
	Valid	32.51	32.8	Valid	33.15	32.59	Valid	31.2	30.16	Valid	31.14	30.7	Valid	31.64	30.96	Valid	31.12	31.19
	Valid	32.13	32.66	Valid	32.63	32.44	Valid	32.67	30.84	Valid	30.92	30.75	Valid	30.75	31.05	Valid	31.35	31.44
	Valid	35.25	33.02	Valid	36.7	32.66	Valid	32.36	30.8	Valid	31.01	30.98	Valid	31.47	31.28	Valid	31.31	31.36
	Valid	32.42	33.06	Valid	33.06	32.71	Valid	31.33	30.46	Valid	31.19	30.76	Valid	30.95	31.19	Valid	30.95	30.81
	Valid	32.68	27.42	Valid	33.53	32.77	Valid	31.36	30.91	Valid	31.25	30.98	Valid	31.16	31.01	Valid	31.76	31.28
	Valid	33.54	27.25	Valid	32.59	32.07	Valid	32.37	30.72	Valid	30.71	31.03	Valid	31.28	31.19	Valid	31.61	31.65
	Valid	32.15	27.32	Valid	31.96	31.77	Valid	32.34	30.1	Valid	31.25	31	Valid	31.25	31.44	Valid	30.46	30.83
	Valid	32.55	27.1	Valid	31.94	31.62	Valid	33.8	33.8	Valid	31.66	N.D	Valid	31.64	30.92	Valid	31.17	30.97
	Valid	32.18	27.33	Valid	32.25	31.9	Valid	N.D	32.7	Valid	31.16	31.27	Valid	31.3	30.53	Valid	30.44	31.29
	Valid	33.21	27.03	Valid	32.6	32.16	Valid	35.69	33.89	Valid	31.04	30.63	Valid	30.73	30.99	Valid	32.3	32.05
	Valid	32.76	26.74	Valid	32.08	31.89	Valid	35.21	33.98	Valid	31.23	31.09	Valid	N.D	31.11	Valid	32.1	31.23

Sample	*Exicycler*^{TM}96 V4 FAST								
	Sputum			Swab			Saliva		
	IPC	Pan-Sarbecovirus gene	SARS-CoV-2 gene	IPC	Pan-Sarbecovirus gene	SARS-CoV-2 gene	IPC	Pan-Sarbecovirus gene	SARS-CoV-2 gene
NTC	Valid	N.D	N.D	N/A			Valid	N.D	N.D
PC	Valid	22.75	22.44				Valid	21.87	21.11
Neg	Valid	N.D	N.D	Valid	N.D	N.D	Valid	N.D	N.D
	Valid	N.D	N.D	Valid	N.D	N.D	Valid	N.D	N.D
	Valid	N.D	N.D	Valid	N.D	N.D	Valid	N.D	N.D
	Valid	N.D	N.D	Valid	N.D	N.D	Valid	N.D	N.D
	Valid	N.D	N.D	Valid	N.D	N.D	Valid	N.D	N.D
	Valid	N.D	N.D	Valid	N.D	N.D	Valid	N.D	N.D
	Valid	N.D	N.D	Valid	N.D	N.D	Valid	N.D	N.D
	Valid	N.D	N.D	Valid	N.D	N.D	Valid	N.D	N.D
	Valid	N.D	N.D	Valid	N.D	N.D	Valid	N.D	N.D
	Valid	N.D	N.D	Valid	N.D	N.D	Valid	N.D	N.D
	Valid	N.D	N.D	Valid	N.D	N.D	Valid	N.D	N.D
	Valid	N.D	N.D	Valid	N.D	N.D	Valid	N.D	N.D

	Valid	N.D	N.D	Valid	N.D	N.D	Valid	N.D	N.D
	Valid	N.D	N.D	Valid	N.D	N.D	Valid	N.D	N.D
	Valid	N.D	N.D	Valid	N.D	N.D	Valid	N.D	N.D
	Valid	N.D	N.D	Valid	N.D	N.D	Valid	N.D	N.D
	Valid	N.D	N.D	Valid	N.D	N.D	Valid	N.D	N.D
	Valid	N.D	N.D	Valid	N.D	N.D	Valid	N.D	N.D
	Valid	N.D	N.D	Valid	N.D	N.D	Valid	N.D	N.D
	Valid	N.D	N.D	Valid	N.D	N.D	Valid	N.D	N.D
	Valid	N.D	N.D	Valid	N.D	N.D	Valid	N.D	N.D
**3 copies/ul**	Valid	31.43	31.07	Valid	31.32	31	N/A		
	Valid	31.57	30.94	Valid	31.15	30.72			
	Valid	30.76	31.11	Valid	30.93	31.03			
	Valid	30.64	30.93	Valid	31.02	30.63			
	Valid	30.75	30.91	Valid	31.72	30.68			
	Valid	30.89	30.6	Valid	31.35	30.95			
	Valid	31.25	30.49	Valid	31.46	31.13			
	Valid	31.41	31.27	Valid	31.06	31.24			
	Valid	31.55	31.15	Valid	31.28	31.22			
	Valid	30.74	31.31	Valid	31	30.88			
	Valid	30.62	31.14	Valid	31.35	30.99			
	Valid	30.73	31.13	Valid	31.44	31.23			
	Valid	30.87	30.85	Valid	31.07	31.23			
	Valid	31.24	30.73	Valid	31.13	31.34			
	Valid	31.99	31.33	Valid	31.97	31.26			
	Valid	32.31	31.12	Valid	31.47	30.91			
	Valid	31.95	31.35	Valid	32.07	31.36			
	Valid	31.02	31.19	Valid	32.04	30.93			
	Valid	31.22	31.1	Valid	32.3	31.05			
	Valid	31.98	31.03	Valid	32.11	31.28			
	Valid	31.68	30.84	Valid	32.18	31.4			
**2 copies/ul**	Valid	30.83	30.56	Valid	31.08	31.05	Valid	30.79	30.88
	Valid	31.54	31.01	Valid	31.3	31.03	Valid	30.54	31.03
	Valid	30.93	30.73	Valid	31.02	30.62	Valid	31.64	31.04

	Valid	31.61	30.96	Valid	31.37	30.72	Valid	31.39	31.64
	Valid	30.62	30.68	Valid	31.46	31.03	Valid	32.23	31.03
	Valid	31.4	30.8	Valid	31.08	31.04	Valid	32.04	30.98
	Valid	31.16	31.06	Valid	31.15	31.14	Valid	31.11	31.74
	Valid	30.81	30.8	Valid	31.31	31.31	Valid	32.05	32.14
	Valid	31.52	31.2	Valid	31.26	31.54	Valid	31.46	31.01
	Valid	N.D	31	Valid	32.04	31.37	Valid	31.41	31.41
	Valid	31.58	31.17	Valid	31.56	31.39	Valid	31.25	31.54
	Valid	30.6	30.95	Valid	31.26	30.88	Valid	32.33	31.81
	Valid	31.38	31.05	Valid	31.65	31.2	Valid	31.93	31.59
	Valid	31.15	31.25	Valid	30.52	31.24	Valid	31.87	32
	Valid	31.38	30.84	Valid	31.66	31.31	Valid	31.63	31.41
	Valid	32.4	31.3	Valid	31.68	31.4	Valid	32.26	32.03
	Valid	31.46	31.12	Valid	31.4	30.96	Valid	29.13	30.82
	Valid	32.52	31.2	Valid	32.39	31.08	Valid	31.25	31.37
	Valid	31.2	30.99	Valid	31.79	31.29	Valid	31.63	31.72
	Valid	31.8	31.02	Valid	31.68	31.29	Valid	32.06	31.17
	Valid	31.52	31.28	Valid	31.68	31.49	Valid	32.95	31.59

Cross-reactivity

1. *AccuPower*® COVID-19 Multiplex Real-Time RT-PCR Kit (NCVM)

No	Cross reactivity sample	Sputum			Swab			Saliva		
		Pan-Sarbecovirus gene	SARS-CoV-2 gene	Pass/Fail	Pan-Sarbecovirus gene	SARS-CoV-2 gene	Pass/Fail	Pan-Sarbecovirus gene	SARS-CoV-2 gene	Pass/Fail
1	HCoV-229E	ND	ND	Pass	ND	ND	Pass	ND	ND	Pass
2	HCoV-OC43	ND	ND	Pass	ND	ND	Pass	ND	ND	Pass
3	HCoV-HKU1	ND	ND	Pass	ND	ND	Pass	ND	ND	Pass
4	HCoV-NL63	ND	ND	Pass	ND	ND	Pass	ND	ND	Pass
5	MERS-coronavirus	ND	ND	Pass	ND	ND	Pass	ND	ND	Pass
6	Adenovirus type 3 (type B)	ND	ND	Pass	ND	ND	Pass	ND	ND	Pass
7	Adenovirus (Ad. 71)	ND	ND	Pass	ND	ND	Pass	ND	ND	Pass
8	Human Metapneumovirus (hMPV)	ND	ND	Pass	ND	ND	Pass	ND	ND	Pass
9	Parainfluenza virus 1	ND	ND	Pass	ND	ND	Pass	ND	ND	Pass
10	Parainfluenza virus 2	ND	ND	Pass	ND	ND	Pass	ND	ND	Pass
11	Parainfluenza virus 3	ND	ND	Pass	ND	ND	Pass	ND	ND	Pass
12	Parainfluenza virus 4	ND	ND	Pass	ND	ND	Pass	ND	ND	Pass
13	Influenza A H1N1	ND	ND	Pass	ND	ND	Pass	ND	ND	Pass
14	Influenza A H3N2	ND	ND	Pass	ND	ND	Pass	ND	ND	Pass
15	Influenza B	ND	ND	Pass	ND	ND	Pass	ND	ND	Pass
16	Enterovirus 71	ND	ND	Pass	ND	ND	Pass	ND	ND	Pass
17	Respiratory syncytial virus A	ND	ND	Pass	ND	ND	Pass	ND	ND	Pass
18	Respiratory syncytial virus B	ND	ND	Pass	ND	ND	Pass	ND	ND	Pass
19	Rhinovirus 14 (type B)	ND	ND	Pass	ND	ND	Pass	ND	ND	Pass
20	Chlamydia pneumonia	ND	ND	Pass	ND	ND	Pass	ND	ND	Pass
21	Haemophilus influenzae	ND	ND	Pass	ND	ND	Pass	ND	ND	Pass
22	Legionella pneumophila	ND	ND	Pass	ND	ND	Pass	ND	ND	Pass
23	Mycobacterium tuberculosis	ND	ND	Pass	ND	ND	Pass	ND	ND	Pass
24	Streptococcus pneumonia	ND	ND	Pass	ND	ND	Pass	ND	ND	Pass
25	Streptococcus pyrogenes	ND	ND	Pass	ND	ND	Pass	ND	ND	Pass
26	Bordetella pertussis	ND	ND	Pass	ND	ND	Pass	ND	ND	Pass
27	Mycoplasma pneumoniae	ND	ND	Pass	ND	ND	Pass	ND	ND	Pass
28	Pooled human nasal wash	ND	ND	Pass	ND	ND	Pass	ND	ND	Pass
29	NATrol Coronavirus-SARS Stock	27.72	ND	Pass	27.50	ND	Pass	27.81	ND	Pass

2. *AccuPower*® SARS-CoV-2 Multiplex Real-Time RT-PCR Kit (SCVM)

No	Cross reactivity sample	Sputum			Swab					
		Pan-Sarbecovirus gene	SARS-CoV-2 gene	Pass/Fail	Pan-Sarbecovirus gene	SARS-CoV-2 gene	Pass/Fail	Pan-Sarbecovirus gene	SARS-CoV-2 gene	Pass/Fail
1	HCoV-229E	ND	ND	Pass	ND	ND	Pass	ND	ND	Pass
2	HCoV-OC43	ND	ND	Pass	ND	ND	Pass	ND	ND	Pass
3	HCoV-HKU1	ND	ND	Pass	ND	ND	Pass	ND	ND	Pass
4	HCoV-NL63	ND	ND	Pass	ND	ND	Pass	ND	ND	Pass
5	MERS-coronavirus	ND	ND	Pass	ND	ND	Pass	ND	ND	Pass
6	Adenovirus type 3 (type B)	ND	ND	Pass	ND	ND	Pass	ND	ND	Pass
7	Adenovirus (Ad. 71)	ND	ND	Pass	ND	ND	Pass	ND	ND	Pass
8	Human Metapneumovirus (hMPV)	ND	ND	Pass	ND	ND	Pass	ND	ND	Pass
9	Parainfluenza virus 1	ND	ND	Pass	ND	ND	Pass	ND	ND	Pass
10	Parainfluenza virus 2	ND	ND	Pass	ND	ND	Pass	ND	ND	Pass
11	Parainfluenza virus 3	ND	ND	Pass	ND	ND	Pass	ND	ND	Pass
12	Parainfluenza virus 4	ND	ND	Pass	ND	ND	Pass	ND	ND	Pass
13	Influenza A H1N1	ND	ND	Pass	ND	ND	Pass	ND	ND	Pass
14	Influenza A H3N2	ND	ND	Pass	ND	ND	Pass	ND	ND	Pass
15	Influenza B	ND	ND	Pass	ND	ND	Pass	ND	ND	Pass
16	Enterovirus 71	ND	ND	Pass	ND	ND	Pass	ND	ND	Pass
17	Respiratory syncytial virus A	ND	ND	Pass	ND	ND	Pass	ND	ND	Pass
18	Respiratory syncytial virus B	ND	ND	Pass	ND	ND	Pass	ND	ND	Pass
19	Rhinovirus 14 (type B)	ND	ND	Pass	ND	ND	Pass	ND	ND	Pass
20	Chlamydia pneumonia	ND	ND	Pass	ND	ND	Pass	ND	ND	Pass
21	Haemophilus influenzae	ND	ND	Pass	ND	ND	Pass	ND	ND	Pass
22	Legionella pneumophila	ND	ND	Pass	ND	ND	Pass	ND	ND	Pass
23	Mycobacterium tuberculosis	ND	ND	Pass	ND	ND	Pass	ND	ND	Pass
24	Streptococcus pneumonia	ND	ND	Pass	ND	ND	Pass	ND	ND	Pass
25	Streptococcus pyrogenes	ND	ND	Pass	ND	ND	Pass	ND	ND	Pass
26	Bordetella pertussis	ND	ND	Pass	ND	ND	Pass	ND	ND	Pass
27	Mycoplasma pneumoniae	ND	ND	Pass	ND	ND	Pass	ND	ND	Pass
28	Pooled human nasal wash	ND	ND	Pass	ND	ND	Pass	ND	ND	Pass
29	NATrol Coronavirus-SARS Stock	22.43	ND	Pass	22.48	ND	Pass	22.84	ND	Pass
30	Enterovirus 70	ND	ND	Pass	ND	ND	Pass	ND	ND	Pass
31	Coxsackievrus B5	ND	ND	Pass	ND	ND	Pass	ND	ND	Pass
32	Echovirus 25	ND	ND	Pass	ND	ND	Pass	ND	ND	Pass
33	Human Parachovirus 3	ND	ND	Pass	ND	ND	Pass	ND	ND	Pass
34	Mycobacterium fortuitum	ND	ND	Pass	ND	ND	Pass	ND	ND	Pass
35	Mycobacterium intracell	ND	ND	Pass	ND	ND	Pass	ND	ND	Pass
36	Mycobacterium gordonae	ND	ND	Pass	ND	ND	Pass	ND	ND	Pass
37	Mycobacterium chelonae	ND	ND	Pass	ND	ND	Pass	ND	ND	Pass
38	Pneumocystis jirovecii(PJP)	ND	ND	Pass	ND	ND	Pass	ND	ND	Pass

S1 Table. Precision evaluation results for the *AccPower*® kits.

1. *AccuPower*® COVID-19 Multiplex Real-Time RT-PCR Kit (NCVM)

Matrix		Sample_Sputum															
Concentration		Negative				LoD (120 copies/㎖)				LoDX3 (360 copies/㎖)				LoDX6 (720 copies/㎖)			
Run		Run 1		Run 2		Run 1		Run 2		Run 1		Run 2		Run 1		Run 2	
Target	Day	Rep 1	Rep 2	Rep 1	Rep 2	Rep 1	Rep 2	Rep 1	Rep 2	Rep 1	Rep 2	Rep 1	Rep 2	Rep 1	Rep 2	Rep 1	Rep 2
Pan-Sarbecovirus gene	1	N.D	N.D	N.D	N.D	39.37	37.34	38.22	39.05	36.68	37.54	37.25	37.28	36.32	36.32	36.18	36.56
	2	N.D	N.D	N.D	N.D	36.88	38.84	39	35.53	37.59	37.18	36.51	39.45	35.93	36.10	36.16	36.16
	3	N.D	N.D	N.D	N.D	37.21	39.59	39.21	39.13	37.58	37.09	37.40	36.78	36.91	36.27	35.55	35.36
	4	N.D	N.D	N.D	N.D	38.65	37.18	38.89	38.75	38.00	37.04	37.58	36.80	36.42	36.47	35.34	34.31
	5	N.D	N.D	N.D	N.D	35.67	38.82	39.37	36.21	37.23	37.33	36.78	38.37	37.13	36.52	36.98	37.41
SARS-CoV-2 gene	1	N.D	N.D	N.D	N.D	38.01	37.11	36.24	36.88	35.08	35.29	33.56	34.06	33.86	34.24	33.69	34.22
	2	N.D	N.D	N.D	N.D	37.54	38.10	37.31	37.70	35.33	36.39	35.18	35.45	35.28	35.80	34.03	34.39
	3	N.D	N.D	N.D	N.D	38.60	37.08	38.07	38.21	35.48	34.88	35.70	35.42	34.53	34.41	34.54	34.17
	4	N.D	N.D	N.D	N.D	37.89	38.42	38.38	37.07	35.50	35.09	34.59	35.21	33.90	34.17	33.98	33.56
	5	N.D	N.D	N.D	N.D	38.44	36.34	38.71	35.16	35.84	35.11	36.68	35.59	34.70	34.57	36.64	35.68

Matrix		Sample_Swab															
Concentration		Negative				LoD (120 copies/㎖)				LoDX3 (360 copies/㎖)				LoDX6 (720 copies/㎖)			
Run		Run 1		Run 2		Run 1		Run 2		Run 1		Run 2		Run 1		Run 2	
Target	Day	Rep 1	Rep 2	Rep 1	Rep 2	Rep 1	Rep 2	Rep 1	Rep 2	Rep 1	Rep 2	Rep 1	Rep 2	Rep 1	Rep 2	Rep 1	Rep 2
Pan-Sarbecovirus gene	1	N.D	N.D	N.D	N.D	39.41	37.58	39.18	39.64	36.30	36.99	36.75	37.07	36.07	36.36	36.04	36.49
	2	N.D	N.D	N.D	N.D	37.49	38.00	39.12	38.33	36.80	37.51	36.61	36.74	36.06	35.78	35.76	35.44
	3	N.D	N.D	N.D	N.D	39.74	38.97	39.13	39.45	36.88	37.12	36.47	36.13	36.21	36.64	34.88	35.34
	4	N.D	N.D	N.D	N.D	36.39	39.27	39.53	38.83	38.05	38.11	35.35	36.66	36.65	37.09	34.69	35.07
	5	N.D	N.D	N.D	N.D	38.52	38.51	39.29	38.97	37.85	36.91	37.79	37.81	36.30	36.35	37.82	41.86
SARS-CoV-2 gene	1	N.D	N.D	N.D	N.D	38.93	36.75	37.42	38.94	34.31	35.33	35.33	35.37	34.72	34.56	33.65	34.46
	2	N.D	N.D	N.D	N.D	37.82	38.78	35.28	38.78	36.36	36.54	35.26	35.33	35.33	35.24	34.22	34.10
	3	N.D	N.D	N.D	N.D	38	38.95	38.72	38.71	35.80	35.93	35.51	35.14	33.93	34.67	34.15	34.37
	4	N.D	N.D	N.D	N.D	38.87	37.99	37.56	37.25	35.14	35.75	34.66	34.77	34.32	34.56	34.02	34.14
	5	N.D	N.D	N.D	N.D	35.19	37.11	37.97	38.6	35.73	35.29	35.63	35.78	34.09	34.34	34.26	34.86

Matrix	Sample_Saliva

Concentration		Negative				LoD (120 copies/㎖)				LoDX3 (360 copies/㎖)				LoDX6 (720 copies/㎖)			
Run		Run 1		Run 2		Run 1		Run 2		Run 1		Run 2		Run 1		Run 2	
Target	Day	Rep 1	Rep 2	Rep 1	Rep 2	Rep 1	Rep 2	Rep 1	Rep 2	Rep 1	Rep 2	Rep 1	Rep 2	Rep 1	Rep 2	Rep 1	Rep 2
Pan-Sarbecovirus gene	1	N.D	N.D	N.D	N.D	38.35	39.39	38.68	38.87	37.55	37.94	37.51	38.28	36.40	36.44	37.05	37.32
	2	N.D	N.D	N.D	N.D	39.61	39.25	39.23	38.81	38.11	37.57	39.22	37.08	36.75	37.36	36.88	36.80
	3	N.D	N.D	N.D	N.D	39.89	38.71	38.28	39.57	39.10	37.92	37.91	37.67	37.50	37.19	36.96	37.40
	4	N.D	N.D	N.D	N.D	39.18	38.47	38.84	37.97	38.05	38.04	37.73	37.90	37.04	37.11	36.55	36.79
	5	N.D	N.D	N.D	N.D	39.52	37.29	39.05	37.93	37.29	37.13	37.38	37.48	37.09	37.00	36.94	37.15
SARS-CoV-2 gene	1	N.D	N.D	N.D	N.D	37.57	37.94	38.32	38.15	37.75	38.12	37.85	35.55	36.16	36.81	36.36	36.19
	2	N.D	N.D	N.D	N.D	38.77	37.11	38.30	38.39	37.58	37.94	35.55	37.93	37.25	37.11	36.75	36.76
	3	N.D	N.D	N.D	N.D	38.86	38.55	38.80	38.60	38.19	38.40	38.02	37.80	36.67	36.64	36.37	36.31
	4	N.D	N.D	N.D	N.D	38.97	37.72	38.89	38.04	37.39	38.69	37.78	37.15	36.69	36.94	36.55	36.52
	5	N.D	N.D	N.D	N.D	38.64	37.21	38.68	38.68	37.55	35.80	37.70	38.50	36.90	36.50	36.67	37.47

2. *AccuPower*® SARS-CoV-2 Multiplex Real-Time RT-PCR Kit (SCVM)

Target		**Sputum**																			
Concentration		Negative				LoD(2 copies/㎕)				LoDX3(6 copies/㎕)				LoDX6(12 copies/㎕)				LoDX300(600 copies/㎕)			
Run		Run 1		Run 2		Run 1		Run 2		Run 1		Run 2		Run 1		Run 2		Run 1		Run 2	
Target	Day	Rep 1	Rep 2	Rep 1	Rep 2	Rep 1	Rep 2	Rep 1	Rep 2	Rep 1	Rep 2	Rep 1	Rep 2	Rep 1	Rep 2	Rep 1	Rep 2	Rep 1	Rep 2	Rep 1	Rep 2
Pan-Sarbecovirus gene	1	N.D	N.D	N.D	N.D	32.30	32.21	32.73	31.48	31.23	31.01	30.28	30.61	30.05	30.41	29.40	29.42	24.23	24.19	23.65	23.68
	2	N.D	N.D	N.D	N.D	32.64	32.22	32.29	32.25	30.33	30.60	31.12	31.29	29.19	29.98	30.19	30.19	23.72	23.75	24.46	24.55
	3	N.D	N.D	N.D	N.D	32.24	32.88	32.21	33.85	31.23	31.20	31.30	30.76	29.87	29.96	29.96	29.72	24.20	24.16	23.99	24.09
	4	N.D	N.D	N.D	N.D	31.56	31.72	32.02	31.95	31.00	30.95	30.72	30.91	29.57	29.63	29.81	29.79	23.98	23.94	24.12	24.20
	5	N.D	N.D	N.D	N.D	31.66	32.23	32.28	32.12	31.07	30.81	30.40	30.12	29.85	30.23	29.09	29.41	24.07	24.04	23.48	23.58
SARS-CoV-2 gene	1	N.D	N.D	N.D	N.D	31.19	31.12	31.31	31.22	30.68	30.49	30.58	30.66	29.56	29.92	29.46	29.47	24.08	24.03	23.98	24.03
	2	N.D	N.D	N.D	N.D	31.28	31.45	31.58	31.71	30.15	30.19	31.08	30.80	28.66	29.29	30.11	29.95	23.34	23.29	24.20	24.24
	3	N.D	N.D	N.D	N.D	31.68	31.14	30.80	31.20	31.33	31.26	30.94	30.96	30.22	30.00	29.71	29.57	24.51	24.52	23.99	24.09
	4	N.D	N.D	N.D	N.D	31.49	31.40	30.98	31.82	31.10	31.04	30.69	30.41	30.01	29.70	29.71	29.52	24.28	24.29	23.85	23.90
	5	N.D	N.D	N.D	N.D	31.31	31.11	31.36	31.00	30.44	30.28	29.69	29.82	29.33	29.64	28.84	28.87	23.86	23.79	23.05	23.10

Target		Swab																			
Concentration		Negative				LoD(2 copies/㎕)				LoDX3(6 copies/㎕)				LoDX6(12 copies/㎕)				LoDX300(600 copies/㎕)			
Run		Run 1		Run 2		Run 1		Run 2		Run 1		Run 2		Run 1		Run 2		Run 1		Run 2	
Target	Day	Rep 1	Rep 2	Rep 1	Rep 2	Rep 1	Rep 2	Rep 1	Rep 2	Rep 1	Rep 2	Rep 1	Rep 2	Rep 1	Rep 2	Rep 1	Rep 2	Rep 1	Rep 2	Rep 1	Rep 2
Pan-Sarbecovirus gene	1	N.D	N.D	N.D	N.D	32.32	31.96	31.45	32.03	31.07	31.43	30.30	30.27	29.71	31.05	29.44	29.86	24.36	24.4	24.46	24.12
	2	N.D	N.D	N.D	N.D	32.02	33.92	31.67	32.16	30.61	31.40	30.87	30.81	29.49	30.38	29.66	29.79	24.01	24.44	24.14	24.46
	3	N.D	N.D	N.D	N.D	32.00	32.18	32.21	31.97	30.60	31.54	30.46	30.57	30.22	30.28	29.70	29.60	24.82	25.27	23.82	23.93
	4	N.D	N.D	N.D	N.D	31.88	32.08	32.18	32.05	30.33	31.27	30.47	30.43	29.99	30.04	29.31	29.4	24.53	25.03	23.81	24.14
	5	N.D	N.D	N.D	N.D	31.91	32.16	32.29	32.27	30.89	31.26	29.98	29.99	29.5	30.83	29.00	29.02	24.21	24.24	22.86	23.21
SARS-CoV-2 gene	1	N.D	N.D	N.D	N.D	31.50	30.98	31.15	30.94	30.5	30.58	30.51	30.37	29.63	30.17	29.69	29.81	24.06	23.97	24.73	24.35
	2	N.D	N.D	N.D	N.D	31.65	31.56	31.21	31.96	30.01	30.36	30.39	30.55	28.92	29.11	29.54	29.41	23.53	23.70	23.96	24.08
	3	N.D	N.D	N.D	N.D	31.35	31.48	31.15	31.11	30.75	31.13	30.20	30.40	30.14	30.48	29.50	29.58	25.18	25.36	23.93	23.97
	4	N.D	N.D	N.D	N.D	31.17	31.45	31.15	31.44	30.49	30.89	30.06	30.19	29.90	30.25	29.18	29.07	24.95	25.13	23.61	23.73
	5	N.D	N.D	N.D	N.D	31.22	31.05	31.32	31.19	30.28	30.35	29.21	29.64	29.39	29.94	28.56	28.43	23.85	23.74	22.35	22.55

Target		**Saliva**															
Concentration		Negative				LoD(2 copies/㎕)				LoDX3(6 copies/㎕)				LoDX6(12 copies/㎕)			
Run		Run 1		Run 2		Run 1		Run 2		Run 1		Run 2		Run 1		Run 2	
Target	Day	Rep 1	Rep 2	Rep 1	Rep 2	Rep 1	Rep 2	Rep 1	Rep 2	Rep 1	Rep 2	Rep 1	Rep 2	Rep 1	Rep 2	Rep 1	Rep 2

Pan-Sarbecovirus gene	1	N.D	N.D	N.D	N.D	32.33	30.53	32.03	31.21	29.45	30.52	30.42	29.93	29.31	28.43	28.79	28.82
	2	N.D	N.D	N.D	N.D	30.24	30.86	31.59	31.48	30.62	31.28	29.66	29.24	28.98	29.17	29.05	29.20
	3	N.D	N.D	N.D	N.D	30.78	30.80	32.44	32.16	29.88	29.75	30.24	30.04	28.50	29.03	28.59	29.31
	4	N.D	N.D	N.D	N.D	31.13	30.64	33.61	31.38	30.67	30.85	30.09	29.66	28.66	29.01	29.24	28.44
	5	N.D	N.D	N.D	N.D	30.91	31.13	32.60	31.50	29.99	30.20	30.13	30.18	28.64	28.71	28.91	28.82
SARS-CoV-2 gene	1	N.D	N.D	N.D	N.D	30.89	30.24	31.93	31.60	30.67	30.76	29.61	30.52	29.13	28.84	28.66	28.80
	2	N.D	N.D	N.D	N.D	30.76	30.71	32.15	31.32	30.42	30.55	30.04	29.67	29.21	29.33	28.93	29.02
	3	N.D	N.D	N.D	N.D	30.27	31.71	31.82	31.20	29.95	29.80	30.39	30.09	29.11	29.08	28.63	28.97
	4	N.D	N.D	N.D	N.D	32.30	30.87	31.69	30.61	30.21	30.51	29.92	29.73	29.42	29.00	29.20	28.94
	5	N.D	N.D	N.D	N.D	31.05	30.21	32.27	31.44	30.00	30.60	30.16	29.68	29.13	29.20	29.12	29.25
