# Supplementary material for: Development and Evaluation of AccuPower COVID-19 Multiplex Real-Time RT-PCR Kit and AccuPower SARS-CoV-2 Multiplex Real-Time RT-PCR Kit for SARS-CoV-2 Detection in Sputum, NPS/OPS, Saliva and Pooled Samples
Source: PLoS One. 2022 Feb 10;17(2):e0263341. doi: 10.1371/journal.pone.0263341 (PMC8830688; doi:10.1371/journal.pone.0263341)
Supplement: Attachment — Submitted filename: Response to Reviewers.docx [file pone.0263341.s011.docx]

**Responding to Reviewers**

Thank you for reviewing the manuscript. We respond to all of your comments as follows.

**< Journal requirements >**

1. **Please ensure that your manuscript meets PLOS ONE's style requirements, including those for file naming. The PLOS ONE style templates can be found at**

<https://journals.plos.org/plosone/s/file?id=wjVg/PLOSOne_formatting_sample_main_body.pdf>  **and**

<https://journals.plos.org/plosone/s/file?id=ba62/PLOSOne_formatting_sample_title_authors_affiliations.pdf>

🡪 As you commented, we uploaded files labeled ‘Response to Reviewers’, ‘Revised Manuscript with Track Changes’ and ‘Manuscript’. Uploaded files were written in PLOS ONE style templates.

2. **Please remove any funding-related text from the manuscript and let us know how you would like to update your Funding Statement.**

**🡪** As you commented, we removed funding-related text. (page 27)

3. **Please state what role the funders took in the study.  If the funders had no role, please state: "The funders had no role in study design, data collection and analysis, decision to publish, or preparation of the manuscript." If this statement is not correct you must amend it as needed. Please include this amended Role of Funder statement in your cover letter; we will change the online submission form on your behalf.”**

🡪 The funders had no role in study design, data collection and analysis, decision to publish, or preparation of the manuscript.

4. **Please include your full ethics statement in the ‘Methods’ section of your manuscript file. In your statement, please include the full name of the IRB or ethics committee who approved or waived your study, as well as whether or not you obtained informed written or verbal consent.**

**🡪** As you commented, the ethics statement has been added to the 'Method_Ethics Statement and Sample Collection' section (page 8).

**Ethics Statement and Sample Collection**

Authors confirm that this paper has complied with PLOS policies on research ethics, as stated in submission guide. Since all identification information of samples was anonymized by replacing it with the unique identification code of the institution, researchers have performed the clinical performance test without risks of identifying or tracing personal information with the sample. The protocol of the study was approved by the Institutional Review Board (IRB) of Myongji Hospital and IRB of Kangwon National University Hospital. Since samples were residual sample with guaranteed anonymity and there was no need to collect additional samples for this study, there was no risk to research subjects in accordance with ‘Bioethics and Safety Act’ and Article 13 of ‘Enforcement Rules of the In Vitro Diagnostic Medical Devices Act’. Hence, IRBs confirmed that this study does not need subject consent. This study was conducted in accordance with the principles of the Declaration of Helsinki, 1964.

Myongji Hospital Institutional Review Board (IRB) approved the use of surplus clinical samples for the NPS/OPS specimen-pooling (IRB No.: MJH 2020-12-028) and clinical performance evaluation (IRB No.: MJH 2020-12-029) tests. Kangwon National University Hospital IRB approved the use of saliva and NPS/OPS samples for the clinical performance testing of the *AccuPower*^®^ kits (IRB No.: KNUH-2021-03-014), which were either surplus samples or de-identified patient samples drawn after informed consent (Fig 1). RNA in the NPS/OPS and sputum samples was extracted using the *ExiPrep*™48 Viral DNA/RNA Kit (BIONEER, Korea) with the *ExiPrep*™48 Dx instrument and the RNA of saliva samples was extracted using the *ExiPrep*™48 Fast Viral RNA Kit (BIONEER, Korea) with the *ExiPrep*™48 Dx instrument. Previously, it was determined whether each sample was SARS-CoV-2 positive or negative by the confirmation test.

**< Review Comments to the Author >**

**Reviewer #1**

1. **In Table 2, the data for LoD test of the reference kit should be included for better comparison with that of two AccuPower® kits.**

🡪 As you kindly recommended, we added LoD data of the reference kit (STANDARD M nCoV Real-Time Detection kit, SD BIOSENSOR) in clinical trials for comparison (page 13, Table 2). LoD results of STANDARD M nCoV Real-Time Detection kit were referenced from Instruction for Use (IFU) Rev1 (SD BIOSENSOR, May 14, 2020).

| **RT-PCR kit** | **Mix type** | **RNA extraction** | **PCR equipment** | **Target genes** | **Limit of detection** |
| --- | --- | --- | --- | --- | --- |
|  |  | **equipment** |  |  |  |
| ***AccuPower*^®^ COVID-19 Multiplex**  **Real-Time RT-PCR Kit** | Premix | *ExiStation*^™^ 48 | *ExiStation*^™^ 48 | Pan-Sarbecovirus gene SARS-CoV-2 gene | Pan-Sarbecovirus gene:  120 copies/mL |
|  |  |  |  |  | SARS-CoV-2 gene:  120 copies/mL |
| ***AccuPower*^®^ SARS-CoV-2 Multiplex Real-Time RT-PCR Kit** | Master mix | *ExiPrep*^™^48 DX | *Exicycler*^™^96 | Pan-Sarbecovirus gene SARS-CoV-2 gene | Pan-Sarbecovirus gene:  2 copies/μL |
|  |  |  |  |  | SARS-CoV-2 gene:  2 copies/μL |
| **STANDARD™ M nCoV Real-Time Detection kit** | Master mix | QIAamp Viral RNA mini kit | CFX96^™^Dx System | ORF1ab gene  E gene | ORF1ab gene:  NP swab 0.25 copies/μL  Sputum 0.125 copies/μL  E gene:  NP swab 0.25 copies/μL  Sputum 0.25 copies/μL |

2. **In general, it is suitable that the cutoff Ct value for real-time RT-PCR is set to 35. Please explain the rationality that the cutoff Ct value is set to 40 in this study.**

🡪 For the cutoff Ct value of *Accupower***^®^** kits, the optimal cutoff Ct value is set by drawing an ROC curve using the LoD(Limit of Detection) analysis result, the low titer positive clinical sample result, and the LoB(Limit of Blank) result.

As a result of ROC Curve confirmation, in the case of NCVM, the Pan-Sarbecovirus gene was 40 Ct (specificity: 100.0%, sensitivity: 94.9.0%, AUC:98.9%), and the SARS-CoV-2 gene was 39 Ct (specificity: 100.0%, sensitivity:96.8%, AUC:98.8%). In the case of SCVM, the Pan-Sarbecovirus gene is 35 Ct (specificity: 100.0%, sensitivity:99.4%, AUC:99.9%), and the SARS-CoV-2 gene is 34Ct (specificity: 100.0%, sensitivity:98.6%, AUC:99.5%) was confirmed.

| ***AccuPower*^®^ COVID-19 Multiplex Real-Time**  **RT-PCR Kit (NCVM)** | ***AccuPower*^®^ SARS-CoV-2 Multiplex Real-Time**  **RT-PCR Kit (SCVM)** |
| --- | --- |
|  |  |
|  |  |

There is a difference in Ct depending on whether SCVM and NCVM use qRT-PCR protocol. Unlike NCVM, SCVM uses the Touch down qRT-PCR protocol to confirm that the Ct value of SCVM is pulled by about 3-5 Ct compared to NCVM at the same concentration. Therefore, when setting the cutoff Ct, the Ct values of LoD, LoB, and low titer positive samples differ by about 3-5 Ct, so the cutoff Ct value for NCVM was 40 Ct (or 39 Ct) and SCVM was found to be 35 Ct (or 34 Ct).

For a similar example, the STANDARD™ M nCoV Real-Time Detection kit (SD BIOSENSOR) which uses the same touch down PCR protocol as SCVM has the cutoff of 36Ct. On the other hand, Allplex™ 2019-nCoV Assay (Seegene) which does not use a touch down PCR protocol has a cutoff of 40Ct.

|  | ***AccuPower*^®^ COVID-19 Multiplex Real-Time RT-PCR Kit** | ***AccuPower*^®^ SARS-CoV-2 Multiplex Real-Time RT-PCR Kit** |
| --- | --- | --- |
| **qRT-PCR Protocol** |  | **<Touch down PCR protocol>**   |
| **Cutoff Ct value** | Pan-Sarbecovirus gene: 40 Ct  SARS-CoV-2 gene: 39Ct | Pan-Sarbecovirus gene: 35 Ct  SARS-CoV-2 gene: 34Ct |
| **Similar Product of other manufacturer** | **Allplex™ 2019-nCoV Assay (Seegene)**    Cutoff Ct: 40Ct | **STANDARDTM M nCoV Real-Time Detection kit (SD BIOSENSOR)**    Cutoff Ct: 36Ct |

3. **In S3 Fig, the comparison of the average Ct values should be performed between STANDARD™ M nCoV Real-Time Detection kit and NCVM.**

S3 Fig. is the analysis of Ct value for two types of kits, SCVM and the STANDARD™ M nCoV Real-Time Detection kit (SD), both of which are master mix type. NCVM, which is a premix type, was excluded from the analysis due to differences in reagent formulation and in qRT-PCR protocol (with or without touch down PCR protocol).

As you commented, the results of analyzing the Ct value between the STANDARD™ M nCoV Real-Time Detection kit (SD) and NCVM S3 Fig. are added as follows. In the ANOVA analysis of SCVM VS SD, the p-value is >0.05, which is not significant, but in the ANOVA analysis of NCVM VS SD, the p-value is <0.05 due to differences in reagent formulation and qRT-PCR protocol as described above.

In addition, since the previously submitted S3 Fig. only analyzed some of samples used in the clinical trial, S3 Fig. is now corrected with the re-analysis result including the total number of samples. (S3 Fig.)

4. **In view of the less sensitivity of two AccuPower® kits in detecting pooled samples, it is better to compare the average Ct values of NCVM and SCVM with that of the reference kit in pooled samples in Fig 3.**

The reference kit (STANDARD™ M nCoV Real-Time Detection kit, SD BIOSENSOR) did not claim pooling samples as usable samples and therefore we could not compare two *AccuPower*^®^ kits to STANDARD™ M nCoV Real-Time Detection kit.

Additionally, according to FDA guidelines, the results of individual samples in the test kit should be compared to those of reference kits (comparator method) and the results of pooled samples in the test kit should be compared to those of individual samples in the same kit (Expected result based on individually test result). We present a guideline for test and analysis in sample pooling, which compares Ct values between individual and pooled samples.

**<Molecular Diagnostic Template for Commercial Manufacturers (FDA, Version July 28, 2020)>**

5. **In Fig 4C, the data of Ct value comparison mentioned in figure legend between saliva and NPS/OPS samples detected by STANDARD™ M nCoV Real-Time Detection kit is missing.**

The reference kit (STANDARD™ M nCoV Real-Time Detection kit, SD BIOSENSOR) was not tested as it did not claim Saliva samples as usable samples. The legend of Figure 4C was written incorrectly and was corrected as follows.

**Fig 4. Clinical performance of the *AccuPower*^®^ kits in saliva and NPS/OPS samples** (A) Ct values of saliva and NPS/OPS samples from patients in various stages of COVID-19. (B) Clinical performance comparison of the *AccuPower*^®^ kits in saliva and NPS/OPS samples shown in scatter plots. (C) Ct value comparison by boxplot among the NCVM and SCVM. ASX, Asymptomatic.

6. **The advantage and disadvantage of two AccuPower® kits compared to the commercial kit and the range of their clinical application should be addressed in discussion section.**

We are already addressed advantage of *AccuPower*^®^ kits. As you commented we added limitations(or disadvantage) of two *AccuPower*^®^ kits. (Page 26)

In this study, *AccuPower*^®^ kits has some limitations. The first is *AccuPower*^®^ kit uses the same fluorescent dye for the RdRp gene and the N gene, so individual detection is impossible. In another thing, in the clinical trial using saliva, there was no EUA RT-PCR assay claiming saliva at the time of the test, so comparison by kit between saliva-saliva was not possible. Therefore, if a comparison with a reference kit claiming saliva is performed later, this part can be supplemented.

In conclusion, this study describes the successful development of two multiplex real-time RT-PCR methods, NCVM and SCVM, for the diagnosis of SARS-CoV-2. Simultaneous targeting of three viral genes (RdRP, N, and E) by the *AccuPower*^®^ kits provides an accurate, reliable, and easy-to-use SARS-CoV-2 detection test.

NCVM was used as an automated system from RNA extraction to PCR with a vacuum dried premix type kit to minimize the hands-on step and user errors. SCVM can be used with other manufacturers' PCR instruments such as CFX96™ Dx Real-Time PCR Detection System(Bio-Rad), Applied Biosystems 7500 Fast Real-time PCR Instrument system(Thermo Fisher Scientific), QuantStudio™5 Real-Time PCR Instrument (Thermo Fisher Scientific).

The *AccuPower*^®^ kits demonstrate the analytical performance characteristics expected of a valid diagnostic assay. The clinical performance of the *AccuPower*^®^ kits was comparable to the gold standard confirmation test, including the reference kit. In addition, the specimen pooling test with n=5 showed the ability of the *AccuPower*^®^ to process high volume samples cost-effectively for use as a surveillance tool. The clinical performance test of *AccuPower*^®^ kits in saliva samples demonstrated the usability of the *AccuPower*^®^ kits with saliva samples and the saliva samples being more adequate than NPS/OPS samples for early detection (before 10 days from the onset of symptom) of COVID-19. These data demonstrate that the *AccuPower*^®^ assays can be used for the fast and dependable detection of the SARS-CoV-2 virus.

7. **The primers and probe information are missing and should be included in the paper.**

Primer and probe information (sequence, etc.) cannot be disclosed as confidential data of the manufacturer.

**Minor comments**

1) **Have the authors verified that the assays are equally sensitive in detecting different circulating SARS-CoV-2 variants?**

SARS-CoV-2 variants analysis was performed as an *in-silico* analysis.

For sequence *In-silico* analysis of *AccuPower*^®^ kis alignment was performed for the following SARS-CoV-2 strain in the GISAID as Aug 30 to Sep 30, 2021(Location : North America, USA).

SARS-CoV-2 alpha variants (known as B.1.1.7, n=9,064), beta variants (known as B.1.351, n=477), delta variants (known as B.1.617.2, n=37,317), gamma variants (known as P.1, n=2,058), Mu variants (known as B.1.621, n=2,756) and lambda variants (known as C.37, n=229) were analyzed for coverage and homology with Accupower kit. Jalview was used for analysis.

The coverage of the using oligomers was analyzed individually by comparing and analyzing a total sequence, except to un-sequenced results (e.g –NNNNN-). The Table below shows the results of this coverage analysis.

| **Target** | **oligo** | **Alpha variant (n=9,064)** | | **Beta variant (n=477)** | | **Delta variant (n=37,317)** | | **Gamma variant (n=2058)** | |
| --- | --- | --- | --- | --- | --- | --- | --- | --- | --- |
|  |  | **match** | **Coverage** | **match** | **Coverage** | **match** | **Coverage** | **match** | **Coverage** |
| E gene set1 | Forward primer | 9053 | 99.85% | 462 | 100.00% | 37247 | 99.81% | 1,945 | 100.00% |
|  | Reverse primer | 9064 | 100% | 462 | 100.00% | 37284 | 99.91% | 1,944 | 99.95% |
|  | Probe | 9062 | 99.95% | 462 | 100.00% | 37241 | 99.80% | 1,944 | 99.95% |
| RdRP-1 | Forward primer | 9039 | 99.7% | 462 | 100.00% | 36685 | 98.31% | 1,945 | 100.00% |
|  | Reverse primer 1 | 0.00% | 0.00% | 0 | 0.00% | 0 | 0.00% | 0 | 0.00% |
|  | Reverse primer 2 | 9048 | 99.8% | 462 | 100.00% | 37237 | 99.79% | 1,940 | 99.74% |
|  | Probe | 9062 | 99.7% | 462 | 100.00% | 37239 | 99.79% | 1,940 | 99.74% |
| SARS-CoV-2 (RdRP set 2) | Forward primer | 9063 | 99.99% | 462 | 100.00% | 37264 | 99.76% | 1,945 | 100.00% |
|  | Reverse primer | 9064 | 100% | 462 | 100.00% | 37315 | 99.99% | 1,945 | 100.00% |
|  | Probe | 9062 | 99.98% | 462 | 100.00% | 37274 | 99.88% | 1,945 | 100.00% |
| SARS-CoV-2 (RdRP set 3) | Forward primer | 9064 | 100% | 462 | 100.00% | 37243 | 99.80% | 1,940 | 99.74% |
|  | Reverse primer | 9064 | 100% | 462 | 100.00% | 37285 | 99.91% | 1,945 | 100.00% |
|  | Probe | 9058 | 99.93% | 461 | 99.78% | 37229 | 99.76% | 1,944 | 99.95% |
| SARS-CoV-2 (N gene) | Forward primer | 9062 | 99.95% | 462 | 100.00% | 36837 | 98.71% | 1,916 | 98.51% |
|  | Reverse primer | 9060 | 99.93% | 462 | 100.00% | 37089 | 99.39% | 1,906 | 97.99% |
|  | Probe | 9060 | 99.98% | 461 | 99.78% | 37172 | 99.61% | 1,929 | 99.18% |
| **Target** | **oligo** | **Lambda variant (n=229)** | | **mu variant (n=2,756)** | |  | |  | |
|  |  | **match** | **Coverage** | **match** | **Coverage** |  |  |  |  |
| E gene set1 | Forward primer | 229 | 100% | 2,756 | 100% |  |  |  |  |
|  | Reverse primer | 229 | 100% | 2,756 | 100% |  |  |  |  |
|  | Probe | 229 | 100% | 2,756 | 100% |  |  |  |  |
| RdRP-1 | Forward primer | 229 | 100% | 2,756 | 100% |  |  |  |  |
|  | Reverse primer 1 | 229 | 0.00% | 0.00% | 0.00% |  |  |  |  |
|  | Reverse primer 2 | 229 | 100% | 2,756 | 100% |  |  |  |  |
|  | Probe | 229 | 100% | 2,756 | 100% |  | |  | |
| SARS-CoV-2 (RdRP set 2) | Forward primer | 229 | 100% | 2,756 | 100% |  |  |  |  |
|  | Reverse primer | 229 | 100% | 2,756 | 100% |  |  |  |  |
|  | Probe | 229 | 100% | 2,756 | 100% |  |  |  |  |
| SARS-CoV-2 (RdRP set 3) | Forward primer | 229 | 100% | 2,756 | 100% |  |  |  |  |
|  | Reverse primer | 229 | 100% | 2,756 | 100% |  |  |  |  |
|  | Probe | 229 | 100% | 2,756 | 100% |  |  |  |  |
| SARS-CoV-2 (N gene) | Forward primer | 228 | 99.56% | 2,756 | 100% |  |  |  |  |
|  | Reverse primer | 229 | 100% | 2,756 | 100% |  |  |  |  |
|  | Probe | 229 | 100% | 2,756 | 100% |  |  |  |  |

2) **Could the authors provide more detailed data regarding the cross-reactivity test?**

Several potentially cross-reactive organisms were spiked in simulated clinical matrix with presence of SARS-CoV-2 RNA (3X LoD concentration, Virus-Like Particles, Isolate USA-WA1/2020, Heat Inactivated). Then, spiked matrix was compared to control matrix (Sputum & NPS/OPS) which contained SARS-CoV-2 RNA and no spiked organisms. Each sample for each organism was tested in three replicates. Ct values of the control matrix were averaged and set as the control value. And Ct values of each sample was averaged and set as each testing group value. Difference between average value of testing group and the control was calculated to evaluate whether all samples were within the specified acceptance criteria or not. It was confirmed that the CV (%) and the % change of microbial interferent result are smaller than the acceptance criterion. All tested organisms were shown no influence in performance of the *AccuPower*^®^ COVID-19 Multiplex Real-Time RT-PCR Kit and *AccuPower*^®^ SARS-CoV-2 Multiplex Real-Time RT-PCR Kit at the 3XLoD concentrations evaluated with regards to analytical specificity and sensitivity.

**List of organisms to be tested**

| **No** | **Organism** | **Reference** | **No** | **Organism** | **Reference** |
| --- | --- | --- | --- | --- | --- |
| **1** | Human Influenza virus A H3N2 | KBPV-VR-71 | **20** | Parainfluenza virus 4a | KBPV-VR-69 |
| **2** | Human Influenza virus A H1N1 | KBPV-VR-76 | **21** | Chlamydia pneumoniae | ATCC 53592 |
| **3** | Human Influenza virus B (Texas/6/11) (Victoria) | 0810242CFHI | **22** | Haemophilus influenzae | ATCC 31441 |
| **4** | Human Coronavirus 229E | KBPV-VR-9 | **23** | Legionella pneumophila | ATCC 33152 |
| **5** | Human Coronavirus NL63 | 0810228CF | **24** | Streptococcus pneumoniae | ATCC 49619 |
| **6** | Human Coronavirus OC43 | 0810024CF | **25** | Streptococcus pyrogenes | ATCC700294 |
| **7** | Human Respiratory syncytial virus A | KBPV-VR-41 | **26** | Bordetella pertussis | ATCC 12742 |
| **8** | Human Respiratory syncytial virus B | KBPV-VR-42 | **27** | Mycoplasma pneumoniae | ATCC 15531D |
| **9** | Human Rhinovirus 14 (type B) | KBPV-VR-39 | **28** | Pooled Human nasal wash - to represent diverse microbial flora in the human respiratory tract | 991-13-P-1 |
| **10** | Human Metapneumovirus(hMPV) | 0810156CF | **29** | NATtrol Coronavirus-SARS Stock (qualitative) (NATSARS-ST/ 2003-00592) | 324780 |
| **11** | Human Adenovirus type 3 (type B) | KBPV-VR-62 | **30** | Enterovirus 70 | KBPV-VR-55 |
| **12** | Enterovirus 71 | KBPV-VR-56 | **31** | Coxsackievrus B5 | KBPV-VR-17 |
| **13** | Mycobacterium tuberculosis H37Rv | NAT MTB(H37RV)-ST | **32** | Echovirus 25 | KBPV-VR-24 |
| **14** | MERS-CoV | 0505-0002 | **33** | Human Parachovirus 3 | 0810147CF |
| **15** | Human coronavirus HKU1  (HCOV-HKU1) | ATCC VR-3262SD | **34** | Mycobacterium fortuitum | ATCC6841 |
| **16** | Adenovirus(71) | VR-1D | **35** | Mycobacterium intracell | ATCC13950 |
| **17** | Parainfluenza virus 1 | KBPV-VR-44 | **36** | Mycobacterium gordonae | KCTC9513 |
| **18** | Parainfluenza virus 2 | KBPV-VR-45 | **37** | Mycobacterium chelonae | KCTC9505 |
| **19** | Parainfluenza virus 3 | KBPV-VR-68 | **38** | Pneumocystis jirovecii(PJP) | NATPJI-ERC |

**Microbial interference and Endogenous interference studies:**

- SARS-CoV-2 gene with interfering substance must be not detected for base matrix.
- Hit Rate at SARS-CoV-2 panel (3xLoD) : The observed positive hit rate for the 3XLoD Positive samples must be equal to 100%.
- 3XLoD Positive samples should be analyzed CV(%) < 5.
- The % change (% change; [Test Ct mean values-Baseline Ct mean values]/ baseline Ct x 100) for the SARS-CoV-2 RNA (3xLoD) must be less than ± 10% change to be considered stable.
