# Supplementary material for: Development and Evaluation of AccuPower COVID-19 Multiplex Real-Time RT-PCR Kit and AccuPower SARS-CoV-2 Multiplex Real-Time RT-PCR Kit for SARS-CoV-2 Detection in Sputum, NPS/OPS, Saliva and Pooled Samples
Source: PLoS One. 2022 Feb 10;17(2):e0263341. doi: 10.1371/journal.pone.0263341 (PMC8830688; doi:10.1371/journal.pone.0263341)
Supplement: Peer review history

# Peer review: Decision Letter 0

**DOI:** 10.1371/journal.pone.0263341.r001

**Authors:** Feng Gao

8 Nov 2021

PONE-D-21-30277Development and Evaluation of AccuPower® COVID-19 Multiplex Real-Time RT-PCR Kit and AccuPower® SARS-CoV-2 Multiplex Real-Time RT-PCR Kit for SARS-CoV-2 Detection in Sputum, NPS/OPS, Saliva and Pooled Samples.PLOS ONE

Dear Dr. Kim,

Thank you for submitting your manuscript to PLOS ONE. After careful consideration, we feel that it has merit but does not fully meet PLOS ONE’s publication criteria as it currently stands. Therefore, we invite you to submit a revised version of the manuscript that addresses the points raised during the review process.

ols: These article types are not expected to include results but may include pilot data. 

Please submit your revised manuscript by Dec 23 2021 11:59PM. If you will need more time than this to complete your revisions, please reply to this message or contact the journal office at plosone@plos.org. When you're ready to submit your revision, log on to https://www.editorialmanager.com/pone/ and select the 'Submissions Needing Revision' folder to locate your manuscript file.

Please include the following items when submitting your revised manuscript:

- A rebuttal letter that responds to each point raised by the academic editor and reviewer(s). You should upload this letter as a separate file labeled 'Response to Reviewers'.
- A marked-up copy of your manuscript that highlights changes made to the original version. You should upload this as a separate file labeled 'Revised Manuscript with Track Changes'.
- An unmarked version of your revised paper without tracked changes. You should upload this as a separate file labeled 'Manuscript'.

If you would like to make changes to your financial disclosure, please include your updated statement in your cover letter. Guidelines for resubmitting your figure files are available below the reviewer comments at the end of this letter.

If applicable, we recommend that you deposit your laboratory protocols in protocols.io to enhance the reproducibility of your results. Protocols.io assigns your protocol its own identifier (DOI) so that it can be cited independently in the future. For instructions see: https://journals.plos.org/plosone/s/submission-guidelines#loc-laboratory-protocols. Additionally, PLOS ONE offers an option for publishing peer-reviewed Lab Protocol articles, which describe protocols hosted on protocols.io. Read more information on sharing protocols at https://plos.org/protocols?utm_medium=editorial-email&utm_source=authorletters&utm_campaign=protocols.

We look forward to receiving your revised manuscript.

Kind regards,

Feng Gao

Academic Editor

PLOS ONE

Journal requirements:

When submitting your revision, we need you to address these additional requirements.

1. Please ensure that your manuscript meets PLOS ONE's style requirements, including those for file naming. The PLOS ONE style templates can be found at

https://journals.plos.org/plosone/s/file?id=wjVg/PLOSOne_formatting_sample_main_body.pdf  and

https://journals.plos.org/plosone/s/file?id=ba62/PLOSOne_formatting_sample_title_authors_affiliations.pdf

2. Thank you for stating the following in the Acknowledgments Section of your manuscript:

“This research was supported by a grant of the Korea Health Technology R&D Project through the Korea Health Industry Development Institute (KHIDI), funded by the Ministry of Health & Welfare, Republic of Korea (grant number: HI20C2557).”

We note that you have provided additional information within the Acknowledgements Section that is not currently declared in your Funding Statement. Please note that funding information should not appear in the Acknowledgments section or other areas of your manuscript. We will only publish funding information present in the Funding Statement section of the online submission form.

Please remove any funding-related text from the manuscript and let us know how you would like to update your Funding Statement. Currently, your Funding Statement reads as follows:

“Initials of the authors who received each award : MJ Kim

Grant numbers awarded to each author : HI20C2557000020 

The full name of each funder : Korea Health Industry Development Institute (KHIDI)

URL of each funder website : https://www.htdream.kr/

Did the sponsors or funders play any role in the study design, data collection and analysis, decision to publish, or preparation of the manuscript? No”

Please include your amended statements within your cover letter; we will change the online submission form on your behalf.

3. Thank you for stating the following financial disclosure:

“Thank you for stating the following financial disclosure:

“Initials of the authors who received each award : MJ Kim

Grant numbers awarded to each author : HI20C2557000020 

The full name of each funder : Korea Health Industry Development Institute (KHIDI)

URL of each funder website : https://www.htdream.kr/

Did the sponsors or funders play any role in the study design, data collection and analysis, decision to publish, or preparation of the manuscript? No”

Please state what role the funders took in the study.  If the funders had no role, please state: "The funders had no role in study design, data collection and analysis, decision to publish, or preparation of the manuscript."

If this statement is not correct you must amend it as needed.

Please include this amended Role of Funder statement in your cover letter; we will change the online submission form on your behalf.”

Please state what role the funders took in the study.  If the funders had no role, please state: "The funders had no role in study design, data collection and analysis, decision to publish, or preparation of the manuscript."

If this statement is not correct you must amend it as needed.

Please include this amended Role of Funder statement in your cover letter; we will change the online submission form on your behalf.

4. Please include your full ethics statement in the ‘Methods’ section of your manuscript file. In your statement, please include the full name of the IRB or ethics committee who approved or waived your study, as well as whether or not you obtained informed written or verbal consent. If consent was waived for your study, please include this information in your statement as well.

Please review your reference list to ensure that it is complete and correct. If you have cited papers that have been retracted, please include the rationale for doing so in the manuscript text, or remove these references and replace them with relevant current references. Any changes to the reference list should be mentioned in the rebuttal letter that accompanies your revised manuscript. If you need to cite a retracted article, indicate the article’s retracted status in the References list and also include a citation and full reference for the retraction notice.

[Note: HTML markup is below. Please do not edit.]

Reviewers' comments:

Reviewer's Responses to Questions

**Comments to the Author**

1. Is the manuscript technically sound, and do the data support the conclusions?

The manuscript must describe a technically sound piece of scientific research with data that supports the conclusions. Experiments must have been conducted rigorously, with appropriate controls, replication, and sample sizes. The conclusions must be drawn appropriately based on the data presented. 

Reviewer #1: Yes

Reviewer #2: Yes

**********

2. Has the statistical analysis been performed appropriately and rigorously? 

Reviewer #1: Yes

Reviewer #2: I Don't Know

**********

3. Have the authors made all data underlying the findings in their manuscript fully available?

The PLOS Data policy requires authors to make all data underlying the findings described in their manuscript fully available without restriction, with rare exception (please refer to the Data Availability Statement in the manuscript PDF file). The data should be provided as part of the manuscript or its supporting information, or deposited to a public repository. For example, in addition to summary statistics, the data points behind means, medians and variance measures should be available. If there are restrictions on publicly sharing data—e.g. participant privacy or use of data from a third party—those must be specified.

Reviewer #1: Yes

Reviewer #2: Yes

**********

4. Is the manuscript presented in an intelligible fashion and written in standard English?

PLOS ONE does not copyedit accepted manuscripts, so the language in submitted articles must be clear, correct, and unambiguous. Any typographical or grammatical errors should be corrected at revision, so please note any specific errors here.

Reviewer #1: Yes

Reviewer #2: Yes

**********

5. Review Comments to the Author

Please use the space provided to explain your answers to the questions above. You may also include additional comments for the author, including concerns about dual publication, research ethics, or publication ethics. (Please upload your review as an attachment if it exceeds 20,000 characters)

Reviewer #1: In this work, Bum et al developed two novel AccuPower® multiplex real-time RT-PCR kits and evaluated the clinical performance comparable to the gold standard confirmation test and reference kit for the detection of SARS-CoV-2 infection by testing multiple kinds of clinical samples, which supported the adequacy of two AccuPower® kits for clinical application. The data presented for the performance characteristics of two AccuPower® kits are intact and reliable supported by using a large number of clinical samples, however, the sensitivity of them are slightly less than that of reference kit in pooled samples detection which may limit further clinical application. Overall, this study is of general interest to the readership of the Journal of PLOS ONE. However, the authors should consider the following points to further strengthen the paper.

Specific points:

1. In Table 2, the data for LoD test of the reference kit should be included for better comparison with that of two AccuPower® kits.

2. In general, it is suitable that the cutoff Ct value for real-time RT-PCR is set to 35. Please explain the rationality that the cutoff Ct value is set to 40 in this study.

3. In S3 Fig, the comparison of the average Ct values should be performed between STANDARD™ M nCoV Real-Time Detection kit and NCVM.

4. In view of the less sensitivity of two AccuPower® kits in detecting pooled samples, it is better to compare the average Ct values of NCVM and SCVM with that of the reference kit in pooled samples in Fig 3.

5. In Fig 4C, the data of Ct value comparison mentioned in figure legend between saliva and NPS/OPS samples detected by STANDARD™ M nCoV Real-Time Detection kit is missing.

6. The advantage and disadvantage of two AccuPower® kits compared to the commercial kit and the range of their clinical application should be addressed in discussion section.

7. The primers and probe information are missing and should be included in the paper.

Reviewer #2: In the current manuscript, Bum et. al. developed and evaluated the AccuPower® COVID-19 Multiplex Real-Time RT-PCR Kit and the AccuPower® SARS-CoV-2 Multiplex Real-Time RT-PCR Kit for SARS-CoV-2 detection. The authors evaluated the limit of detection (LoD), cross-reactivity, and precision of the assays and concluded that the BIONEER NCVM and SCVM assays are comparable with the current standard confirmation assay and are suitable for effective clinical management.

Minor comments:

1) Have the authors verified that the assays are equally sensitive in detecting different circulating SARS-CoV-2 variants?

2) Could the authors provide more detailed data regarding the cross-reactivity test?

**********

6. PLOS authors have the option to publish the peer review history of their article (what does this mean?). If published, this will include your full peer review and any attached files.

If you choose “no”, your identity will remain anonymous but your review may still be made public.

**Do you want your identity to be public for this peer review?** For information about this choice, including consent withdrawal, please see our Privacy Policy.

Reviewer #1: No

Reviewer #2: No

[NOTE: If reviewer comments were submitted as an attachment file, they will be attached to this email and accessible via the submission site. Please log into your account, locate the manuscript record, and check for the action link "View Attachments". If this link does not appear, there are no attachment files.]

While revising your submission, please upload your figure files to the Preflight Analysis and Conversion Engine (PACE) digital diagnostic tool, https://pacev2.apexcovantage.com/. PACE helps ensure that figures meet PLOS requirements. To use PACE, you must first register as a user. Registration is free. Then, login and navigate to the UPLOAD tab, where you will find detailed instructions on how to use the tool. If you encounter any issues or have any questions when using PACE, please email PLOS at figures@plos.org. Please note that Supporting Information files do not need this step.

# Author comment: Author response to Decision Letter 0

**DOI:** 10.1371/journal.pone.0263341.r002

21 Dec 2021

Responding to Reviewers

Thank you for reviewing the manuscript. We respond to all of your comments as follows.

< Journal requirements >

1. Please ensure that your manuscript meets PLOS ONE's style requirements, including those for file naming. The PLOS ONE style templates can be found at

https://journals.plos.org/plosone/s/file?id=wjVg/PLOSOne_formatting_sample_main_body.pdf and

https://journals.plos.org/plosone/s/file?id=ba62/PLOSOne_formatting_sample_title_authors_affiliations.pdf

� As you commented, we uploaded files labeled ‘Response to Reviewers’, ‘Revised Manuscript with Track Changes’ and ‘Manuscript’. Uploaded files were written in PLOS ONE style templates. 

2. Please remove any funding-related text from the manuscript and let us know how you would like to update your Funding Statement. 

� As you commented, we removed funding-related text. (page 27)

3. Please state what role the funders took in the study. If the funders had no role, please state: "The funders had no role in study design, data collection and analysis, decision to publish, or preparation of the manuscript." If this statement is not correct you must amend it as needed. Please include this amended Role of Funder statement in your cover letter; we will change the online submission form on your behalf.” 

� The funders had no role in study design, data collection and analysis, decision to publish, or preparation of the manuscript.

4. Please include your full ethics statement in the ‘Methods’ section of your manuscript file. In your statement, please include the full name of the IRB or ethics committee who approved or waived your study, as well as whether or not you obtained informed written or verbal consent. 

� As you commented, the ethics statement has been added to the 'Method_Ethics Statement and Sample Collection' section (page 8).

Ethics Statement and Sample Collection 

Authors confirm that this paper has complied with PLOS policies on research ethics, as stated in submission guide. Since all identification information of samples was anonymized by replacing it with the unique identification code of the institution, researchers have performed the clinical performance test without risks of identifying or tracing personal information with the sample. The protocol of the study was approved by the Institutional Review Board (IRB) of Myongji Hospital and IRB of Kangwon National University Hospital. Since samples were residual sample with guaranteed anonymity and there was no need to collect additional samples for this study, there was no risk to research subjects in accordance with ‘Bioethics and Safety Act’ and Article 13 of ‘Enforcement Rules of the In Vitro Diagnostic Medical Devices Act’. Hence, IRBs confirmed that this study does not need subject consent. This study was conducted in accordance with the principles of the Declaration of Helsinki, 1964.

Myongji Hospital Institutional Review Board (IRB) approved the use of surplus clinical samples for the NPS/OPS specimen-pooling (IRB No.: MJH 2020-12-028) and clinical performance evaluation (IRB No.: MJH 2020-12-029) tests. Kangwon National University Hospital IRB approved the use of saliva and NPS/OPS samples for the clinical performance testing of the AccuPower® kits (IRB No.: KNUH-2021-03-014), which were either surplus samples or de-identified patient samples drawn after informed consent (Fig 1). RNA in the NPS/OPS and sputum samples was extracted using the ExiPrep™48 Viral DNA/RNA Kit (BIONEER, Korea) with the ExiPrep™48 Dx instrument and the RNA of saliva samples was extracted using the ExiPrep™48 Fast Viral RNA Kit (BIONEER, Korea) with the ExiPrep™48 Dx instrument. Previously, it was determined whether each sample was SARS-CoV-2 positive or negative by the confirmation test.

< Review Comments to the Author >

Reviewer #1

1. In Table 2, the data for LoD test of the reference kit should be included for better comparison with that of two AccuPower® kits.

� As you kindly recommended, we added LoD data of the reference kit (STANDARD M nCoV Real-Time Detection kit, SD BIOSENSOR) in clinical trials for comparison (page 13, Table 2). LoD results of STANDARD M nCoV Real-Time Detection kit were referenced from Instruction for Use (IFU) Rev1 (SD BIOSENSOR, May 14, 2020).

RT-PCR kit Mix type RNA extraction PCR equipment Target genes Limit of detection

 equipment 

AccuPower® COVID-19 Multiplex 

Real-Time RT-PCR Kit 　Premix ExiStation™ 48 ExiStation™ 48 Pan-Sarbecovirus gene SARS-CoV-2 gene Pan-Sarbecovirus gene:

 120 copies/mL 

 SARS-CoV-2 gene: 

120 copies/mL

AccuPower® SARS-CoV-2 Multiplex Real-Time RT-PCR Kit Master mix ExiPrep™48 DX Exicycler™96 Pan-Sarbecovirus gene SARS-CoV-2 gene Pan-Sarbecovirus gene:

 2 copies/μL 

 SARS-CoV-2 gene: 

2 copies/μL

STANDARD™ M nCoV Real-Time Detection kit Master mix QIAamp Viral RNA mini kit CFX96™Dx System ORF1ab gene

E gene ORF1ab gene:

NP swab 0.25 copies/μL

Sputum 0.125 copies/μL

E gene:

NP swab 0.25 copies/μL

Sputum 0.25 copies/μL

2. In general, it is suitable that the cutoff Ct value for real-time RT-PCR is set to 35. Please explain the rationality that the cutoff Ct value is set to 40 in this study.

� For the cutoff Ct value of Accupower® kits, the optimal cutoff Ct value is set by drawing an ROC curve using the LoD(Limit of Detection) analysis result, the low titer positive clinical sample result, and the LoB(Limit of Blank) result.

As a result of ROC Curve confirmation, in the case of NCVM, the Pan-Sarbecovirus gene was 40 Ct (specificity: 100.0%, sensitivity: 94.9.0%, AUC:98.9%), and the SARS-CoV-2 gene was 39 Ct (specificity: 100.0%, sensitivity:96.8%, AUC:98.8%). In the case of SCVM, the Pan-Sarbecovirus gene is 35 Ct (specificity: 100.0%, sensitivity:99.4%, AUC:99.9%), and the SARS-CoV-2 gene is 34Ct (specificity: 100.0%, sensitivity:98.6%, AUC:99.5%) was confirmed.

AccuPower® COVID-19 Multiplex Real-Time 

RT-PCR Kit (NCVM) AccuPower® SARS-CoV-2 Multiplex Real-Time 

RT-PCR Kit (SCVM)

There is a difference in Ct depending on whether SCVM and NCVM use qRT-PCR protocol. Unlike NCVM, SCVM uses the Touch down qRT-PCR protocol to confirm that the Ct value of SCVM is pulled by about 3-5 Ct compared to NCVM at the same concentration. Therefore, when setting the cutoff Ct, the Ct values of LoD, LoB, and low titer positive samples differ by about 3-5 Ct, so the cutoff Ct value for NCVM was 40 Ct (or 39 Ct) and SCVM was found to be 35 Ct (or 34 Ct).

For a similar example, the STANDARD™ M nCoV Real-Time Detection kit (SD BIOSENSOR) which uses the same touch down PCR protocol as SCVM has the cutoff of 36Ct. On the other hand, Allplex™ 2019-nCoV Assay (Seegene) which does not use a touch down PCR protocol has a cutoff of 40Ct.

 AccuPower® COVID-19 Multiplex Real-Time RT-PCR Kit AccuPower® SARS-CoV-2 Multiplex Real-Time RT-PCR Kit

qRT-PCR Protocol 

 <Touch down PCR protocol>

Cutoff Ct value Pan-Sarbecovirus gene: 40 Ct

SARS-CoV-2 gene: 39Ct Pan-Sarbecovirus gene: 35 Ct

SARS-CoV-2 gene: 34Ct

Similar Product of other manufacturer Allplex™ 2019-nCoV Assay (Seegene)

Cutoff Ct: 40Ct STANDARDTM M nCoV Real-Time Detection kit (SD BIOSENSOR)

Cutoff Ct: 36Ct

3. In S3 Fig, the comparison of the average Ct values should be performed between STANDARD™ M nCoV Real-Time Detection kit and NCVM.

S3 Fig. is the analysis of Ct value for two types of kits, SCVM and the STANDARD™ M nCoV Real-Time Detection kit (SD), both of which are master mix type. NCVM, which is a premix type, was excluded from the analysis due to differences in reagent formulation and in qRT-PCR protocol (with or without touch down PCR protocol).

As you commented, the results of analyzing the Ct value between the STANDARD™ M nCoV Real-Time Detection kit (SD) and NCVM S3 Fig. are added as follows. In the ANOVA analysis of SCVM VS SD, the p-value is >0.05, which is not significant, but in the ANOVA analysis of NCVM VS SD, the p-value is <0.05 due to differences in reagent formulation and qRT-PCR protocol as described above. 

In addition, since the previously submitted S3 Fig. only analyzed some of samples used in the clinical trial, S3 Fig. is now corrected with the re-analysis result including the total number of samples. (S3 Fig.)

4. In view of the less sensitivity of two AccuPower® kits in detecting pooled samples, it is better to compare the average Ct values of NCVM and SCVM with that of the reference kit in pooled samples in Fig 3.

The reference kit (STANDARD™ M nCoV Real-Time Detection kit, SD BIOSENSOR) did not claim pooling samples as usable samples and therefore we could not compare two AccuPower® kits to STANDARD™ M nCoV Real-Time Detection kit. 

Additionally, according to FDA guidelines, the results of individual samples in the test kit should be compared to those of reference kits (comparator method) and the results of pooled samples in the test kit should be compared to those of individual samples in the same kit (Expected result based on individually test result). We present a guideline for test and analysis in sample pooling, which compares Ct values between individual and pooled samples.

<Molecular Diagnostic Template for Commercial Manufacturers (FDA, Version July 28, 2020)>

5. In Fig 4C, the data of Ct value comparison mentioned in figure legend between saliva and NPS/OPS samples detected by STANDARD™ M nCoV Real-Time Detection kit is missing.

The reference kit (STANDARD™ M nCoV Real-Time Detection kit, SD BIOSENSOR) was not tested as it did not claim Saliva samples as usable samples. The legend of Figure 4C was written incorrectly and was corrected as follows. 

Fig 4. Clinical performance of the AccuPower® kits in saliva and NPS/OPS samples (A) Ct values of saliva and NPS/OPS samples from patients in various stages of COVID-19. (B) Clinical performance comparison of the AccuPower® kits in saliva and NPS/OPS samples shown in scatter plots. (C) Ct value comparison by boxplot among the NCVM and SCVM. ASX, Asymptomatic.

6. The advantage and disadvantage of two AccuPower® kits compared to the commercial kit and the range of their clinical application should be addressed in discussion section.

We are already addressed advantage of AccuPower® kits. As you commented we added limitations(or disadvantage) of two AccuPower® kits. (Page 26)

In this study, AccuPower® kits has some limitations. The first is AccuPower® kit uses the same fluorescent dye for the RdRp gene and the N gene, so individual detection is impossible. In another thing, in the clinical trial using saliva, there was no EUA RT-PCR assay claiming saliva at the time of the test, so comparison by kit between saliva-saliva was not possible. Therefore, if a comparison with a reference kit claiming saliva is performed later, this part can be supplemented. 

In conclusion, this study describes the successful development of two multiplex real-time RT-PCR methods, NCVM and SCVM, for the diagnosis of SARS-CoV-2. Simultaneous targeting of three viral genes (RdRP, N, and E) by the AccuPower® kits provides an accurate, reliable, and easy-to-use SARS-CoV-2 detection test. 

NCVM was used as an automated system from RNA extraction to PCR with a vacuum dried premix type kit to minimize the hands-on step and user errors. SCVM can be used with other manufacturers' PCR instruments such as CFX96™ Dx Real-Time PCR Detection System(Bio-Rad), Applied Biosystems 7500 Fast Real-time PCR Instrument system(Thermo Fisher Scientific), QuantStudio™5 Real-Time PCR Instrument (Thermo Fisher Scientific). 

The AccuPower® kits demonstrate the analytical performance characteristics expected of a valid diagnostic assay. The clinical performance of the AccuPower® kits was comparable to the gold standard confirmation test, including the reference kit. In addition, the specimen pooling test with n=5 showed the ability of the AccuPower® to process high volume samples cost-effectively for use as a surveillance tool. The clinical performance test of AccuPower® kits in saliva samples demonstrated the usability of the AccuPower® kits with saliva samples and the saliva samples being more adequate than NPS/OPS samples for early detection (before 10 days from the onset of symptom) of COVID-19. These data demonstrate that the AccuPower® assays can be used for the fast and dependable detection of the SARS-CoV-2 virus.

7. The primers and probe information are missing and should be included in the paper.

Primer and probe information (sequence, etc.) cannot be disclosed as confidential data of the manufacturer.

Minor comments

1) Have the authors verified that the assays are equally sensitive in detecting different circulating SARS-CoV-2 variants? 

SARS-CoV-2 variants analysis was performed as an in-silico analysis. 

For sequence In-silico analysis of AccuPower® kis alignment was performed for the following SARS-CoV-2 strain in the GISAID as Aug 30 to Sep 30, 2021(Location : North America, USA). 

SARS-CoV-2 alpha variants (known as B.1.1.7, n=9,064), beta variants (known as B.1.351, n=477), delta variants (known as B.1.617.2, n=37,317), gamma variants (known as P.1, n=2,058), Mu variants (known as B.1.621, n=2,756) and lambda variants (known as C.37, n=229) were analyzed for coverage and homology with Accupower kit. Jalview was used for analysis. 

The coverage of the using oligomers was analyzed individually by comparing and analyzing a total sequence, except to un-sequenced results (e.g –NNNNN-). The Table below shows the results of this coverage analysis.

Target oligo Alpha variant (n=9,064) Beta variant (n=477) Delta variant (n=37,317) Gamma variant (n=2058)

 match Coverage match Coverage match Coverage match Coverage

E gene set1 Forward primer 9053 99.85% 462 100.00% 37247 99.81% 1,945 100.00%

 Reverse primer 9064 100% 462 100.00% 37284 99.91% 1,944 99.95%

 Probe 9062 99.95% 462 100.00% 37241 99.80% 1,944 99.95%

RdRP-1 Forward primer 9039 99.7% 462 100.00% 36685 98.31% 1,945 100.00%

 Reverse primer 1 0.00% 0.00% 0 0.00% 0 0.00% 0 0.00%

 Reverse primer 2 9048 99.8% 462 100.00% 37237 99.79% 1,940 99.74%

 Probe 9062 99.7% 462 100.00% 37239 99.79% 1,940 99.74%

SARS-CoV-2 (RdRP set 2) Forward primer 9063 99.99% 462 100.00% 37264 99.76% 1,945 100.00%

 Reverse primer 9064 100% 462 100.00% 37315 99.99% 1,945 100.00%

 Probe 9062 99.98% 462 100.00% 37274 99.88% 1,945 100.00%

SARS-CoV-2 (RdRP set 3) Forward primer 9064 100% 462 100.00% 37243 99.80% 1,940 99.74%

 Reverse primer 9064 100% 462 100.00% 37285 99.91% 1,945 100.00%

 Probe 9058 99.93% 461 99.78% 37229 99.76% 1,944 99.95%

SARS-CoV-2 (N gene) Forward primer 9062 99.95% 462 100.00% 36837 98.71% 1,916 98.51%

 Reverse primer 9060 99.93% 462 100.00% 37089 99.39% 1,906 97.99%

 Probe 9060 99.98% 461 99.78% 37172 99.61% 1,929 99.18%

Target oligo Lambda variant (n=229) mu variant (n=2,756) 

 match Coverage match Coverage 

E gene set1 Forward primer 229 100% 2,756 100% 

 Reverse primer 229 100% 2,756 100% 

 Probe 229 100% 2,756 100% 

RdRP-1 Forward primer 229 100% 2,756 100% 

 Reverse primer 1 229 0.00% 0.00% 0.00% 

 Reverse primer 2 229 100% 2,756 100% 

 Probe 229 100% 2,756 100% 

SARS-CoV-2 (RdRP set 2) Forward primer 229 100% 2,756 100% 

 Reverse primer 229 100% 2,756 100% 

 Probe 229 100% 2,756 100% 

SARS-CoV-2 (RdRP set 3) Forward primer 229 100% 2,756 100% 

 Reverse primer 229 100% 2,756 100% 

 Probe 229 100% 2,756 100% 

SARS-CoV-2 (N gene) Forward primer 228 99.56% 2,756 100% 

 Reverse primer 229 100% 2,756 100% 

 Probe 229 100% 2,756 100% 

2) Could the authors provide more detailed data regarding the cross-reactivity test?

Several potentially cross-reactive organisms were spiked in simulated clinical matrix with presence of SARS-CoV-2 RNA (3X LoD concentration, Virus-Like Particles, Isolate USA-WA1/2020, Heat Inactivated). Then, spiked matrix was compared to control matrix (Sputum & NPS/OPS) which contained SARS-CoV-2 RNA and no spiked organisms. Each sample for each organism was tested in three replicates. Ct values of the control matrix were averaged and set as the control value. And Ct values of each sample was averaged and set as each testing group value. Difference between average value of testing group and the control was calculated to evaluate whether all samples were within the specified acceptance criteria or not. It was confirmed that the CV (%) and the % change of microbial interferent result are smaller than the acceptance criterion. All tested organisms were shown no influence in performance of the AccuPower® COVID-19 Multiplex Real-Time RT-PCR Kit and AccuPower® SARS-CoV-2 Multiplex Real-Time RT-PCR Kit at the 3XLoD concentrations evaluated with regards to analytical specificity and sensitivity.

List of organisms to be tested

No Organism Reference No Organism Reference

1 Human Influenza virus A H3N2 KBPV-VR-71 20 Parainfluenza virus 4a KBPV-VR-69

2 Human Influenza virus A H1N1 KBPV-VR-76 21 Chlamydia pneumoniae ATCC 53592

3 Human Influenza virus B (Texas/6/11) (Victoria) 0810242CFHI 22 Haemophilus influenzae ATCC 31441

4 Human Coronavirus 229E KBPV-VR-9 23 Legionella pneumophila ATCC 33152

5 Human Coronavirus NL63 0810228CF 24 Streptococcus pneumoniae ATCC 49619

6 Human Coronavirus OC43 0810024CF 25 Streptococcus pyrogenes ATCC700294

7 Human Respiratory syncytial virus A KBPV-VR-41 26 Bordetella pertussis ATCC 12742

8 Human Respiratory syncytial virus B KBPV-VR-42 27 Mycoplasma pneumoniae ATCC 15531D

9 Human Rhinovirus 14 (type B) KBPV-VR-39 28 Pooled Human nasal wash - to represent diverse microbial flora in the human respiratory tract 991-13-P-1

10 Human Metapneumovirus(hMPV) 0810156CF 29 NATtrol Coronavirus-SARS Stock (qualitative) (NATSARS-ST/ 2003-00592) 324780

11 Human Adenovirus type 3 (type B) KBPV-VR-62 30 Enterovirus 70 KBPV-VR-55

12 Enterovirus 71 KBPV-VR-56 31 Coxsackievrus B5 KBPV-VR-17

13 Mycobacterium tuberculosis H37Rv NAT MTB(H37RV)-ST 32 Echovirus 25 KBPV-VR-24

14 MERS-CoV 0505-0002 33 Human Parachovirus 3 0810147CF

15 Human coronavirus HKU1

(HCOV-HKU1) ATCC VR-3262SD 34 Mycobacterium fortuitum ATCC6841

16 Adenovirus(71) VR-1D 35 Mycobacterium intracell ATCC13950

17 Parainfluenza virus 1 KBPV-VR-44 36 Mycobacterium gordonae KCTC9513

18 Parainfluenza virus 2 KBPV-VR-45 37 Mycobacterium chelonae KCTC9505

19 Parainfluenza virus 3 KBPV-VR-68 38 Pneumocystis jirovecii(PJP) NATPJI-ERC

Microbial interference and Endogenous interference studies:

- SARS-CoV-2 gene with interfering substance must be not detected for base matrix.

- Hit Rate at SARS-CoV-2 panel (3xLoD) : The observed positive hit rate for the 3XLoD Positive samples must be equal to 100%.

- 3XLoD Positive samples should be analyzed CV(%) < 5.

- The % change (% change; [Test Ct mean values-Baseline Ct mean values]/ baseline Ct x 100) for the SARS-CoV-2 RNA (3xLoD) must be less than ± 10% change to be considered stable.

Attachment Submitted filename: Response to Reviewers.docx Click here for additional data file.

# Peer review: Decision Letter 1

**DOI:** 10.1371/journal.pone.0263341.r003

**Authors:** Feng Gao

18 Jan 2022

Development and Evaluation of AccuPower® COVID-19 Multiplex Real-Time RT-PCR Kit and AccuPower® SARS-CoV-2 Multiplex Real-Time RT-PCR Kit for SARS-CoV-2 Detection in Sputum, NPS/OPS, Saliva and Pooled Samples.

PONE-D-21-30277R1

Dear Dr. Kim,

We’re pleased to inform you that your manuscript has been judged scientifically suitable for publication and will be formally accepted for publication once it meets all outstanding technical requirements.

Within one week, you’ll receive an e-mail detailing the required amendments. When these have been addressed, you’ll receive a formal acceptance letter and your manuscript will be scheduled for publication.

An invoice for payment will follow shortly after the formal acceptance. To ensure an efficient process, please log into Editorial Manager at http://www.editorialmanager.com/pone/, click the 'Update My Information' link at the top of the page, and double check that your user information is up-to-date. If you have any billing related questions, please contact our Author Billing department directly at authorbilling@plos.org.

If your institution or institutions have a press office, please notify them about your upcoming paper to help maximize its impact. If they’ll be preparing press materials, please inform our press team as soon as possible -- no later than 48 hours after receiving the formal acceptance. Your manuscript will remain under strict press embargo until 2 pm Eastern Time on the date of publication. For more information, please contact onepress@plos.org.

Kind regards,

Feng Gao

Academic Editor

PLOS ONE

Additional Editor Comments (optional):

Reviewers' comments:

Reviewer's Responses to Questions

**Comments to the Author**

1. If the authors have adequately addressed your comments raised in a previous round of review and you feel that this manuscript is now acceptable for publication, you may indicate that here to bypass the “Comments to the Author” section, enter your conflict of interest statement in the “Confidential to Editor” section, and submit your "Accept" recommendation.

Reviewer #1: All comments have been addressed

Reviewer #2: All comments have been addressed

**********

2. Is the manuscript technically sound, and do the data support the conclusions?

The manuscript must describe a technically sound piece of scientific research with data that supports the conclusions. Experiments must have been conducted rigorously, with appropriate controls, replication, and sample sizes. The conclusions must be drawn appropriately based on the data presented. 

Reviewer #1: Yes

Reviewer #2: Yes

**********

3. Has the statistical analysis been performed appropriately and rigorously? 

Reviewer #1: I Don't Know

Reviewer #2: I Don't Know

**********

4. Have the authors made all data underlying the findings in their manuscript fully available?

The PLOS Data policy requires authors to make all data underlying the findings described in their manuscript fully available without restriction, with rare exception (please refer to the Data Availability Statement in the manuscript PDF file). The data should be provided as part of the manuscript or its supporting information, or deposited to a public repository. For example, in addition to summary statistics, the data points behind means, medians and variance measures should be available. If there are restrictions on publicly sharing data—e.g. participant privacy or use of data from a third party—those must be specified.

Reviewer #1: Yes

Reviewer #2: Yes

**********

5. Is the manuscript presented in an intelligible fashion and written in standard English?

PLOS ONE does not copyedit accepted manuscripts, so the language in submitted articles must be clear, correct, and unambiguous. Any typographical or grammatical errors should be corrected at revision, so please note any specific errors here.

Reviewer #1: Yes

Reviewer #2: Yes

**********

6. Review Comments to the Author

Please use the space provided to explain your answers to the questions above. You may also include additional comments for the author, including concerns about dual publication, research ethics, or publication ethics. (Please upload your review as an attachment if it exceeds 20,000 characters)

Reviewer #1: (No Response)

Reviewer #2: In the revised version, the authors have addressed all the comments. I don't have further comments.

**********

7. PLOS authors have the option to publish the peer review history of their article (what does this mean?). If published, this will include your full peer review and any attached files.

If you choose “no”, your identity will remain anonymous but your review may still be made public.

**Do you want your identity to be public for this peer review?** For information about this choice, including consent withdrawal, please see our Privacy Policy.

Reviewer #1: No

Reviewer #2: No

# Editor report: Acceptance letter

**DOI:** 10.1371/journal.pone.0263341.r004

**Authors:** Feng Gao

2 Feb 2022

PONE-D-21-30277R1 

Development and Evaluation of *AccuPower* COVID-19 Multiplex Real-Time RT-PCR Kit and *AccuPower* SARS-CoV-2 Multiplex Real-Time RT-PCR Kit for SARS-CoV-2 Detection in Sputum, NPS/OPS, Saliva and Pooled Samples. 

Dear Dr. Kim:

I'm pleased to inform you that your manuscript has been deemed suitable for publication in PLOS ONE. Congratulations! Your manuscript is now with our production department. 

If your institution or institutions have a press office, please let them know about your upcoming paper now to help maximize its impact. If they'll be preparing press materials, please inform our press team within the next 48 hours. Your manuscript will remain under strict press embargo until 2 pm Eastern Time on the date of publication. For more information please contact onepress@plos.org.

If we can help with anything else, please email us at plosone@plos.org. 

Thank you for submitting your work to PLOS ONE and supporting open access. 

Kind regards, 

PLOS ONE Editorial Office Staff

on behalf of

Dr. Feng Gao 

Academic Editor

PLOS ONE
